# Fossil *Hyaenanche* Pollen from the Eocene of Kenya: The Paleophytogeograpy and Paleoclimate of a Relict Plant Genus Endemic to the Cape Province, South Africa

**DOI:** 10.3390/biology13121079

**Published:** 2024-12-20

**Authors:** Friðgeir Grímsson, Christian Geier, Johannes M. Bouchal, Silvia Ulrich, Reinhard Zetter, Manuel Vieira

**Affiliations:** 1 Department of Botany and Biodiversity Research, University of Vienna, 1030 Vienna, Austria; christian.geier@univie.ac.at (C.G.); johannes.martin.bouchal@univie.ac.at (J.M.B.); silvia.ulrich@univie.ac.at (S.U.); reinhard.zetter@univie.ac.at (R.Z.); 2 Department of Historical Archaeology, Austrian Archaeological Institute (OeAI), Austrian Academy of Sciences (OeAW), 1010 Vienna, Austria; 3 Department of Earth Sciences, NOVA School of Science and Technology, GEOBIOTEC, Campus de Caparica, 2829-516 Caparica, Portugal

**Keywords:** fynbos, paleobiomes, paleovegetation, palynology, Picrodendraceae, plant dispersal, pollen morphology, rainforest, scanning electron microscopy, tropical vegetation

## Abstract

On the African continent, Picrodendraceae are represented by four genera. Their intracontinental paleophytogeographic histories and paleoecological aspects are obscured by the lack of pre-Miocene fossils. For this study, late Eocene sediments from Kenya were investigated. The sample was prepared in the laboratory, and its organic residue was screened for pollen. We extracted fossil Picrodendraceae pollen and investigated the grains using light and scanning electron microscopy. Based on the pollen morphology, the grains were assigned to *Hyaenanche*. This genus is currently confined to a small area within the Cape Province, South Africa. There, the plants grow as shrubs and small trees at an elevation between 60 and 800 m, on rocky substrate, as part of open fynbos vegetation, and under a dry climate with hot summers and limited precipitation. The sedimentary context and the associated palynoflora suggest that during the Eocene of Kenya, *Hyaenanche* was part of lowland coastal vegetation in Eastern Africa. There, the plants grew under fully humid to winter-dry tropical climates as part of landwards margins of mangroves, seasonally inundated floodplain forests, or coastal forests. Our study shows that when evaluating paleoecological aspects of relict monotypic plants, their extant closely related genera and their fossil records need to be considered.

## Simple Summary

Pollen from angiosperms can have extremely resistant walls that are often preserved as fossils and reflect ancient plants that lived on Earth millions of years ago. We discovered fossil pollen of *Hyaenanche* (Picrodendraceae) in a ca. 37-million-year-old palynoflora from Kenya. Today, *Hyaenanche* is endemic to a small part of the Cape Province, South Africa, where the plants grow on rocky substrate under a dry climate as part of bush vegetation. The Kenyan fossils show that *Hyaenanche* had a much wider distribution in Africa before the present, and compared to other fossil counterparts, we believe that the genus dispersed from Europe into Africa already prior to this earliest record now discovered. The associated fossil pollen and other *Hyaenanche* and alike records suggest that the plants occurred in complex forest environments, enduring fully humid climates in the past. Therefore, our study shows that relying on the closest living or potential modern analogue of a fossil taxon is not always the most representative. This applies especially to relict taxa. For the paleoecological estimations of such fossil plants, their complete fossil records, associated plant taxa from the same fossil assemblages, as well as present day closely related genera need to be considered.

## 1. Introduction

Today, the plant genus *Hyaenanche* Lamb. & Vahl of the Picrodendraceae Small comprises only one living species, *H. globosa* (Gaertn.) Lamb & Vahl. Shrubs and small trees of this taxon are endemic to the Cape Floristic Region (CFR), South Africa [1,2,3]. The main *Hyaenanche* population occurs on the Gifberg of the northern Bokkeveld Escarpment, south of Vanrhynsdorp, in the Northwestern part of the CFR [4]. Despite the limited distribution of this taxon, it is commonly known for its bioactivity and has been used by both indigenous people, the San, for poison on their arrows, and by European newcomers (farmers) to poison carcasses for killing hyenas, jackals, and other animals considered vermin. Both the scientific name *Hyaenanche* (= hyena poison) and the common name gifboom (gif = poison; boom = tree) refer to the plant’s toxic properties used to kill vermin (e.g., [4,5,6]). The *Hyaenanche* trees/shrubs grow under a dry climate with hot summers and limited rainfall confined to winter months. The substrate is rocky, hardground, and/or with shallow and nutrient-poor sandy soil [4,7]. The plants are part of open fynbos vegetation, occurring alongside other typical fynbos trees/shrubs of the genera *Diospyros* L. (Ebenaceae), *Euclea* L. (Ebenaceae), *Heeria* Meisn. (Anacardiaceae), *Leucospermum* R.Br. (Proteaceae), *Maytenus* Molina (Celastraceae), *Olea* L. (Oleaceae), *Osyris* L. (Santalaceae), *Paranomus* Salisb. (Proteaceae), *Phylica* L. (Rhamnaceae), and *Protea* L. (Proteaceae) [4]. For a more detailed description of the South African fynbos, we refer to Cowling et al. [8]. The *Hyaenanche globosa* plants are deciduous shrubs or small trees that are 2 to 4 m tall and up to 8 m in diameter. The leaves are simple and entire and arranged opposite or in whorls of four, and they are lance to elliptic in shape and slightly hairy. The plants are dioecious, but both male and female flowers are small, inconspicuous, and red in color. The staminate flowers have 8–30 stamens [2,4]. *Hyaenanche* produces ± spherical, echinate, and stephanoporate pollen characteristic of the Picrodendraceae [9,10,11,12,13]. The fossil record of this genus is meagre. Until now, only a single African locality with sediments of early Miocene age has yielded *Hyaenanche* pollen [13]. The other few pollen records are all from the early to late Eocene of Europe (e.g., [13,14,15]).

Here, we describe fossil *Hyaenanche* pollen grains from the late Eocene of Kenya, Eastern Africa, the earliest record of this genus from the African continent. The discovered fossil pollen is compared to that from present-day *Hyaenanche globosa*, endemic to South Africa, and to similar pollen of the closely related genera *Tetracoccus*, *Picrodendron*, and *Piranhea* of the Picrodendraceae, occurring in the Americas and Caribbean. The complete fossil record of *Hyaenanche* and similar pollen is compiled and compared to the new Eocene pollen from Kenya. Based on morphological comparison between the fossil pollen from America, Europe, and Africa, the early Cenozoic origin followed by an Eocene dispersal of this lineage into Africa is further elaborated. Also, by examining the current habitats and climate tolerances of extant American and African Picrodendraceae, along with the associated paleofloras comprising the fossil *Hyaenanche* pollen, we estimate the palaeoecological aspects of *Hyaenanche* from the Eocene of Kenya. Furthermore, we speculate how the climate/habitat preferences of this genus have changed/narrowed up through the Cenozoic.

## 2. Materials and Methods

### 2.1. Origin and Preparation of Sample

**Figure 1:**
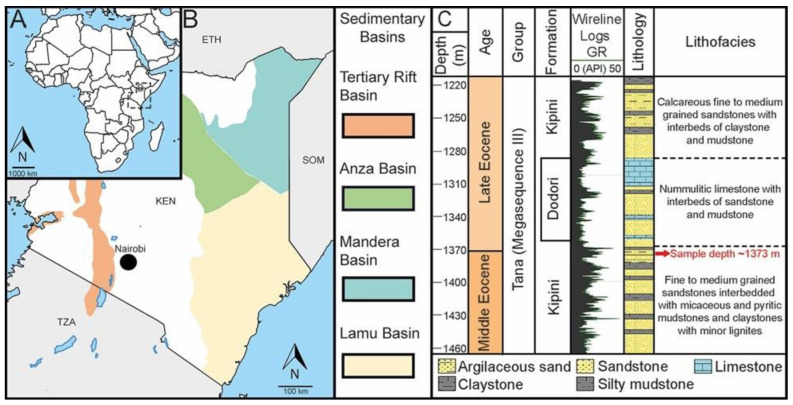
Geographic and geological maps. (**A**). Schematic map of the African continent showing the position of the study site, Kenya, Eastern Africa. (**B**). Schematic map showing major relevant geological formations and the location of the Dodori-1 well, close to Dodori, southeastern Kenya. (**C**). Compiled stratigraphic profile (modified after Vieira et al. [18]) showing the stratigraphic level of the sample comprising the fossil Picrodendraceae pollen. KEN = Kenya; TZA = Tanzania; ETH = Ethiopia; SOM = Somalia.

The fossil *Hyaenanche* pollen grains from Kenya, Eastern Africa (Figure 1), were extracted from a single core sample of the Dodori-1 drill core (lat. 1° 48′ 53.7” S, long 41° 11′ 04.0″ E), Lamu Basin, Kenya, Africa [16]. The core was named after a settlement in Lamu County situated c. 21 km inland from the coast (Figure 1). The Dodori-1 core was drilled in 1964 to a depth of c. 4340 m, penetrating both Cretaceous and Cenozoic strata, at the periphery of the Lamu Basin. The sample we studied is from a silty mudstone, initially positioned ca. 1373 m below the surface. The sample was processed, and fossil pollen was extracted according to methods explained in Halbritter et al. [17], (pp. 118–121). Consequently, fossil *Hyaenanche* pollen grains were investigated with both light microscopy (LM) and scanning electron microscopy (SEM) using the single]-grain method, as described by Halbritter et al. [17], (pp. 121–123). SEM stubs with the fossil *Hyaenanche* pollen from Kenya investigated for this study and part of the original sedimentary sample are stored in the collection of the Department of Botany and Biodiversity Research, University of Vienna, Austria.

### 2.2. Geographic and Geological Background

The Lamu Basin, the largest basin in Kenya, spans southeastern Kenya and extends into southwestern Somalia (Figure 1) [19]. Between the 1950s and the early 2000s, 16 wells were drilled in the Lamu Basin to pursue hydrocarbon resources. While these drilling efforts did not result in successful discoveries, they contributed significantly to understanding the basin’s geology. The basin fill comprises a diverse range of rocks, spanning from the Permian to the Cenozoic, including continental rift sandstones, fluvio-deltaic sandstones, marine shales, and carbonates. The Mesozoic and Cenozoic sediments of the Lamu Basin have been categorized into four megasequences deposited during alternating transgressive and regressive periods [16]. Megasequence I, known as the Karroo Group, includes sediments from the Late Permian to Middle Jurassic. Megasequence II, referred to as the Sabaki Group, encompasses strata laid down between the Late Jurassic and Late Paleocene. Megasequence III, the Tana Group, consists of Paleogene sediments (Eocene–Oligocene) that sometimes rest on a regional unconformity above the Sabaki Group. The Tana Group includes the Kipini Formation (Fm) and the Dodori Limestone (Figure 1), deposited during three sea-level rise events and one regressive phase. Megasequence IV, or the Coastal Group, represents the most recent sedimentary deposits of the Lamu Basin, lying unconformably on top of the late Oligocene sediments of the Tana Group [16]. The Kipini Fm (Tana Group) is fairly extensive, covering the southern part of the Lamu Basin, extending from the early Eocene into the late Oligocene. It is characterized by interbedded nummulitic sands, poorly sorted calcareous sandstones, nummulitic and micritic limestones, along with dark olive greenish-grey shales and grey-green silty mudstones. All lithologies are interpreted as having formed within a delta-front environment [16]. The Dodori Limestone represents carbonate shelf-deposition, which occurred until the late Eocene during a transgressive phase. This deposition was intermittently disrupted by the progradation of siliciclastic deposits associated with the Kipini Fm. The Dodori Limestone features nummulitic components, shell fragments, and interbedded mudstones [16]. The pollen sample originates from a greyish shale within the Kipini Fm, located just beneath the Dodori Limestone, at a depth of ca. 1373 m (Figure 1). This interval has been dated to the earliest late Eocene (earliest Priabonian) at approximately 37.71 Ma (according to Cohen et al. [20], updated). This dating is supported by the consistent presence of the large foraminifera *Nummulites fabianii* (found from 1344 m upwards) and various dinoflagellate cyst species, including *Cordosphaeridium fibrospinosum*, which are characteristic of the late Eocene.

### 2.3. Criteria for Applicable Fossil Pollen Records

Previously published paleopalynofloras, as well as other paleobotanical essays/theses on fossil flowers with in-situ pollen from America, Europe, Africa, and Asia, were screened for pollen falling within the morphological variability of *Hyaenanche* (and alike) of the Picrodendraceae, as outlined by, e.g., Punt [9], Köhler [11], Simpson and Levin [12], Grímsson et al. [13], and Levin and Simpson [21]. All fossil pollen showing shared features currently occurring in extant *Hyaenanche* and closely related taxa were measured and described based on published micrographs or previous descriptions. The compiled fossil record was plotted onto a map noting the fossils’ age and their approximate location. These data were used to hypothesize on the origin of the Picrodendraceae, how they diverged, and which lineage dispersed into Africa.

### 2.4. Georeferenced Biome and Climate Data: Harvesting and Analysis

For extant *Hyaenanche* and closely related American genera, georeferenced distribution data were downloaded from GBIF.org (*Hyaenanche*: https://doi.org/10.15468/dl.52hh3x (accessed on 12 September 2024); *Picrodendron*: https://doi.org/10.15468/dl.ee6dmh (accessed on 5 November 2024); *Piranhea*: https://doi.org/10.15468/dl.eeaucq (accessed on 12 September 2024); *Tetracoccus*: https://doi.org/10.15468/dl.gzmzaf (accessed on 5 November 2024)). These data sets were corrected for outliers (e.g., records from botanical gardens were removed) and multiple occurrences (same coordinates were merged). The adjusted data sets include 65 georeferenced occurrences of the monotypic *Hyaenanche*, 75 occurrences of the monotypic *Picrodendron*, 393 occurrences of *Piranhea* (four extant species), and 1059 occurrences of *Tetracoccus* (five extant species) (Supplementary Materials File S1). These data sets were then plotted, using the “Intersection geoprocessing” tool in Qgis (v.3.34.11-Prizren), onto the world biome data set of Olson et al. [22] to compile biome profiles for the investigated genera/species. Köppen profiles were used to summarize the climatic niches occupied by living *Hyaenanche*, *Picrodendron*, *Piranhea*, and *Tetracoccus* species and to evaluate potential niche shifts within this American–African clade of *Picrodendraceae* through geological time. For clarification, Köppen profiles reflect the proportional Köppen–Geiger climate (e.g., [18,23]) zone coverage of modern plant species based on their gridded distribution data from GBIF.org (Supplementary Material File S2; [24,25,26]). The occurrence data sets were then plotted onto 1 km^2^ grid Köppen–Geiger climate maps (data covering the years 1979–2013, from Cui et al. [27]) to establish Köppen profiles for the genera/species studied. The georeferenced data and the Köppen–Geiger climate maps, with 1 km^2^ resolution, were processed using the ‘Sample Raster Values’ Toolbox in Qgis v.3.16.4-Hannover. The Köppen–Geiger climates occupied by extant *Hyaenanche*, *Picrodendron*, *Piranhea*, and *Tetracoccus* are provided as maps generated in Qgis and as frequency diagrams (showing proportional distribution) (Supplementary Materials File S2). Additionally, climate diagrams covering mean monthly temperatures (MMTs), maximum monthly temperatures (MaxMTs), minimum monthly temperatures (MinMTs), and the average monthly precipitation (MMP) for all extant *Hyaenanche*, *Picrodendron*, *Piranhea*, and *Tetracoccus* species are provided (see Supplementary Materials File S2). These were compiled relying on historical climate data, from the years 1970–2000, using World clim v.2.1 (https://www.worldclim.org/data/worldclim21.html (accessed on 26 November 2021) at a resolution of 30 s (c. 1 km^2^; [28]).

## 3. Results and Discussion

### 3.1. Systematic and Descriptive Palynology

The plant taxonomy to family level follows APG IV [29]. The pollen description includes all morphological and diagnostic features observed with both LM and SEM. The pollen terminology follows Punt et al. [30] (LM) and Halbritter et al. [17] (LM and SEM).

Clade: Angiosperms

Order: Malpighiales Juss. ex Bercht. & J.Presl

Family: Picrodendraceae Small

Genus: *Hyaenanche* Lamb. & Vahl

**Figure 2:**
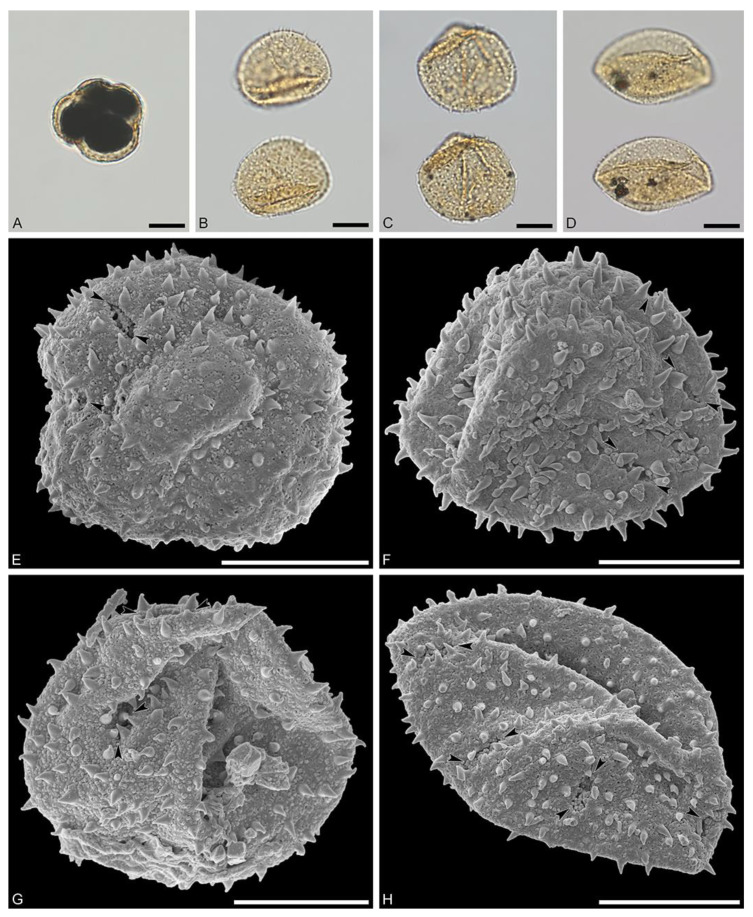
Fossil *Hyaenanche* Dodori MT pollen from the earliest late Eocene of southeast Kenya, Africa. (**A**–**D**). LM micrographs. (**E**–**H**). SEM micrographs. (**A**). Oblique polar view, pollen filled with pyrite. **B**. Oblique equatorial view, compressed grain. (**C**). Oblique polar view, compressed grain. (**D**). Oblique view, folded grain. (**E**). Oblique polar view, same grain as in (**A**), note apertures and their membranes. (**F**). Oblique equatorial view, same grain as in (**B**), nanogemmae less conspicuous. (**G**). Oblique polar view, same grain as in (**C**), apertures infolded. (**H**). Oblique polar view, compressed grain, same as in (**D**), note row of apertures along equatorial plane. Black arrowheads pinpoint apertures. Scale bars = 10 µm.

**Figure 3:**
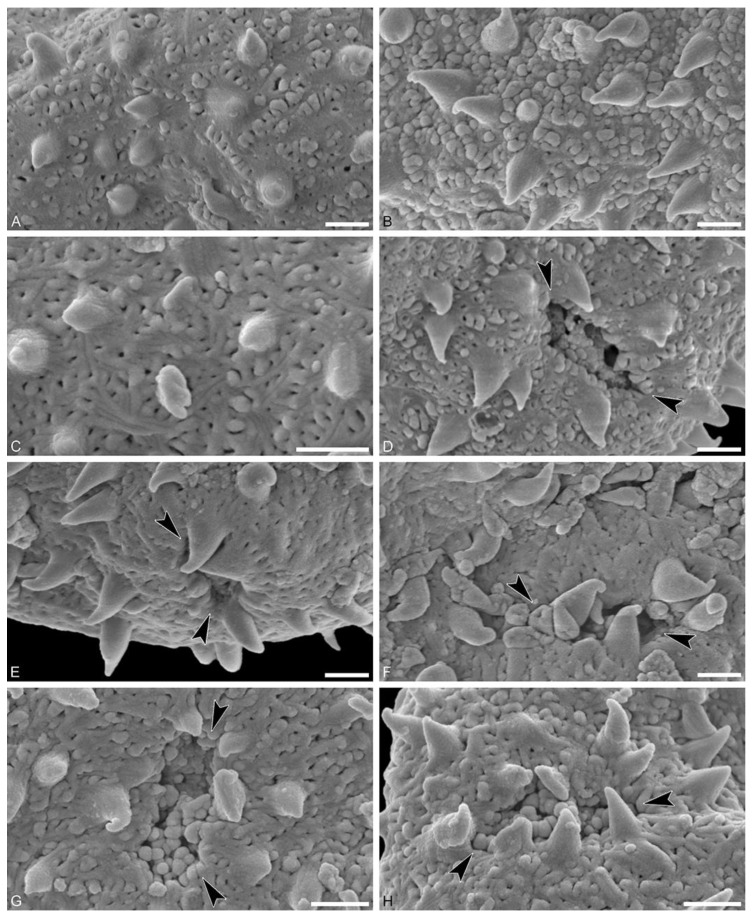
Fossil *Hyaenanche* Dodori MT pollen from the earliest late Eocene of southeast Kenya, Africa. (**A**–**H**). SEM micrographs. (**A**). Interapertural area (close-up of Figure 2A), fossulae and large perforation in-between echini, nanogemmae in rows and widely spaced. (**B**). Interapertural area (close-up of Figure 2G), nanogemmae densely packed, echini striate. (**C**). Interapertural area (close-up of Figure 2H), fossulae and large perforations in-between echini, nanogemmae less conspicuous. (**D**). Aperture region (close-up of Figure 2E), parts of membrane preserved. (**E**). Aperture region, note echini bending over pori. (**F**). Aperture region (close-up of Figure 2F), aperture infolded and obscured by echini. (**G**). Aperture region (close-up of Figure 2H), aperture membrane nanogemmate. (**H**). Aperture region (close-up of Figure 2H), aperture membrane nanogemmate, echini bending over pori. Black arrowheads pinpoint apertures. Scale bars = 1 µm.

Species: *Hyaenanche* sp., Dodori morphotype (MT) (Figure 2 and Figure 3)

Description: Pollen, monad, isopolar, P/E ratio isodiametric to slightly oblate, shape spheroidal, outline circular to slightly elliptic in polar and equatorial view; equatorial diameter including echini 23–33 μm in LM, 21–30 μm in SEM, equatorial diameter excluding echini 21–31 μm in LM, 19–28 μm in SEM, polar axis including echini 20–21 μm in LM, 18–19 μm in SEM, polar axis excluding echini 19–20 μm in LM, 17–18 μm in SEM; stephano(6–7)porate; pori at regular intervals, positioned at the equator, elliptic in outline, 1.7– 3.2 μm in diameter (SEM); exine 1.2–1.4 μm thick; pollen wall tectate; sculpture echinate in LM, microechinate to echinate in SEM, and fossulate, perforate, and nanogemmate in areas between echini (SEM); 26–42 echini per 100 μm^2^ pollen surface in central polar area; echini at irregular intervals, 0.6–1.7 μm in height, echini broad at base and narrowing abruptly at around 2/3 of their height, faintly striate especially at base; aperture membrane nanogemmate (SEM).

Remarks: Picrodendraceae pollen from all extant genera has been investigated both morphologically (LM and SEM) and ultrastructurally (TEM) by Punt [9], Köhler [11], Simpson and Levin [12], Grímsson et al. [13], Levin and Simpson [21]*,* and references therein. Extant and relevant fossil Picrodendraceae pollen are listed in Table 1, Table 2, Table 3 and Table 4. 

Compared to available micrographs of extant Picrodendraceae pollen, the fossil Dodori MT from the Eocene of Kenya resembles mostly pollen from *Piranhea* and *Hyaenanche*. Pollen of these two genera is morphologically similar, but differs slightly in size, shape, and density of echini (Table 1). *Piranhea* has between 12 and 20 echini per 100 μm^2^, with a height of 1.6–2.7 µm and a cone-like shape. *Hyaenanche* has 15–30 echini per 100 μm^2^, with a height of 1.0–2.0 µm and a sturdier shape at the base and tapers off in the upper part of the echinus. 

Upon closer comparison with pollen of *H. globosa*, the affinity of the Dodori MT pollen to this taxon becomes apparent (compare Figure 2 and Figure 3 with Köhler ([11], (pl. 8, fig. 18)), Punt ([10], (fig. 7)), Simpson and Levin ([12], (figs. 54 and 55)), and Grímsson et al. ([13], (fig. 6)). The fossil Dodori MT pollen has a slightly shorter polar axis and equatorial diameter than observed in pollen of extant *H. globosa* (up to 1/3 shorter). These differences might both come from biological reasons as well as taphonomic processes like compression of the fossil pollen during sediment accumulation and fossilization. Additionally, the pori of the fossil Dodori MT pollen are slightly smaller than observed for pollen of the extant species (1.7–3.2 µm vs 3.0–5.5 μm). The exine sculpture is essentially the same (echinate and fossulate, perforate and nanogemmate between echini, and with finely striate echini) except for the density and height of the echini. The fossil Dodori MT pollen has more and shorter echini (26–42 echini per 100 μm^2^ pollen surface, with a height of 0.6–1.7 µm) versus pollen of *H. globosa* (15–30 echini per 100 μm^2^ pollen surface, with a height of 1.0–2.0 μm). However, both the number of echini and their size measures are overlapping.

### 3.2. Ecological Aspects of Early Divergent American and African Picrodendraceae as Proxy for Fossil Eocene Taxa

**Figure 4:**
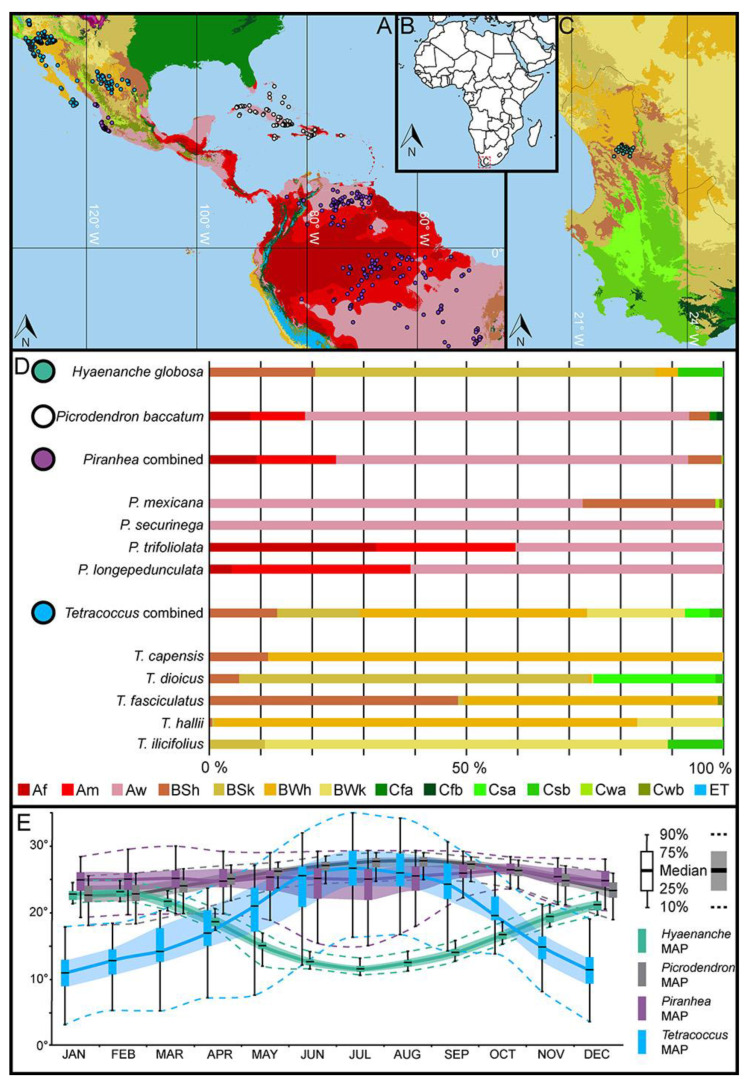
Geographic distribution and climate parameters for *Hyaenanche* and relevant taxa. (**A**–**C**). Geographic distribution in relation to Köppen climate classification. (**D**). Köppen climate profiles. (**E**). Minimum monthly temperatures (MinMTs). The climate map was generated in Qgis based on Cui et al. [27]. The distribution and climate profiles for each species are available in the Supplementary Materials File S2. Af = fully humid equatorial rainforest, Am = equatorial monsoonal, Aw = winter dry equatorial savannah, BSh = hot arid steppe, BSk = cold arid steppe, BWh = hot arid desert, BWk = cold arid desert, Cfa = fully humid warm temperate with hot summer, Cfb = fully humid warm temperate with warm summer, Csa = summer dry warm temperate with hot summer, Csb = summer dry warm temperate with warm summer, Cwa = winter dry warm temperate with hot summer, Cwb = winter dry warm temperate with warm summer, and ET = polar tundra climates.

**Figure 5:**
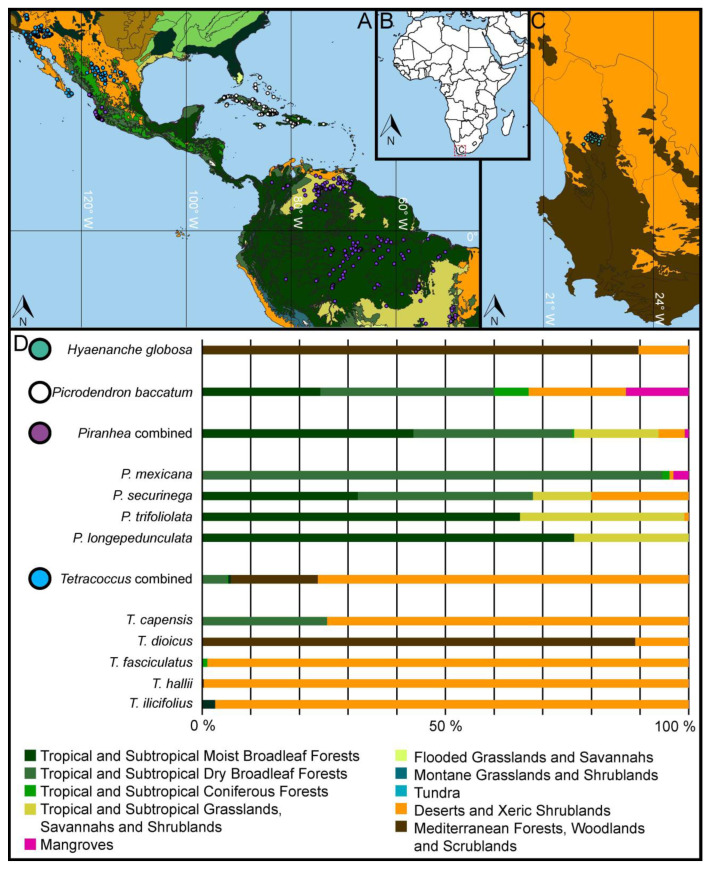
Geographic distribution and Biome parameters for *Hyaenanche* and relevant taxa. (**A**–**C**). Geographic distribution in relation to Biomes. (**D**). Biome profiles (proportional occupied biomes). The Biome map was generated in Qgis based on Olson [22]. The distribution and Biome profiles for each species are available in the Supplementary Materials File S2.

All currently living *Hyaenanche*, *Picrodendron*, *Piranhea*, and *Tetracoccus* species thrive under warm climates in the Americas or Africa (Figure 4 and Figure 5). *Hyaenanche globosa* has a very limited distribution in South Africa, occurring in areas enduring hot steppe (BSh) or cool steppe (BSk) climates, but it is also found growing in habitats with hot desert (BWh) and temperate summer dry (Csb) climates (Figure 4). During the warmest period, between November and March (dry season), the precipitation drops below 15 mm per month in the main distribution area of this species (Supplementary Materials File S2). The foremost biome where *H. globosa* occur is the Mediterranean Forests, Woodlands and Scrublands Biome, but some isolated populations also occur within the Deserts and Xeric Shrublands Biome (Figure 5).

*Picrodendron baccatum* are small trees and shrubs restricted to the Caribbean [31,43]. The plants occur primarily in fully humid (Af), monsoonal (Am), and winter dry (Aw) tropical climates. At the periphery of their distribution, they extend into hot steppe (BSh) climates and, in mountainous areas, into fully humid temperate (Cfa, Cfb) climates. During the dry period, between December and March, the precipitation drops below 50 mm per month in the main distribution area of this species (Supplementary Materials File S2). The main habitats of *P. baccatum* are the Tropical and Subtropical Moist Broadleaf Forests, Tropical and Subtropical Dry Broadleaf Forests, and the Tropical and Subtropical Coniferous Forests Biomes. However, the species also extends into the Deserts and Xeric Shrublands and Mangroves Biomes (Figure 5). *Picrodendron* plants are calciphile, often growing on limestone and as part of dry limestone shrub forests. They also have some salt tolerance and are found in the landward margins of mangroves [31].

*Piranhea* comprises shrubs and trees, mainly thriving under fully tropical (Af, Am, Aw) climates but can extend into arid steppe climates (BSh), as well as winter-dry warm temperate (Cwa, Cwb) climates (Figure 4). Generally, these correspond to several different tropical and subtropical forest vegetation types that all fall within the Tropical and Subtropical Grasslands, Savannahs and Shrublands Biome, the Deserts and Xeric Shrublands Biome, and the Mangroves Biome (Figure 5). *Piranhea longepedunculata* is confined to fully tropical climates, including mainly summer dry (As) and monsoonal (Am), but at the periphery of its range, it extends into fully humid (Af) climates (Figure 4). Between July and September (dry season), the precipitation drops below 10 mm per month in the main distribution area of *P. longepedunculata* (Supplementary Materials File S2).

Piranhea longepedunculata is exclusive to the Tropical and Subtropical Moist Broadleaf Forests Biome and the Tropical and Subtropical Grasslands, Savannahs and Shrublands Biome. In Brazil, P. longepedunculata is a member of well-drained forests and seasonal floodplain forests inundated by rivers across the Brazilian states of Pará, Rondônia, and Maranhão [44]. Piranhea mexicana is restricted to Central America, where its main distribution is traced along Mexico’s Pacific coast. The climate preferences of P. mexicana range from fully tropical winter dry climates (Aw) to hot steppe climates (BSh), but the plants also occur in montane regions enduring winter dry temperate climates with hot (Cwa) and warm summers (Cwb) (Figure 4). In the main distribution area, the precipitation drops below 10 mm per month between February and May (dry season), coinciding with the lowest minimum monthly temperatures (Supplementary Materials File S2). This species mainly occurs in the Tropical and Subtropical Dry Broadleaf Forests Biome but extends into the Tropical and Subtropical Coniferous Forests Biome in montane regions and also into the Mangroves Biome along coastlines. In the Mexican states of Jalisco, southern Sinaloa, and in northern Nayarit, P. mexicana is a member of lowland deciduous forests and mixed coastal forests at higher elevation. In these forests, P. mexicana can be very abundant at localized sites [45]. Piranhea securinega is distributed across the Bahia and Minas Gerais states of western Brazil, in areas enduring fully tropical winter dry climates (Aw) (Figure 4). In this region, the dry season occurs between June and August, with the precipitation dropping below 10 mm per month (Supplementary Materials File S2). This coincides with the coolest monthly temperatures (MinMTs) (Figure 4). Piranhea securinega is spread across four different biomes, occurring in the Tropical and Subtropical Moist Broadleaf Forests Biome, the Tropical and Subtropical Dry Broadleaf Forests Biome, the Tropical and Subtropical Grasslands, Savannahs and Shrublands Biome, and the Deserts and Xeric Shrublands Biome. In the states of Bahia, Goiás, and Minas Gerais, P. securinega is a part of semi-arid tropical vegetation as well as mixed ombrophilous forests [44]. Piranhea trifoliolata has the widest distribution within the genus, spanning from latitude 10° N to 16° S across South America. The species is restricted to fully tropical climates, including fully humid (Af), monsoonal (Am), and winter dry (Aw) conditions (Figure 4). Piranhea trifoliolata primarily inhabits the Tropical and Subtropical Moist Broadleaf Forests Biome and the Tropical and Subtropical Grasslands, Savannahs and Shrublands Biome, but with rare occurrences in the Deserts and Xeric Shrublands Biome. In the Amazon, P. trifoliolata inhabits floodplains and seasonally inundated forests [46].

Tetracoccus comprises xerophytic shrubs occurring in the Southwestern USA and Mexico [47]. The shrubs primarily grow under arid steppe (BSh, BSk) and desert (BWh, BWk) climates, but can also be found under summer dry warm temperate (Csa, Csb) climates (Figure 4). The plants mainly inhabit the Deserts and Xeric Shrublands, the Mediterranean Forests, Woodlands and Scrublands, and the Tropical and Subtropical Dry Broadleaf Forests Biomes, but rarely extend into the Tropical and Subtropical Moist Broadleaf Forests and the Tropical and Subtropical Coniferous Forests Biomes (Figure 5). Tetracoccus capensis shrubs are restricted to the Cape Region, Baja California, where they endure hot arid steppe (BSh) and desert (BWh) climates (Figure 4). The plants are exclusively found in the Deserts and Xeric Shrublands and the Tropical and Subtropical Dry Broadleaf Forest Biomes (Figure 5). In this region, the precipitation drops below 10 mm per month between February and June (Supplementary Materials File S2). Tetracoccus dioicus shrubs occur in Orange and San Diego counties, USA, and Northern Baja California, Mexico [47,48]. The plants grow under hot and cold arid steppe (BSh), temperate summer dry (Csa, Csb), and occasionally in hot and cold arid desert (BWh, BWk) climates (Figure 4). May until October are the driest months, with precipitation below 20 mm (Supplementary Materials File S2). This species primarily occurs in the Mediterranean Forests, Woodlands and Scrubland and the Deserts and Xeric Shrublands Biomes (Figure 5). The plants are usually part of coastal chaparral and coastal scrub vegetation, at an elevation between 100 and 700 m [48]. Tetracoccus fasciculatus distribution extends from the Central Mexican Plateau (the states Chihuahua and Durango) to the Sierra Madre Oriental Mountain range (state of Coahuila) [47]. The plants thrive primarily in hot arid steppe (BSh) and desert (BWh) climates, but occasionally extend into cold steppe (BSk) and winter dry (Cwb) climates (Figure 4). November until April are the driest months, with precipitation below 20 mm (Supplementary Materials File S2). The main habitats of T. fasciculatus are within the Deserts and Xeric Shrublands Biome, with rare occurrences in the Tropical and Subtropical Coniferous Forests Biome (Figure 5). The scrubby plants grow at an elevation between 1000 and 2000 m and are part of desert and chaparral habitats [47]. Tetracoccus halli are small shrubs occurring in the southern part of California, Arizona, and Nevada, USA, and Baja California, Mexico [47,48]. The plants predominantly grow under hot or cold arid desert (BWh, BWk) climates and occasionally extend into temperate summer dry (Csa) climates (Figure 4). The monthly precipitation rarely exceeds 40 mm in the distribution region of this species (Supplementary Material File S2). Tetracoccus halli only occurs in the Deserts and Xeric Shrublands Biome (Figure 5). The shrubby plants grow on desert slopes and dry washes at an elevation between 30 and 1200 m [48]. Tetracoccus ilicifolius has the most restricted distribution within the genus and only occurs on mountain sides flanking Death Valley, CA, USA [47,48]. The plants grow under cold arid steppe (BSk), desert (BWk), as well as temperate summer dry (Csa) climates (Figure 4). As with the previous species, T. ilicifolius grows predominantly in areas receiving less than 40 mm of monthly precipitation (Supplementary Materials File S2). The habitats of T. ilicifolius all belong to the Mediterranean Forests, Woodlands and Scrubland and the Deserts and Xeric Shrublands Biomes (Figure 5). Tetracoccus ilicifolius grows on limestone outcrops in desert scrub vegetation, at an elevation between 600 and 1900 m [48].

### 3.3. The Hyaenache Dodori MT Pollen Compared to That of Extant and Fossil American-Afro-Indian Clade Picrodendraceae

**Figure 6:**
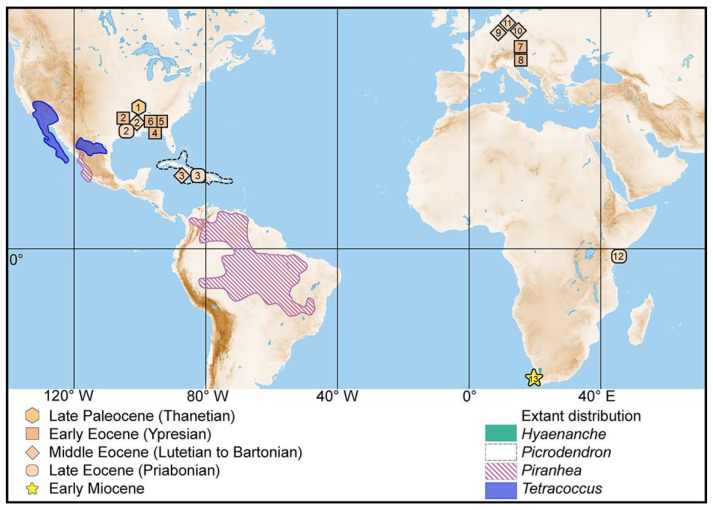
Fossil records and extant distribution of *Hyaenanche* and relevant taxa. For fossil taxa, consult records listed in Table 1, Table 2, Table 3 and Table 4.

All Picrodendraceae genera produce pollen that is ±spheroidal, with a porate aperture configuration and echinate sculpture. Still, based on the pollen morphology observed with combined LM and SEM, it is possible to differentiate genera or groups of taxa within the family. The three American genera of the American-Afro-Indian clade all produce similar pollen that can be differentiated based on SEM scrutiny. Not only can pollen of Tetracoccus, Picrodendron, and Piranhea be differentiated from each other (Table 1, Table 2, Table 3 and Table 4), but also from the fossil Hyaenanche Dodori MT pollen. The fossil pollen is considerably smaller than that of Tetracocccus ([13], (fig. 14)), the apertures are also shorter/smaller, and the exine is considerably thinner. The number of echini per 100 µm^2^ pollen surface is much higher in the fossil pollen than in Tetracoccus (Table 1, Table 2, Table 3 and Table 4). Most importantly, the sculpture elements in-between the echini are very different when the two taxa are compared. Tetracoccus pollen has densely packed nanogemmae and only rare and tiny perforations. On the contrary, the Hyaenanche Dodori MT pollen has widely spaced nanogemmae and numerous distinct perforations as well as fossulae. This particular difference can also be observed when Picrodendron baccatum pollen ([31], (figs. 3–9)) is compared to the fossil Hyaenanche Dodori MT pollen. In addition, there seems to be a higher variability in the number of pori in Picrodendron pollen compared to the fossil Dodori MT, and the echini in P. baccatum pollen are larger (Table 1, Table 2, Table 3 and Table 4). Out of the three relevant American genera, the pollen of Piranhea ([13], (fig. 11 and 12)) is most comparable to that of the fossil Hyaenanche Dodori MT pollen. The size of Piranhea pollen is similar to that of the fossil Dodori MT pollen and they share the same number and arrangement of pori. The apertures can be smaller/shorter in the fossil Dodori MT pollen than in Piranhea, and the exine is thinner in the fossil pollen. The number of echini per 100 µm^2^ pollen surface is higher in the fossil Dodori MT, but their configuration is comparable, and the sculpture elements in-between the echini are similar. Still, the nanogemmae are more densely packed in pollen of Piranhea than in the fossil Dodori MT; the perforations are also distinct but not to the degree and frequency as noted for the fossil Dodori MT pollen. Additionally, there are also fossulae between the nanogemmae and perforations in pollen of Piranhea as in the fossil Dodori MT pollen (Table 1, Table 2, Table 3 and Table 4).

For the Afro-Indian Picrodendraceae, Grímsson et al. [13] established three pollen types (PTs). All three PTs have 5–8 pori situated around the equator and are stephanoporate. The pollen of *Hyaenanche globosa* was categorized as PT1, the only African taxon producing such a PT. The combination of pollen features that makes it unique within the Afro-Indian clade are a high number (15–30 per µm^2^) of medium sized (1–2 µm) echini paired with a fossulate, perforate, and nanogemmate ornamentation in areas between echini. In addition, the aperture membrane is nanogemmate. All these features correspond well to those observed in the fossil Dodori MT pollen (Table 1, Table 2, Table 3 and Table 4). PT2, within Afro-Indian Picrodendraceae, occurs in *Aristogeitonia*, *Mischodon*, *Oldfieldia*, and *Voatamalo*. Pollen from these taxa share the general Picrodendraceae pollen morphology, but with the most striking feature being large echini (2–4.5 µm) with a density of 3–13 per 100 µm^2^ pollen surface. These features exclude the aforementioned genera as potential modern analogues or nearest living relatives of the parent plant producing the fossil Dodori MT pollen, which has shorter and more densely arranged echini (Table 1, Table 2, Table 3 and Table 4). The pollen of *Androstachys* and *Stachyandra* was categorized as PT3 by Grímsson et al. [13]. The pollen from these two genera is slightly angular, microechinate, granulate, and perforate, and the echini measure between 0.7 and 1.3 µm in height and occur at a density of 30–45 per 100 µm^2^ pollen surface. The aperture membrane is microechinate. Still, the most defining characteristic of PT3 are the irregularly placed pori, which not only occur around the equator but are also dislocated onto the hemispheres of the pollen grains. Due to the presence of several (micro)echini (26–45 echini per 100 μm^2^) with a height of 0.6–1.7 µm, the fossil Dodori MT pollen is similar to extant pollen of category PT3. However, the PT3 pollen grains have pori placed at irregular intervals and dislocated outside of the equator, which does not occur in the fossil Dodori MT pollen. In addition, the exine sculpture of PT3 is granulate and perforate, versus fossulate, perforate, nanogemmate, and striate in the Dodori MT pollen. Finally, the PT3 and the Dodori MT pollen differ in ornamentation of the aperture membrane. The former is mostly nanoechinate, nanogemmate, and granulate, while the fossil Dodori MT pollen has only a nanogemmate aperture membrane. In short, out of all the American-Afro-Indian clade Picrodendraceae pollen, the fossil Dodori MT compares best to that of extant *Hyaenanche globosa*.

Even though the fossil Paleocene and Eocene American Picrodendraceae pollen have features that clearly define them as members of this family, their morphology is more comparable to pollen from extant *Tetracoccus* and *Picrodendron* than to the fossil *Hyaenanche* Dodori MT (Table 1, Table 2, Table 3 and Table 4). Micrographs from the available literature suggest that the fossil American pollen grains originate from more than a single biological species, and when compared, they can be differentiated using LM- and SEM-based morphology. Among the features that differentiate the Paleocene to Eocene American pollen types (Table 1, Table 2, Table 3 and Table 4) from the Dodori MT pollen are displaced apertures, distinct exine thickenings around apertures, larger and widely distributed echini, and the configuration of sculpture elements in-between echini. The Dodori MT pollen is very similar to fossil *Piranhea*/*Hyaenanche* pollen types from the Early and Middle Eocene of Europe (Table 1, Table 2, Table 3 and Table 4). The European fossils, from the Early Eocene of Austria and Middle Eocene of Germany completely match the Dodori MT pollen in all morphological characteristics. The European and the Dodori MT pollen have the same size, the same P/E ratio, and are identical in outline and in aperture configuration. The aperture diameter can be slightly larger in the Dodori MT pollen, but its exine thickness is overlapping, the ornamentation and sculpture elements in-between the echini are comparable, the number of echini per 100 µm^2^ pollen surface is within the range, and the height of the echini is similar. In short, it is very hard to distinguish between an Early to Middle Eocene European *Piranhea*/*Hyaenanche* pollen and that from the Early Late Eocene of Dodori, Kenya, Eastern Africa (Table 1, Table 2, Table 3 and Table 4). The Dodori MT pollen is also similar to the Early Miocene Saldanha MT pollen from Saldanha Bay, South Africa (Table 1, Table 2, Table 3 and Table 4). The two MTs overlap in size, P/E ratio, outline, aperture configuration, and sculpture. Still, the diameter of apertures in the Saldanha MT pollen is larger, and the echini can also be higher. In addition, the Dodori MT pollen grains have more echini per 100 µm^2^ pollen surface. Out of all the American, European, and African fossil Picrodendraceae pollen (Table 1, Table 2, Table 3 and Table 4), the Dodori MT pollen from Kenya is most similar to Early and Middle Eocene European *Piranhea*/*Hyaenanche*-like pollen and second to the Saldanha MT pollen from the Early Miocene of South Africa. The morphology of the Saldanha MT pollen is again very close to that of pollen from extant *Hyaenanche globosa* (Table 1, Table 2, Table 3 and Table 4).

### 3.4. Palephytogeography of Early Diverging American-African Picrodendraceae

Molecular phylogeny places American (excl. *Podocalyx*) and African Picrodendraceae within an American-Afro-Indian clade ([13], (figs. 24 and 25)), with *Tetracoccus* as the earliest diverging genus. *Picrodendron* and *Piranhea* appear as sister genera in the second diverging lineage of the same clade. These are followed by Afro-Indian genera, with *Hyaenanche* as the earliest diverging genus within the Afro-Indian clade. The phylogeny shows that African Picrodendraceae are most closely related to present-day American taxa, and that the lineage that dispersed into Africa originated from a North American stock. This pattern is also supported by the fossil record (Figure 6). The earliest fossils representing the Picrodendraceae lineage leading to the African-Indian clade are late Paleocene flowers with in-situ pollen from Buchanan, Tennessee, USA, assigned to the Arecaceae by Feldman [32] (Figure 6 and Table 1, Table 2, Table 3 and Table 4). Based on the SEM and TEM micrographs of the pollen provided by the author, these pollen grains are not sulcate but stephanoporate and of clear Picrodendraceae affinity, as suggested by Grímsson et al. [13], having a suite of features that occur in extant pollen of *Tetracoccus*, *Piranhea*, and *Picrodendron*, the three basal genera within the American-Afro-Indian clade. These shared pollen features highlight the potential ancestral state of the fossil Paleocene pollen from Tennessee. However, further detailed analysis of the fossil flowers and their in-situ pollen in comparison to that of extant North American Picrodendraceae taxa are needed to fully understand the intrafamilial position of the Tennessee fossils. The Paleocene fossils are followed by a considerably higher number of Eocene records from the Americas, Europe, Australasia, and now also Africa (this study). The fossil Australasian Picrodendraceae pollen records are not part of the evolutionary lineage leading to African genera and are therefore not discussed herein. Early to Late Eocene pollen records from the Mississippi and Alabama area (e.g., *Echiperiporites* [33,37]; *Malvacipollis* [37,49]; *Nothofagus* [39]) and from the Middle to Late Eocene of Cuba (*Malvacipollis tschudyi* [35]), with morphological suites suggesting affinities to *Picrodendron* and *Piranhea*, show that the Picrodendraceae had already diverged and dispersed within North America during the Paleocene and Eocene. The further Early Cenozoic fossil record of the American-Afro-Indian clade is represented by several Early, Middle, and Late Eocene pollen (Figure 6, Table 1) with affinity to both *Piranhea* and *Hyaenanche* from Austria [13,14,15] and Germany [13,41,42]. These European fossil records, in combination with the molecular phylogeny, suggest that Picrodendraceae dispersed from North America to Europe across the North Atlantic Land Bridge during the Early Eocene. This dispersal route is a known gateway for many temperate to tropical plant taxa during the Eocene and for temperate plants until the Late Miocene (e.g., [50,51,52,53,54,55,56,57,58] and references therein).

The Late Eocene Dodori MT pollen from Kenya shares morphological features with both fossil European pollen of the American-African clade and, therefore, extant *Piranhea*, as well as extant *Hyaenanche* pollen (Figure 6 and Table 1, Table 2, Table 3 and Table 4). Based on our comparison, the Dodori MT pollen type is morphologically close to both *Hyaenanche* and *Piranhea*. Notably, the shared features with *Piranhea* and the Eocene European fossils support an Early to Middle Eocene dispersal into Africa from an ancestral European stock. Even though this is the first and only Paleogene Picrodendraceae record from Africa, we hypothesize that prior to the end-Eocene, Picrodendraceae had already dispersed across the African continent. This will hopefully be verified by future studies on African paleopalynofloras using the single grain method and combined LM and SEM. Fossil Miocene *Hyaenanche* pollen from South Africa shows that this genus had already dispersed into its current endemic region prior to the Early Miocene [13]. How and in what way this relict distribution evolved until the present can only be resolved by investigating further Eocene to Holocene palynofloras across Africa using the method applied herein.

### 3.5. The Paleoecology of Eocene Hyaenanche Based on Fossil and Modern Counterparts

Today, *Hyaenanche* is restricted to hot steppe (BSh) or cool steppe (BSk) climates but also occurs under hot desert (BWh) and warm temperate summer dry (Csb) climates. Based on this, it could be assumed that during the earliest Late Eocene (Early Priabonian), the vegetation in the coastal area of Dodori, Kenya, endured continuous dry climate conditions. However, the paleopalynological assemblage, associated with the *Hyaenanche* Dodori MT pollen, comprises countless other spore and pollen types from plants with potential modern analogs presently growing under various humid subtropical to tropical climates. These include between 15 and 20 different palm pollen types, among them both *Sclerosperma* and *Nypa* [18]. *Sclerosperma* is a small understory palm, presently occurring in lowland rainforests and swampy coastal areas of tropical Western and Middle Africa [59]. *Nypa* is a typical mangrove element growing under tropical conditions in coastal areas of Southeastern Asia. Palms, in general, are predominantly tropical, with their highest diversity and abundance in regions with a CMMT ≥ 18 °C and MART ≤ 10 °C [60]. Therefore, relying solely on the diverse palm pollen types discovered in the same sample as the *Hyaenanche* Dodori MT pollen already lays the ground for interpreting the early Late Eocene climate around the Dodori site, coastal Kenya, Eastern Africa, as humid tropical. Additional examples of fossil pollen types from this paleopalynoflora representing families/genera that are presently important components of subtropical to tropical ecosystems, especially humid forests, include *Bombax* (Malvaceae), Melastomataceae, Schisandraceae, and Sapotaceae. Based on the geological evidence from the Dodori-1 well, the stratigraphic interval where the sample was collected was interpreted as representing a delta-front environment [16]. The occurrence of interbedded sandstones and mudstones with lignite, in combination with freshwater (algae) and marine (dinoflagellate cysts, foraminifera) microfossils [16], as well the high diversity of spores and pollen from both terrestrial and aquatic plants (e.g., [18,23]), suggests complex and varied lowland coastal environments. These can be summarized from inland towards the sea as comprising well-drained lowlands to wetlands, delta plains with associated floodplains, freshwater lakes, swamps, and brackish lagoons. An extensive delta environment in this region of Eastern Africa during the Eocene has already been suggested by Couvreur ([61], (fig. 2b)). This intricate lowland paleo-delta environment is prone to have sustained varied vegetation units occurring mostly within Tropical and Subtropical Moist Broadleaf Forest and Mangrove paleo-Biomes. Humid tropical vegetation during the Eocene of Eastern Africa would be in correspondence with previous hypotheses on the latitudinal position of the continent and general topography at that time. During the early Paleogene, the broadest part of Africa was located between latitudes 0° and 15° N (and Kenya at c. 10° S) and the physical setting of the continent was characterized by “isolation” from other landmasses and a general lack of mountainous regions. Without obstructions to moist air masses, this allowed the development of vast vegetative lowlands across the continent, potentially resulting in the development of a tropical pan-African rainforest (e.g., [61,62,63]). The assumption of a pan-African rainforest prevailing throughout the Paleogene has so far relied on Paleocene and/or Eocene Western and Middle African plant fossil records, encompassing rich and diverse spore and pollen assemblages investigated with LM only (e.g., [64,65,66,67,68]). Until now, the lack of Eocene plant fossils from Eastern Africa suggestive of rainforest environments has discredited the theory of a Paleogene pan-African rainforest (e.g., [69]). In addition, Eastern African Eocene paleofloras from Tanzania, positioned south of the Dodori site, indicate woodland (that endured dry climate with pronounced seasonality) rather than rainforest [70,71,72,73]. On the contrary, in southeastern North Africa, north of the Dodori area, Eisawi and Schrank [74] reported warm and humid climate conditions for the spore and pollen flora encountered from the Eocene of southeast Sudan. Based on the paleobotanical data at hand, it is hard to conclude about the extent of tropical rainforests in the Eocene of Africa, if a pan-African rainforest existed at that time, and if present, how far north or south it extended. More evidence from fossil Eocene floras across the continent is needed to resolve this. In any case, the paleopalynoflora associated with the *Hyaenanche* Dodori MT pollen is suggestive of tropical lowland wetland rainforests in the coastal area of Kenya during the early Late Eocene. Such vegetation and climate also correspond to the environments visualized as nursing the ancestral *Piraneha*/*Hyaenanche* lineage that dispersed into Africa from Europe. During the Eocene, when the *Piranhea*/*Hyaenanche* lineage had dispersed from the Americas across the NALB, Europe endured a hot and humid paratropical climate comparable to that of present-day moist tropics and subtropics [75,76,77]. This paleoclimate regime sustained a unique thermophilic flora, the so-called Paratropical Rainforest [78]. The most renowned fossil assemblage from that time in Europe is from Messel, Germany, known for its uniquely preserved amphibians, reptiles, fishes, birds, mammals, insects, and plants, reflecting paratropical rainforest environments (e.g., [79,80,81,82,83,84,85]). Another example is the Early Eocene, Ypresian, Krappfeld flora, Austria, which also comprises fossil *Picrodendraceae* pollen resembling *Piranhea* (Table 1, Table 2, Table 3 and Table 4). The Krappfeld paleopalynoflora has been studied with combined LM and SEM by [14,86,87,88] and is both rich and diverse in woody (trees, shrubs, lianas, etc.) as well as herbaceous (aquatic) plants, especially angiosperms typical of present-day humid warm temperate to tropical climates. The palynoflora comprises, among others, at least fourteen different Arecaceae pollen types (incl. Arecoideae, Calamoideae, and Nypoideae), as well as pollen of Araceae (*Gonatopus*), Chloranthaceae, Cyrillaceae, Icacinaceae (*Iodes*), Malvaceae (*Bombax*), Moraceae, Nyssaceae (*Mastixia*), Pandanaceae (*Pandanus*), Restionaceae, and Stemonuraceae (*Gomphandra*). The complete palynoflora reflects varied vegetation units along the Early Eocene coastline of the Krappfeld region ([14], text-fig. 3), a comparable scenario to that of the early Late Eocene of Dodori, Kenya, Eastern Africa.

When all geological and paleontological data/facts from the early Late Eocene of Dodori, Kenya, as well as those from the Eocene of Europe, are considered, the present-day habitat and climate to which *Hyaenanche* plants are confined seem out of place for their Eocene predecessors. To estimate the paleoecological aspects of Eocene *Hyaenanche*, the paleoecology of closely related fossil relatives in combination with ecological parameters of *Tetracoccus*, *Piranhea*, and *Picrodendron* (the predecessors of *Hyaenanche* within the American-Afro-Indian clade) are likely to provide a more holistic picture of the climatic and habitat range occupied by *Hyaenanche* in the Eocene of Africa and throughout the Cenozoic. When all three genera of the ancestral lineage of *Hyaenanche* are considered, *Tetracoccus*, *Picrodendron*, and *Piranhea*, it becomes clear that plants of this clade can tolerate both prolonged droughts (dry seasons) as well as a high amount of precipitation equally distributed over the year (Figure 4; Supplementary Materials File S2). What really defines these taxa and delimits their distribution is the mean temperature of the coldest month (MTCM). Plants from all four genera are more or less confined to areas with equatorial/tropical (A) climates and/or arid (B) climates, where the MTCM is ≥ 18 °C, except for cold arid desert (BWk) climates. *Picrodendron* and *Piranhea*, who form a sister clade to the Afro-Indian clade comprising *Hyaenanche* (as the earliest diverging lineage), both thrive in tropical rainforest (Af), tropical monsoonal (Am), and tropical winter-dry Savanna (Aw) climates. Compared to the Eocene climate of Europe, these extant climate categories seem likely to reflect the climate endurance of parent plants of the fossil *Piranhea*/*Hyaenanche* pollen encountered in Eocene sediments of both Europe and Africa.

Summarizing, we suggest that African Eocene *Hyaenanche* had a wider ecological and climatic range than is indicated by its single extant relative, *H. globosa*. During the Eocene, *Hyaenanche* probably grew under climates ranging from fully humid tropical rainforest (Af) to tropical winter dry (Aw). Based on the habitats of extant genera and the sedimentary context of the fossils, we also believe that the parent plants producing the *Hyaenanche* Dodori MT pollen were part of lowland vegetation units. In the lowlands, they could have been part of the landward margins of mangroves, floodplains and seasonally inundated forests, well-drained forests, or mixed coastal forests. The precipitation could have been evenly distributed over the year, or the plants endured winter dry seasonality. Neither would have been a delimiting factor.

## 4. Conclusions

Our study demonstrates that the origin and evolution of *Hyaenanche*, a relic plant now endemic to a small region of the Cape Province, South Africa, can be traced using a unique type of pollen from the fossil record. We discovered *Hyaenanche*-type pollen from early Late Eocene sediments, close to Dodori, Kenya, Eastern Africa. This fossil pollen is comparable to Early and Middle Eocene pollen from Europe, suggesting that this lineage dispersed from Europe into Africa prior to the Late Eocene. The European fossil pollen suggest they originate from an older Paleocene or Early Eocene North American stock that dispersed across the North Atlantic Land Bridge during the Early Eocene. This earliest African fossil record of *Hyaenanche*, far away from its present-day distribution, suggests the genus had a much wider distribution within Africa in the geological past. How widespread it was can only be revealed from additional studies on Paleogene palynofloras from across the continent. The sedimentary context in combination with the spore and pollen assemblages of Eocene *Hyaenanche* (and alike) pollen suggest that the parent plants grew in/under habitats/vegetation/climate different from that of present day *Hyaenanche*. It appears that the climate tolerance and habitat range of pre-extant *Hyaenanche* were broader than at present day. During the Eocene in Kenya, these plants were likely part of lowland wetland vegetation, which may have included the landward margins of mangroves, seasonally inundated floodplain forests, or mixed coastal forests. It seems that the amount or distribution of precipitation throughout the year was not a limiting factor for Eocene *Hyaenanche*. Our study also highlights the importance of incorporating ecological data from closely related genera when estimating the paleoecological aspects of monotypic relict taxa.

## Figures and Tables

**Table 1:** Pollen morphology of extant Picrodendraceae related to *Hyaenanche*.

Taxon	*Tetracoccus fasciculatus*	*Picrodendron baccatum*	*Piranhea trifoliata, P. longepedunculat*	*Hyaenanche globosa*
**Living or fossil/age**	Extant (5 spp.)	Extant (1 sp.)	Extant (4 spp.)	Extant (1 sp.)
**Distribution or locality**	South-west USA to north Mexico, North America	West Indies, Caribbean, North America	Brazil to Mexico, South to Central America	South Africa, Africa
**P/E ratio; shape**	Isodiametric to slightly oblate; Spheroidal	Isodiametric to slightly oblate; Spheroidal	Isodiametric to slightly oblate; Spheroidal	Isodiametric to slightly oblate; Spheroidal
**Outline in polar view/equatorial view**	Circular/Circular	Circular/Circular	Circular/Circular	Circular/Circular
**Equatorial diameter including echini (LM/SEM)**	31–35/30–33	~34/~29	25–31/24–29	32–39/30–38
**Equatorial diameter excluding echini (LM/SEM)**	30–33/28–30	N.O./N.O.	24–29/21–26	29–35/28–34
**Polar axis including echini (LM/SEM)**	29–31/27–28	26–34/N.O.	24–28/21–26	30–35/29–34
**Polar axis excluding echini (LM/SEM)**	27–30/25–26	N.O./N.O.	22–25/20–23	29–33/26–32
**Aperture type and number**	Stephano(6)porate	Stephano(5–8)porate	Stephano(6–7)porate	Stephano(6–7)porate
**Aperture position**	Pori at regular intervals,	Pori at regular intervals,	Pori at regular intervals, positioned at the equator	Pori at regular intervals, positioned at the equator
	positioned at the equator	positioned at the equator		
**Aperture diameter (SEM)**	3.0–4.4	N.O.	3.3–4.4	3.0–5.5
**Exine thickness excluding echini (LM)**	2–2.5	N.O.	1.7–2.0	1.4–1.6
**Pollen wall (SEM)**	Tectate	Tectate	Tectate	Tectate
**Sculpture (LM/SEM)**	Echinate/Echinate; nanogemmate to granulate, perforate in areas between echini	Echinate/Echinate; nanogemmate, perforate in areas between echini	Echinate/Echinate; fossulate, perforate, and nanogemmate in areas between echini	Echinate/Echinate; fossulate, perforate, and nanogemmate in areas between echini
**Number of echini in central polar area (SEM)**	20–25 per 100 μm^2^	N.O.	12–20 per 100 μm^2^	15–30 per 100 μm^2^
**Echini height (SEM)**	1.1–2.2	3≥	1.6–2.7	1.0–2.0
**Aperture membrane (SEM)**	Nanogemmate to granulate	N.O.	Nanogemmate	Nanogemmate
**Notes and/or references**	Measurements and description extracted from Grímsson et al. ([13], (fig. 14))	Measurements and description extracted from Hayden et al. ([31], (figs. 3–6))	Measurements and description extracted from Grímsson et al. ([13], (figs. 11,12))	Measurements and description extracted from Grímsson et al. )[13], (fig. 6))

All measurements are given in µm; N.O. = not observed. Extant distribution of genera and fossil records are shown in Figure 6.

**Table 2:** Pollen morphology of fossil Picrodendraceae related to *Hyaenanche*.

Taxon	*Protoarecoidea buchananensis (1)*	*Echiperiporites* sp. (2)	*Malvacipollis tschudyi* (3)	*Echiperiporites tschudyi* (4)
**Living or fossil/age**	Fossil/Late Paleocene (Thanetian), 59.2–56.0 Ma	Fossil/Early, Middle, and Late Eocene (Ypresian to Priabonian), 56–33.9 Ma	Fossil/Middle and Late Eocene (Lutetian to Priabonian), 47.8–33.9 Ma	Fossil/Middle Eocene (Bartonian), 41.2–37.8 Ma
**Distribution or locality**	Buchanan, Tennessee, USA, North America	Clairborne and Jackson Groups, Mississippi, USA, North America	Saramaguacán Formation, Cuba, North America	Gosport Sand, Alabama, USA, North America
**P/E ratio; shape**	Isodiametric to slightly oblate; Spheroidal	Oblate; Spheroidal	Isodiametric to slightly oblate; Spheroidal	Oblate; Spheroidal
**Outline in polar view/equatorial view**	Circular to elliptic	Circular/Circular	Circular	Circular/Circular
**Equatorial diameter including echini (LM/SEM)**	N.O./23–27	32–34/N.O.	N.O./N.O.	28–39/N.O.
**Equatorial diameter excluding echini (LM/SEM)**	N.O./21–24	N.O./N.O.	30–35/N.O.	N.O./N.O.
**Polar axis including echini (LM/SEM)**	N.O./14–17	N.O./N.O.	N.O./N.O.	N.O./N.O.
**Polar axis excluding echini (LM/SEM)**	N.O./11–15	N.O./N.O.	N.O./N.O.	N.O./N.O.
**Aperture type and number**	Stephanoporate	Stephano(7)porate	Stephanoporate	Stephano(7)porate
**Aperture position**	Pori positioned at the equator	Pori positioned at the equator, additionally 2 pores on the hemisphere	Pori at regular intervals, positioned at the equator	Pori at regular intervals, positioned at the equator
**Aperture diameter (SEM)**	~3.4	N.O.	2 (LM)	N.O.
**Exine thickness excluding echini (LM)**	N.O.	N.O.	1–2	~ 1.5
**Pollen wall (SEM)**	Tectate	N.O.	N.O.	N.O.
**Sculpture (LM/SEM)**	N.O./Echinate; striate, perforate, and nanogemmate in areas between echini	Echinate/N.O.	Echinate/N.O.	Echinate/N.O.
**Number of echini in central polar area (SEM)**	18–20 per 100 μm^2^	N.O.	N.O.	N.O.
**Echini height (SEM)**	1.7–3.6	N.O.	N.O.	N.O.
**Aperture membrane (SEM)**	Nanogemmate	N.O.	N.O.	N.O.
**Notes and/or references**	Grímsson et al. [13] pointed out affinity to Picrodendraceae and not Arecaceae as described by Feldman ([32], (figs. 79–88)). Measurements and description based on micrographs provided in Feldman [32]	Measurements and description based on micrographs in Tschudy ([33], (pl. 3, figs. 13,14)). Grímsson et al. [13] pointed out affinity to *Picrodendron.* Age according to Ebersole et al. [34]	Measurements and description extracted from Graham et al. ([35], (figs. 73–75)). Age according to Batista Rodríguez [36]	Measurements and description based on micrographs in Frederiksen ([37], (pl. 2, figs. 21,22))

All measurements are given in µm; N.O. = not observed. Extant distribution of genera and fossil records are shown in Figure 6.

**Table 3:** Pollen morphology of fossil *Picrodendraceae* related to *Hyaenanche*.

Taxon	*Malvacipollis tschudyi* (5)	*Nothofagus* sp. (6)	*Compositoipollenites* sp. *(Aristogeitonia* type) (7)	Krappfeld MT (*Malvacipollis* sp.; *Iodes* sp.) (8)
**Living or fossil/age**	Fossil/Middle Eocene (Bartonian), 41.2–37.8 Ma	Fossil/Middle to Late Eocene (Bartonian to Priabonian); 41.2–33.9 Ma	Fossil/Early Eocene (Ypresian), 56–47.8 Ma	Fossil/Early Eocene (Ypresian), 56–47.8 Ma
**Distribution or locality**	North Twistwood Creek Clay Member, Alabama, USA, North America	Yazoo clay, Jackson Group, Mississippi, USA, North America	Salzburg, Austria, Europe	Krappfeld, Austria, Europe
**P/E ratio; shape**	Oblate; spheroidal	N.O./N.O.	Isodiametric to slightly oblate; spheroidal	Isodiametric to slightly oblate; spheroidal
**Outline in polar view/equatorial view**	Circular/Circular	Circular/N.O.	Circular/Circular	Circular/Circular
**Equatorial diameter including echini (LM/SEM)**	35/N.O.	N.O./N.O.	N.O./22–24	23–25/21–23
**Equatorial diameter excluding echini (LM/SEM)**	N.O./N.O.	N.O./N.O.	N.O./20–21	20–22/16–18
**Polar axis including echini (LM/SEM)**	N.O./N.O.	N.O./N.O.	N.O./N.O.	19–21/N.O.
**Polar axis excluding echini (LM/SEM)**	N.O./N.O.	N.O./N.O.	N.O./N.O.	15–17/N.O.
**Aperture type and number**	Stephano(7)porate	Stephano(5–7?)porate	porate	Stephano(7)porate
**Aperture position**	Pori at regular intervals, positioned at the equator	Pori at regular intervals, positioned at the equator	Pori positioned at the equator	Pori at regular intervals, positioned at the equator
**Aperture diameter (SEM)**	N.O.	N.O./N.O.	1.8–2	3.5–4.0
**Exine thickness excluding echini (LM)**	~1.5	N.O./N.O.	N.O.	1.3–1.5
**Pollen wall (SEM)**	N.O.	N.O./N.O.	N.O.	Tectate
**Sculpture (LM/SEM)**	Echinate/N.O.	Echinate/N.O.	Echinate/Echinate, perforate, and nanogemmate in areas between echini, striate at base of echini	Echinate/Echinate; fossulate, perforate, and nanogemmate in areas between echini
**Number of echini in central polar area (SEM)**	N.O.	N.O.	16–20 per 100 μm^2^	20–25 per 100 μm^2^
**Echini height (SEM)**	N.O.	N.O.	1.6–2.6	2.3–2.6
**Aperture membrane (SEM)**	N.O.	N.O.	Nanogemmate	Nanogemmate
**Notes and/or references**	Measurements and description based on micrographs in Frederiksen ([37], (pl. 2, figs 19,20)) and ([38], (pl. 8, fig. 27))	Measurements and description based on micrographs in Tschudy and Van Loenen ([39], (pl. 3, figs. 23,27,28)). Age according Ebersole et al. [34]	Measurements and description based on micrographs in Hofmann ([15], (pl. 5, figs. 10–12))	Measurements and description extracted from Hofmann and Zetter [14], (pl. 2, figs. 1–3), Hofmann et al. [40], (pl. 2, figs. J–L), and Grímsson et al. ([13], (fig. 16A,B,E,F))

All measurements are given in µm; N.O. = not observed. Extant distribution of genera and fossil records are shown in Figure 6.

**Table 4:** Pollen morphology of fossil *Picrodendraceae* related to *Hyaenanche*.

Taxon	Stolzenbach MT (9)	*Multiporopollenites* sp.; *Nothofagidites* spp. *(10)*	Profen MT (11)	*Hyaenanche* Dodori MT (12)	Saldanha MT (13)
**Living or fossil/age**	Fossil/Middle Eocene (Lutetian), 47.8–41.2 Ma	Fossil/Middle Eocene (Lutetian), 47.8–41.2 Ma	Fossil/Middle Eocene (Bartonian), 41.2–37.8 Ma	Fossil/Earliest Late Eocene, (Earliest Priabonian), ~37.71 Ma	Fossil/Early Miocene (Aquitanian to Burdigalian), 23.03–15.98 Ma
**Distribution or locality**	Stolzenbach, Germany, Europe	Wulfersdorfer Flözgruppe, Germany, Europe	Profen, Germany, Europe	Dodori, Kenya, Africa	Saldanha Bay, South Africa, Africa
**P/E ratio; shape**	Isodiametric to slightly oblate; Spheroidal	N.O.	Isodiametric to slightly oblate; Spheroidal	Isodiametric to slightly oblate; Spheroidal	Isodiametric to slightly oblate; Spheroidal
**Outline in polar view/equatorial view**	Circular/Circular	Circular/N.O.	Circular/Circular	Circular/Circular to elliptic	Circular/Circular
**Equatorial diameter including echini (LM/SEM)**	24–32/22–30	21–31/N.O.	28–30/26–30	23–33/21–30	28–38/26–35
**Equatorial diameter excluding echini (LM/SEM)**	22–30/20–28	N.O./N.O.	26–27/23–27	21–31 /19–28	25–35/23–32
**Polar axis including echini (LM/SEM)**	22–30/N.O.	N.O./N.O.	N.O./N.O.	20–21/18–19	27–30/25–27
**Polar axis excluding echini (LM/SEM)**	22–28/N.O.	N.O./N.O.	N.O./N.O.	19–20/17–18	25–28/22–24
**Aperture type and number**	Stephano(7)porate	Stephano(5–6)porate	Stephano(7)porate	Stephano(6–7)porate	Stephano(7)porate
**Aperture position**	Pori at regular intervals, positioned at the equator	Pori at regular intervals, positioned at the equator	Pori at regular intervals, positioned at the equator	Pori at regular intervals, positioned at the equator	Pori at regular intervals, positioned at the equator
**Aperture diameter (SEM)**	2.1–2.6	3–4 (LM)	1.0–2.3	1.7–3.2	3.0–4.5
**Exine thickness excluding echini (LM)**	1.2–1.4	~2.1	1.2–1.3	1.3–1.5	1.2–1.4
**Pollen wall (SEM)**	Tectate	N.O.	Tectate	N.O.	Tectate
**Sculpture (LM/SEM)**	Echinate/Micro-echinate to echinate; fossulate, perforate, and nanogemmate in areas between echini	Echinate/N.O.	Echinate/Echinate; fossulate, perforate, and nanogemmate in areas between echini	Echinate/Micro-echinate to echinate; fossulate, perforate, and nanogemmate in areas between echini, striate at base of echini	Echinate/Echinate; fossulate, perforate, and nanogemmate in areas between echini
**Number of echini in central polar area (SEM)**	19–30 per 100 μm^2^	N.O.	17–25 per 100 μm^2^	26–42 per 100 μm^2^	17–25 per 100 μm^2^
**Echini height (SEM)**	0.9–2.7	N.O.	1.7–3.1	0.6–1.7	1.0–2.5
**Aperture membrane (SEM)**	Nanogemmate	N.O.	Nanogemmate	Nanogemmate	Nanogemmate
**Notes and/or references**	Measurements and description extracted from Grímsson et al. ([13], figs. 16C–J, 17A–H))	Measurements and description based on micrographs in Lenz ([41], (pl. 8, figs. 19a–20b)) and Lenz ([42], (pl. 8, figs. 19a–20b))	Measurements and description extracted from Grímsson et al. ([13], (fig. 18A–H))	This study	Measurements and description extracted from Grímsson et al. ([13], (fig. 21A–H))

All measurements are given in µm; N.O. = not observed. Extant distribution of genera and fossil records are shown in Figure 6.

## Data Availability

All data produced during this study are either part of this published manuscript or provided within the Supplementary Materials.

## Supplementary Materials

The following supporting information can be downloaded at https://www.mdpi.com/article/10.3390/biology13121079/s1: File S1 and File S2.
