# Supplementary material for: Fossil Hyaenanche Pollen from the Eocene of Kenya: The Paleophytogeograpy and Paleoclimate of a Relict Plant Genus Endemic to the Cape Province, South Africa
Source: Biology (Basel). 2024 Dec 20;13(12):1079. doi: 10.3390/biology13121079 (PMC11672945; doi:10.3390/biology13121079)
Supplement: Supplementary file 1 [file biology-13-01079-s001.zip › Supplementary_Materials_File_02.pdf]

Supplementary Materials File 02:
Distribution, climate and biomes of *Hyaenanche, Picrodendron*, *Piranhea*, and *Tetracoccus*

1. Köppen-Geiger climate symbols and defining criteria (Peel et al. 2007, Kottek et al. 2006, Rubel et al. 2017, Cui et al. 2021).

1st	2nd	3rd	Description and criteria
A			**Equatorial**/tropical—MTCM ≥ 18 °C
	f		Rainforest, fully humid—P_{dry} ≥ 60 mm
	m		Monsoonal—not *Af* & P_{dry} ≥ 100 − MAP/25
	s		Savannah with dry summer—P_{sdry} < 60 mm
	w		Savannah with dry winter—P_{wdry} < 60 mm
B			**Arid**—MAP < 10 × $P_{threshold}$
	W		Desert—MAP < 5 × $P_{threshold}$
	S		Steppe—MAP ≥ 5 × $P_{threshold}$
		h	Hot arid—MAT ≥ 18 °C
		k	Cold arid—MAT < 18 °C
C			**Warm temperate** (subtropical to temperate)—T_{hot} > 10 °C & 0 °C < MTCM < 18 °C
D			**Snow** (cold-temperate to boreal)—T_{hot} > 10 °C & MTCM ≤ 0 °C
	s		Summer dry—P_{sdry} < 40 mm & P_{sdry} < P_{wwet}/3
	w		Winter dry—P_{wdry} < P_{swet}/10
	f		Fully humid, without a dry season, i.e. not *s* or *w*
		a	Hot summer—T_{hot} ≥ 22 °C
		b	Warm summer—not *a* & T_{mon10} ≥ 4
		c	Cool/cold and short summer—not *a/b* & 1 ≤ T_{mon10} < 4
		d	Extremely continental: cold/very short or no summer & very cold winter—not *a/b* & MTCM < −38 °C
E			**Polar—**T_{hot} < 10 °C
	T		Tundra—(T_{hot} > 0)
	F		Ice, permanent frost (‘frost desert’)—(T_{hot} ≤ 0)

MAP = mean annual precipitation, MAT = mean annual temperature, T_{hot} = mean temperature of the hottest month, T_{cold} = mean temperature of the coldest month, T_{mon10} = number of months where the temperature is above 10 °C, P_{dry} = precipitation of the driest month, P_{sdry} = precipitation of the driest month in summer, P_{wdry} = precipitation of the driest month in winter, P_{swet} = precipitation of the wettest month in summer, P_{wwet} = precipitation of the wettest month in winter, $P_{threshold}$ = varies according to the following rules: if 70% of MAP occurs in winter then $P_{threshold}$ = 2 x MAT, if 70% of MAP occurs in summer then $P_{threshold}$ = 2 x MAT + 28, otherwise $P_{threshold}$ = 2 x MAT + 14. Summer (winter) is defined as the warmer (cooler) six months period of October to March and April to September.

2. Color coding.

2.1. Köppen-Geiger climate map color coding.

Af	Am	As	Aw	BSh	BSk	BWh	BWk	Cfa	Cfb	Cfc
Csa	Csb	Csc	Cwa	Cwb	Cwc	Dfa	Dfb	Dfc	Dfd	Dsa
Dsb	Dsc	Dsd	Dwa	Dwb	Dwc	Dwd	EF	ET		

2.2. Biome map color coding using the Biome categories of Olson et al. (2001).

- Tropical & Subtropical Moist Broadleaf Forests
- Tropical & Subtropical Dry Broadleaf Forests
- Tropical & Subtropical Coniferous Forests
- Temperate Broadleaf & Mixed Forests
- Temperate Conifer Forests
- Boreal Forests / Taiga
- Tropical & Subtropical Grasslands, Savannahs & Shrublands
- Temperate Grasslands, Savannahs & Shrublands
- Flooded Grasslands & Savannahs
- Montane Grasslands & Shrublands
- Tundra
- Mediterranean Forests, Woodlands & Scrub
- Desert & Xeric Shrublands
- Mangroves

Abbreviations: Tropical & Subtropical Moist Broadleaf Forests (Tropic. & Subtropic. MBLF); Tropical & Subtropical Dry Broadleaf Forests (Tropic. & Subtropic. DBLF); Tropical & Subtropical Coniferous Forests (Tropic. & Subtropic. CF); Temperate Broadleaf & Mixed Forests (Temp. BLMF); Temperate Conifer Forests (Temp. CF); Tropical & Subtropical Grasslands, Savannahs & Shrublands (Tropic. & Subtropic. GLSSL); Temperate Grasslands, Savannahs & Shrublands (Temp. GLSSL); Flooded Grasslands & Savannahs (Flooded GLS); Montane Grasslands & Savannahs (Montane GLS), Mediterranean Forests, Woodlands & Scrub (MFWS); Deserts & Xeric Shrublands (DXSL).

3. Genus *Hyaenanche* Lamb. et Vahl, 1797

3.1. Species *Hyaenanche globosa* (Gaertn., 1790) Lamb. et Vahl, 1797

3.1.1. Köppen profile, distribution, and climate map – GBIF occurrences of *Hyaenanche globosa*; all specimens (herbarium and human observation) excluding duplicate occurrences (n = 65; preserved specimens, n = 22).

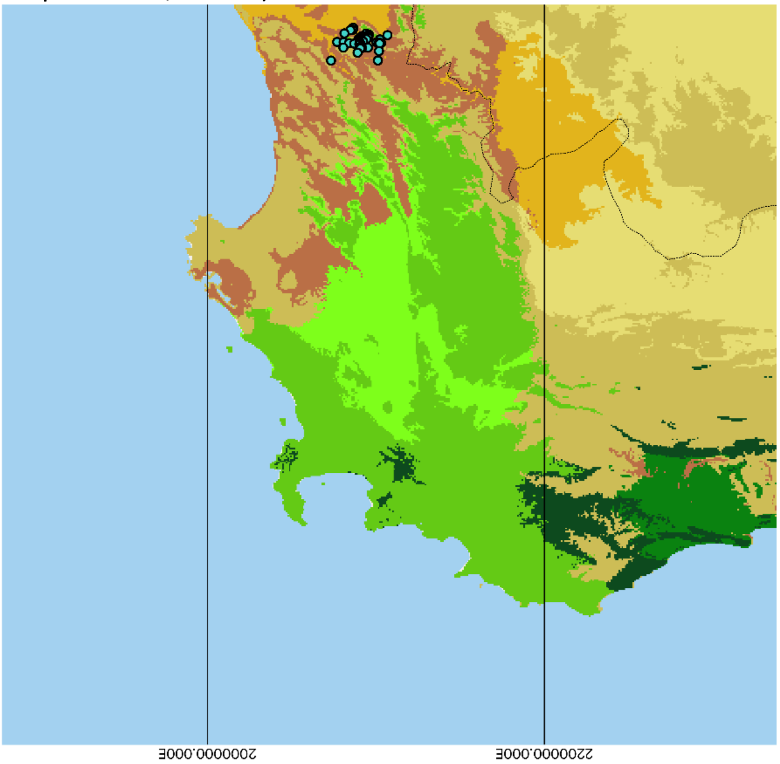

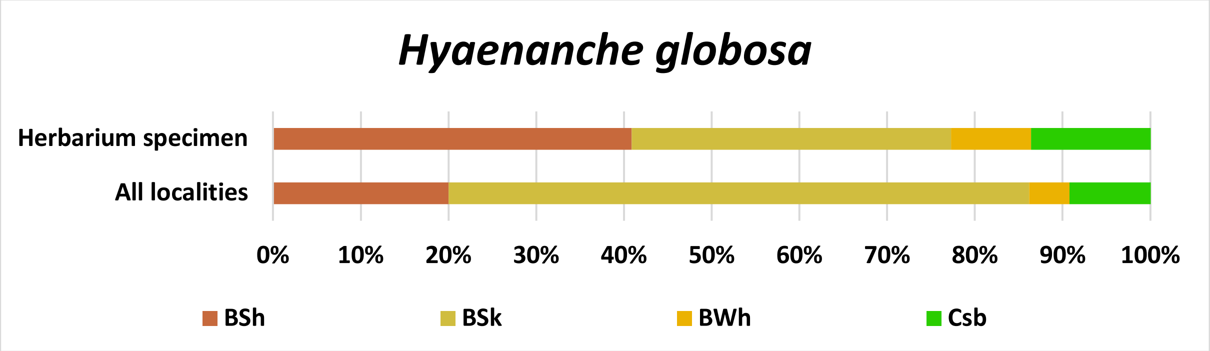

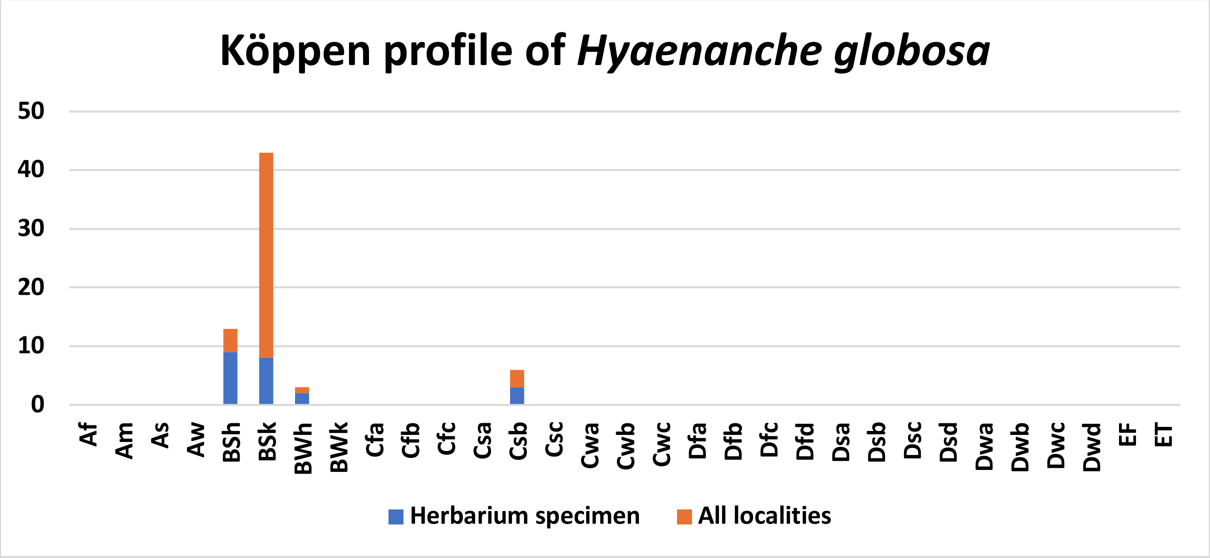

3.1.2. Biome profile, distribution, and climate map – GBIF occurrences of *Hyaenanche globosa*; all specimens (herbarium and human observation) excluding duplicate occurrences (n = 65; preserved specimens, n = 22).

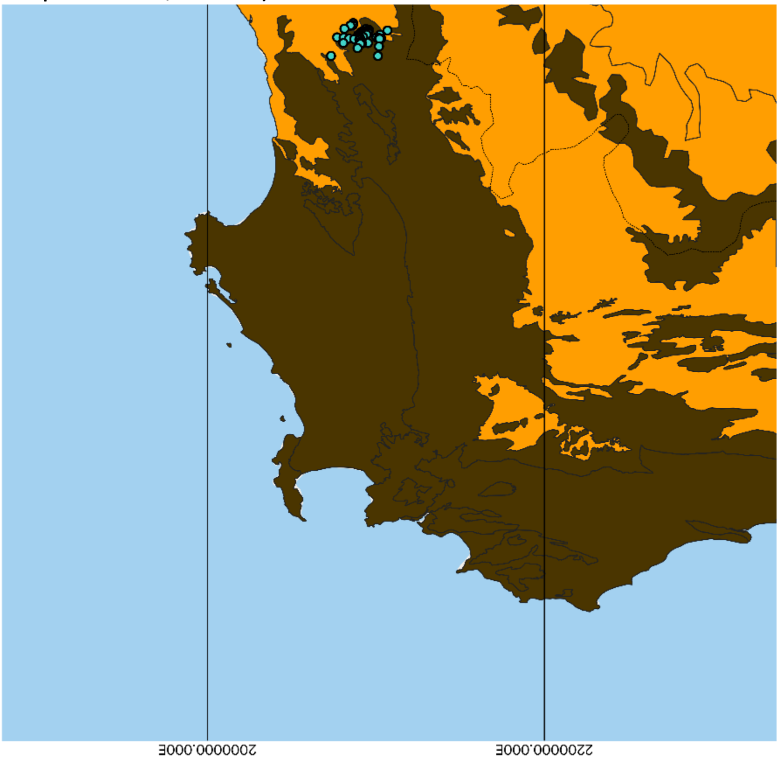

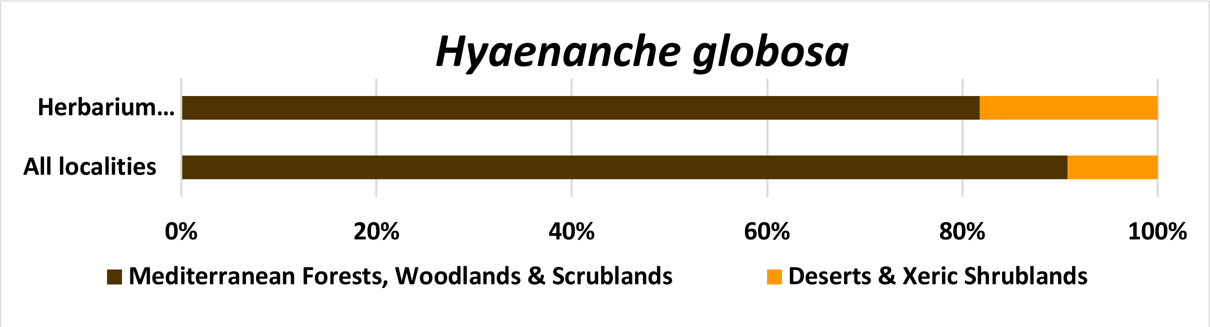

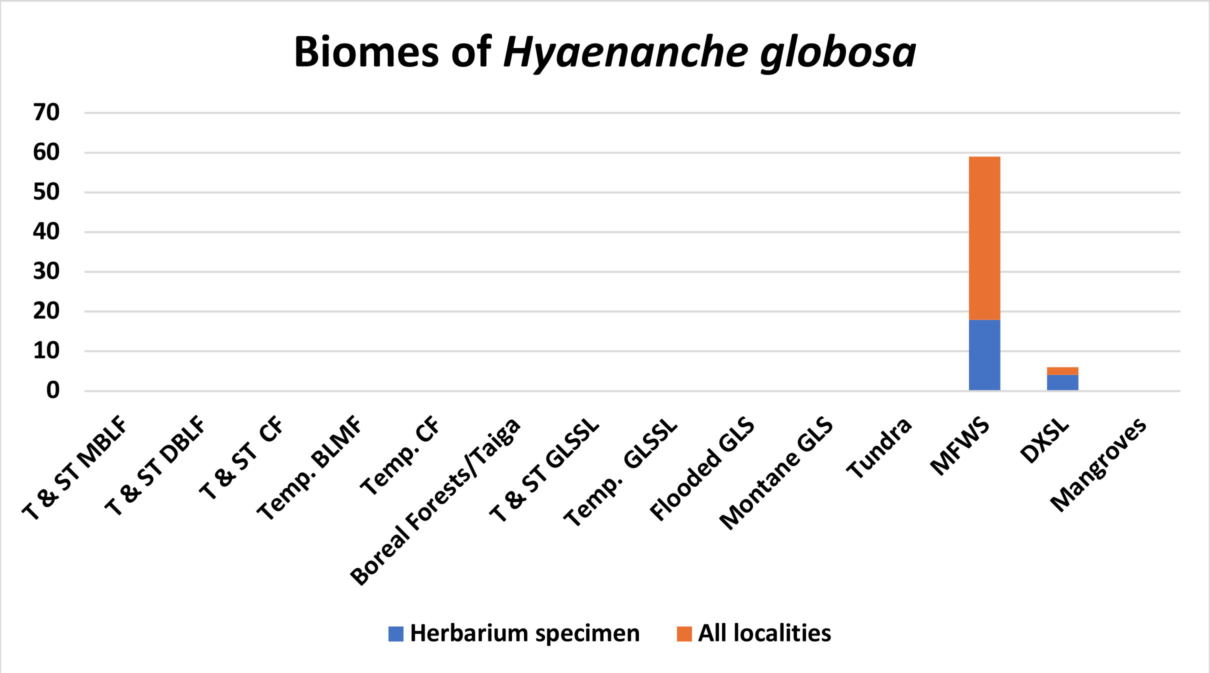

3.1.3. Climate graphs - based on 65 *Hyaenanche globosa* occurrences in GBIF

3.1.3.1. Mean monthly temperature (MMT) [°C]

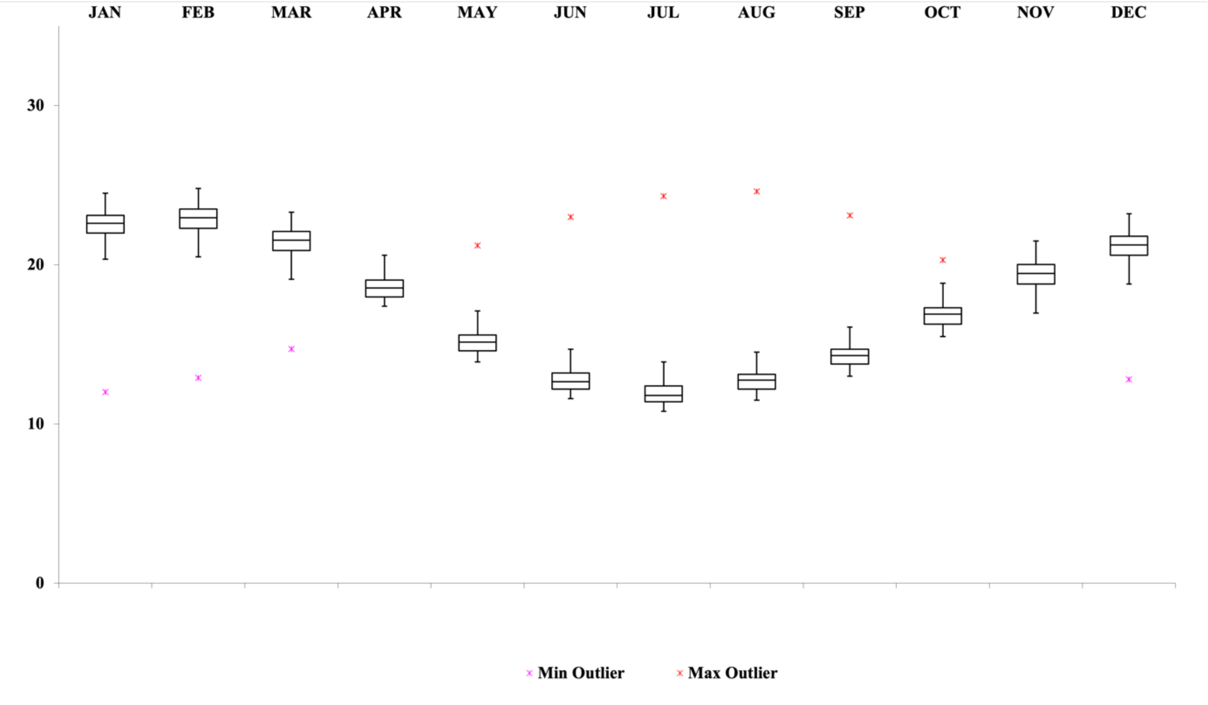

3.1.3.2. Maximum monthly temperature (MaxMT) [°C]

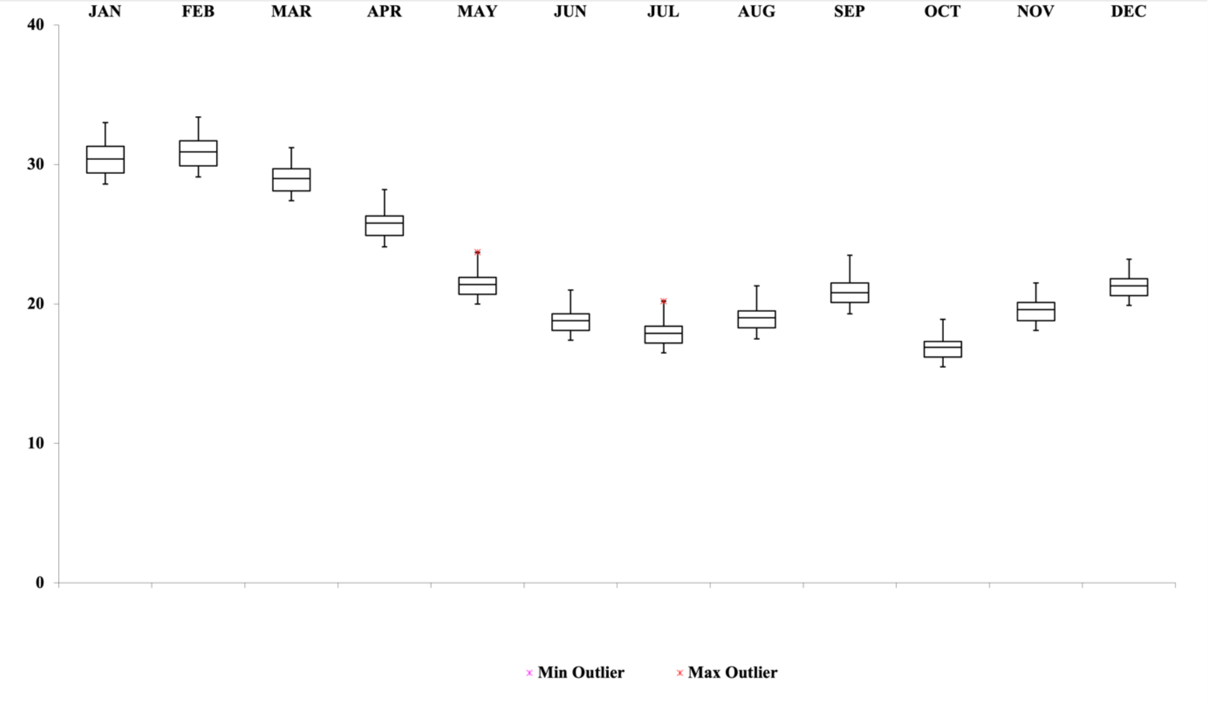

3.1.3.3. Minimum monthly temperature (MinMT) [°C]

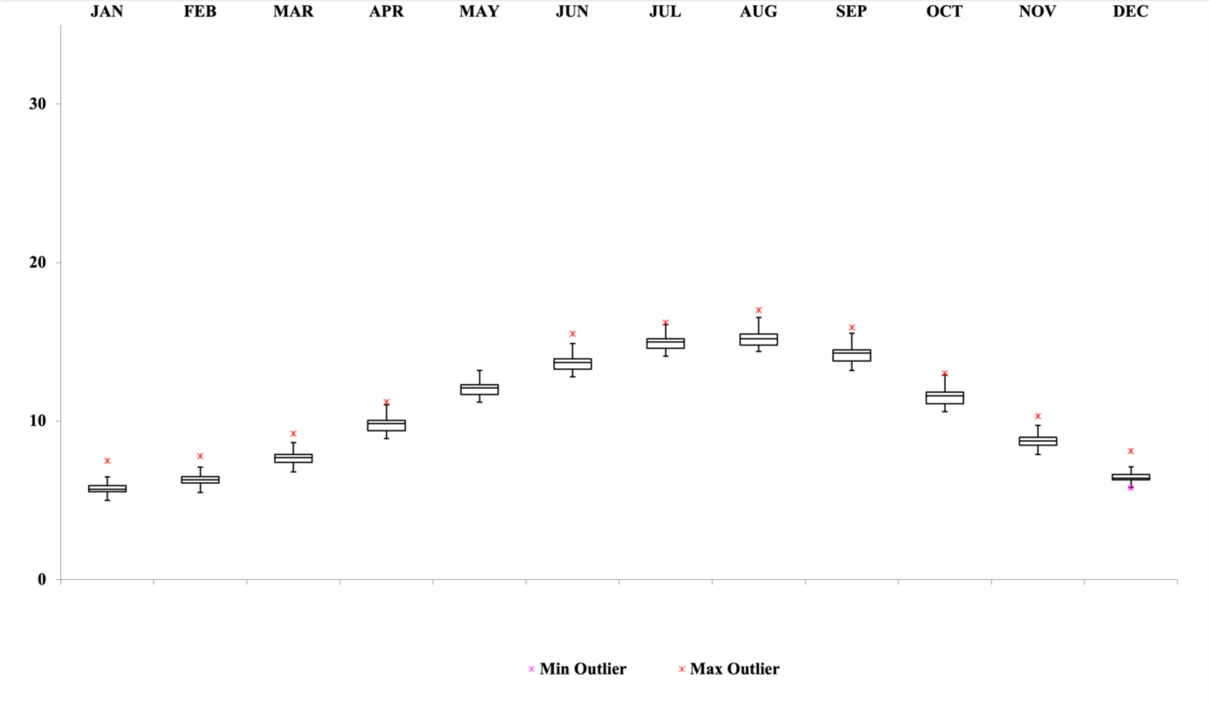

3.1.3.4. Mean monthly precipitation (MMP) [mm]

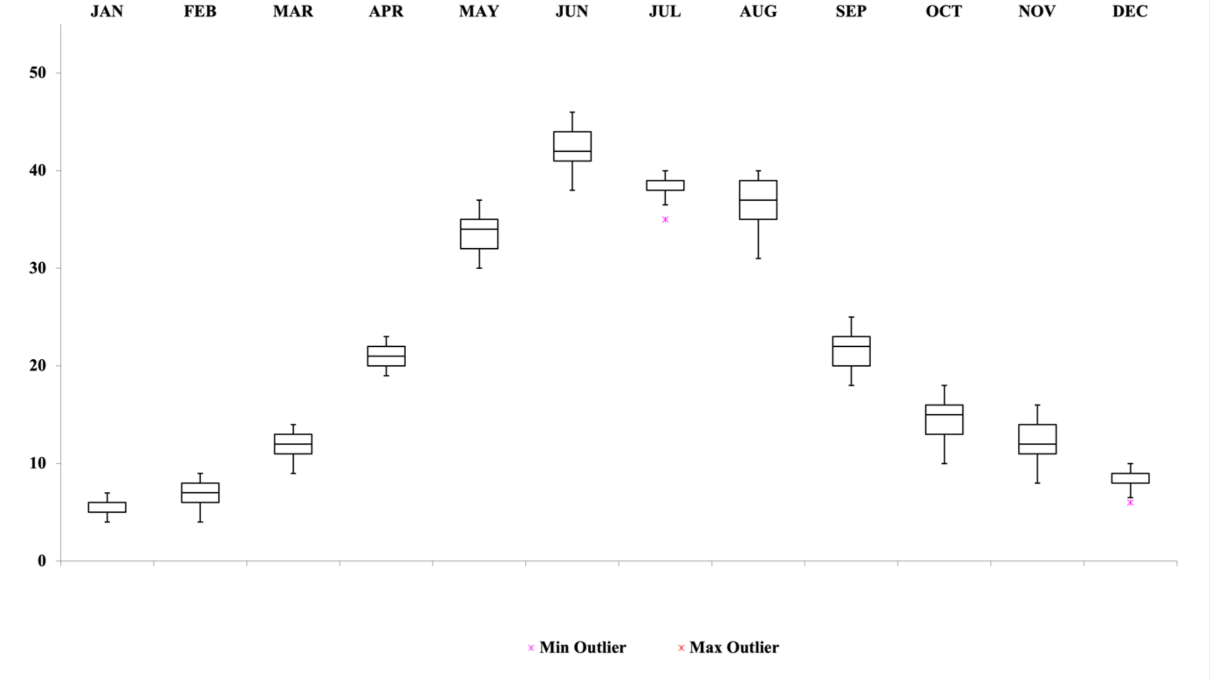

4. Genus *Picrodendron* Planch., 1846

4.1. Species *Picrodendron baccatum* (L., 1759) Krug et Urb., 1892

4.1.1. Köppen profile, distribution, and climate map – GBIF occurrences of *Picrodendron baccatum*; all specimens (herbarium and human observation) excluding duplicate occurrences (n = 75; preserved specimens, n = 21).

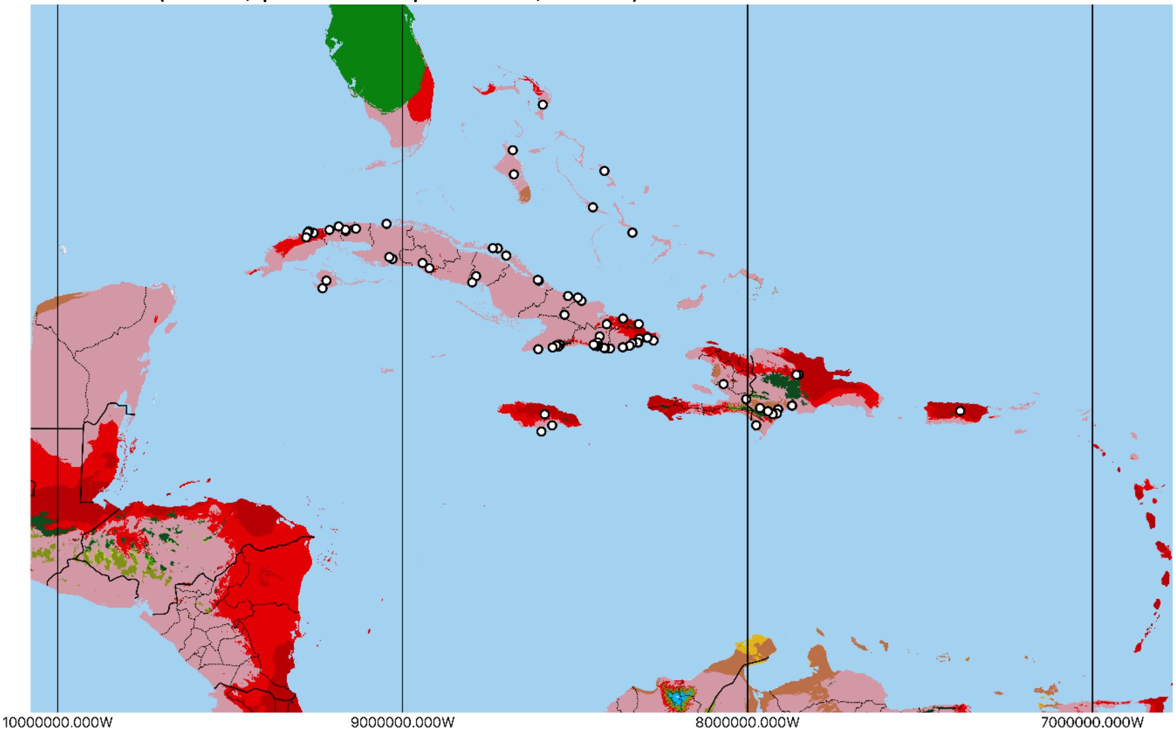

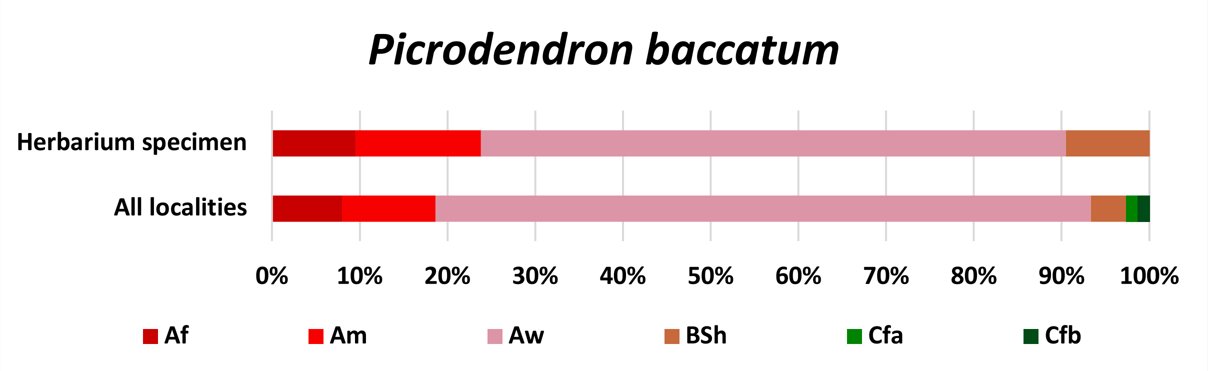

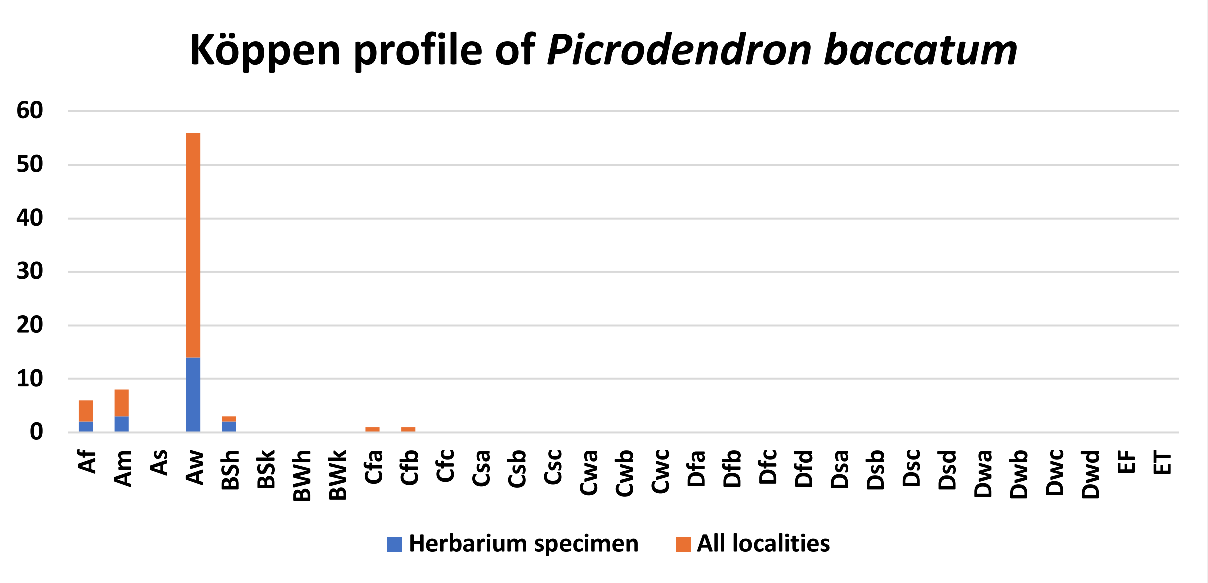

4.1.2. Biome profile, distribution, and climate map – GBIF occurrences of *Picrodendron baccatum*; all specimens (herbarium and human observation) excluding duplicate occurrences (n = 70; preserved specimens, n = 20).

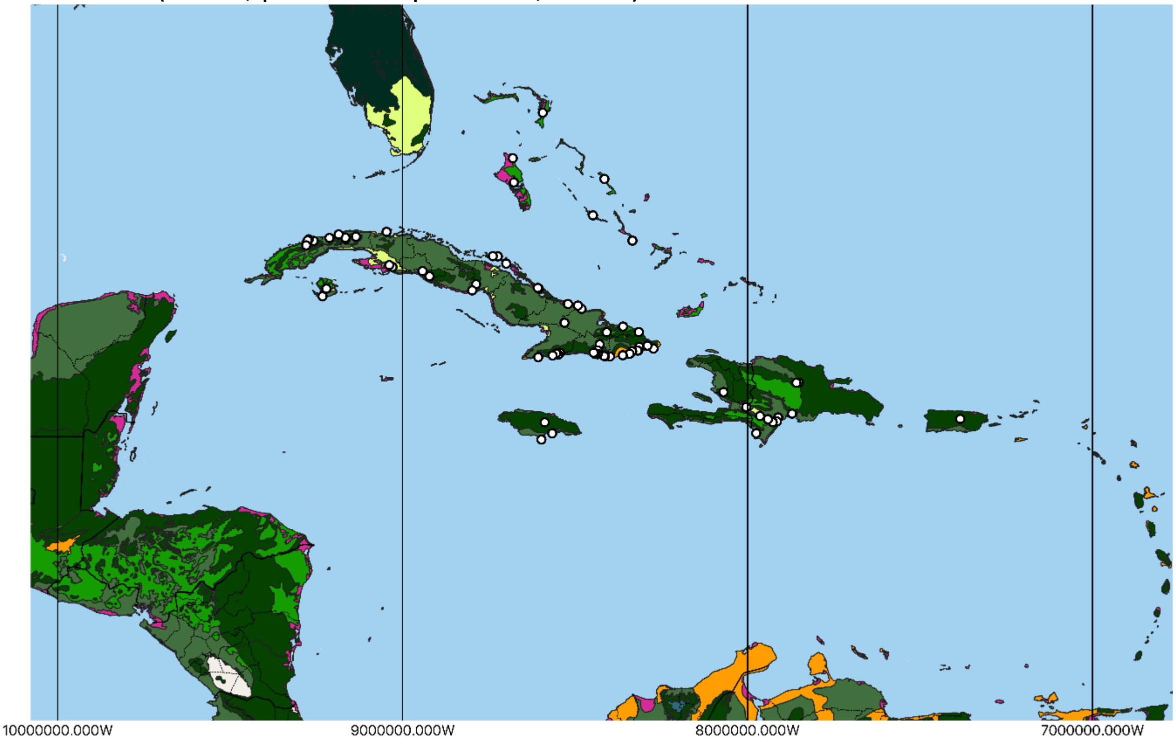

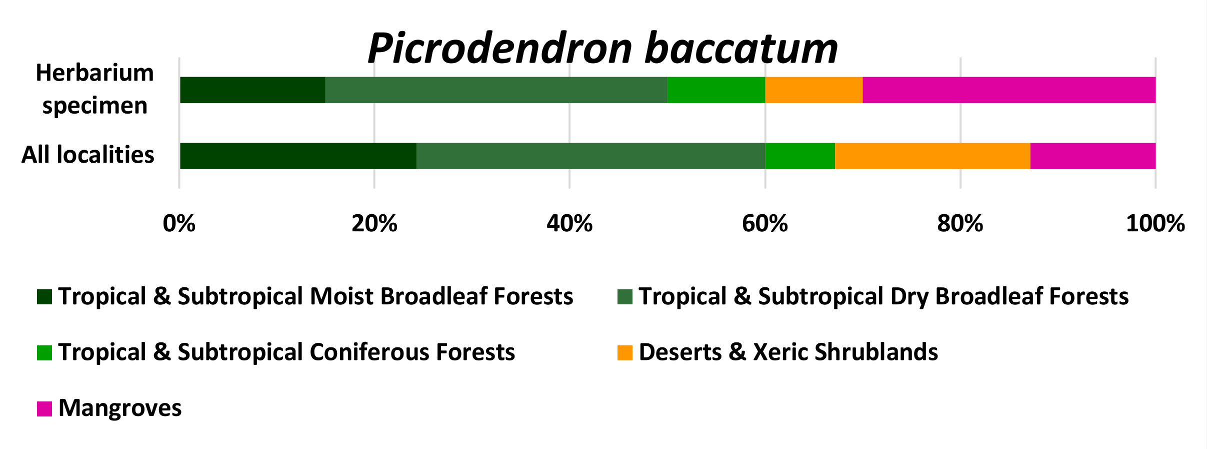

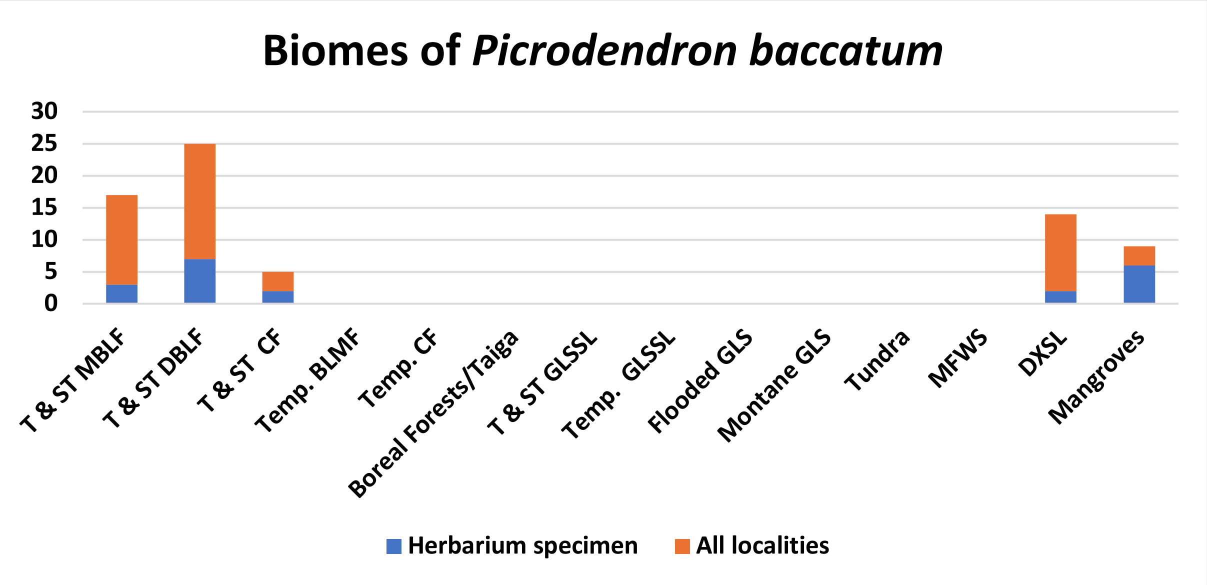

4.1.3. Climate graphs - based on 70 *Picrodendron baccatum* occurrences in GBIF

4.1.3.1. MMT [°C]

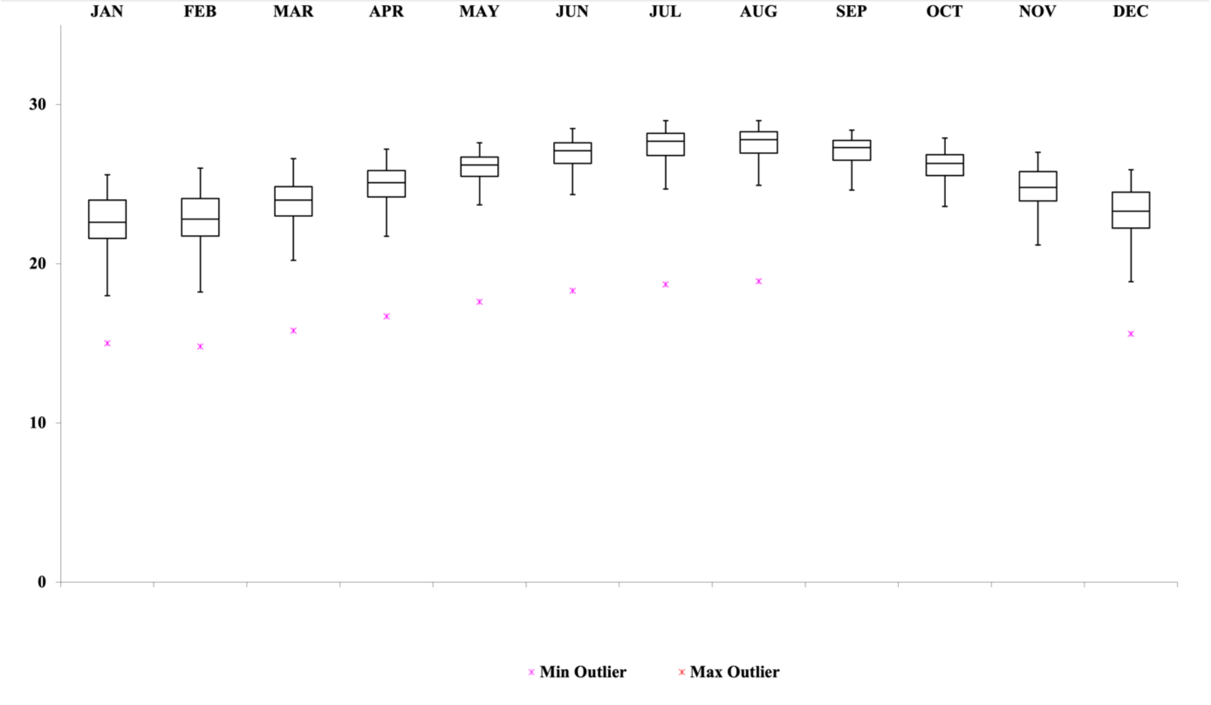

4.1.3.2. MaxMT [°C]

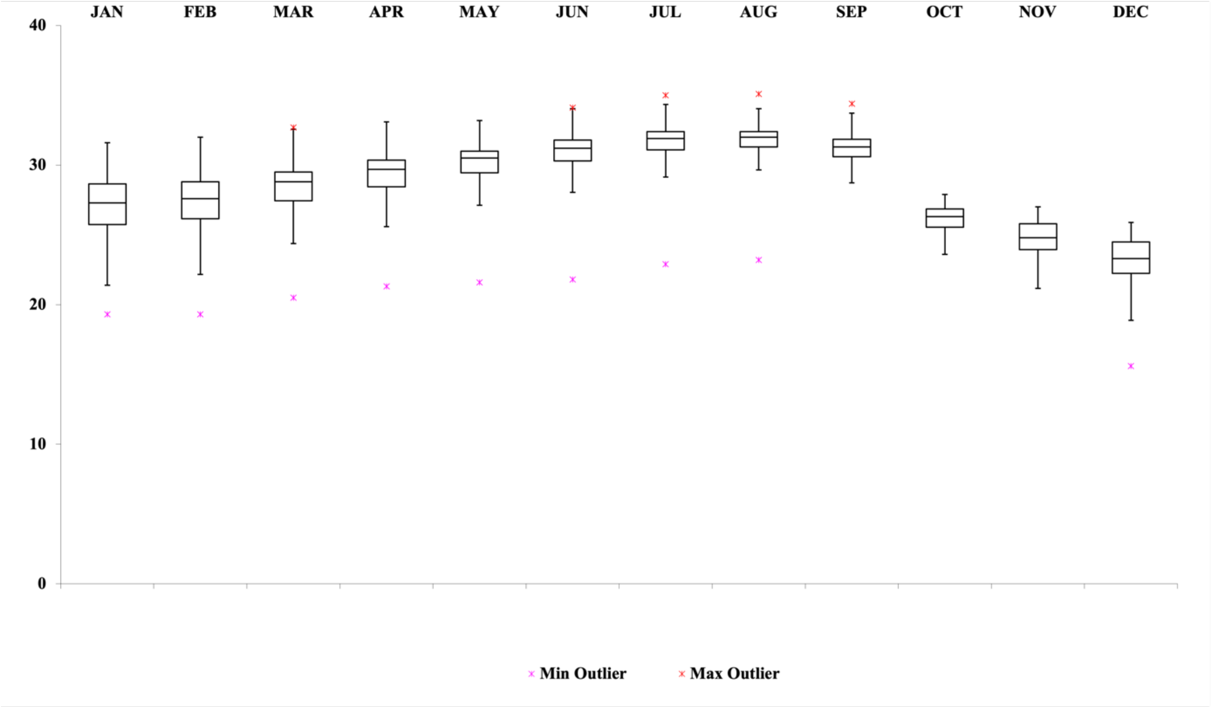

4.1.3.3. MinMT [°C]

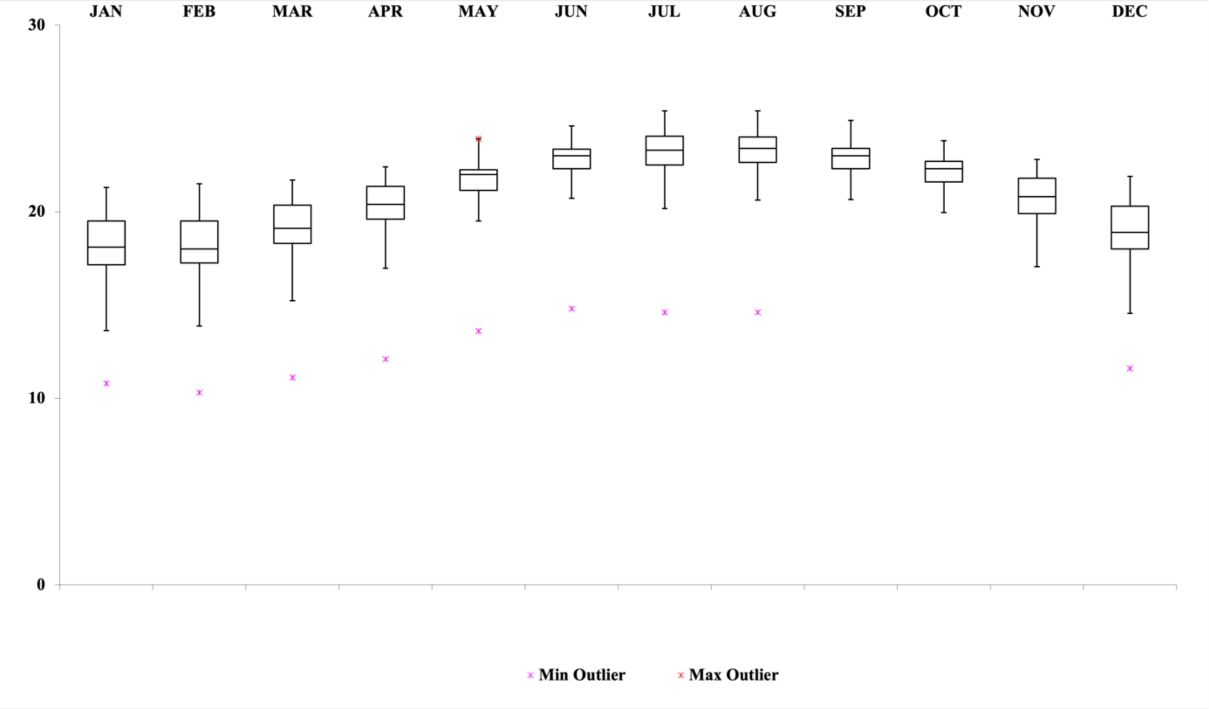

4.1.3.4. MMP [mm]

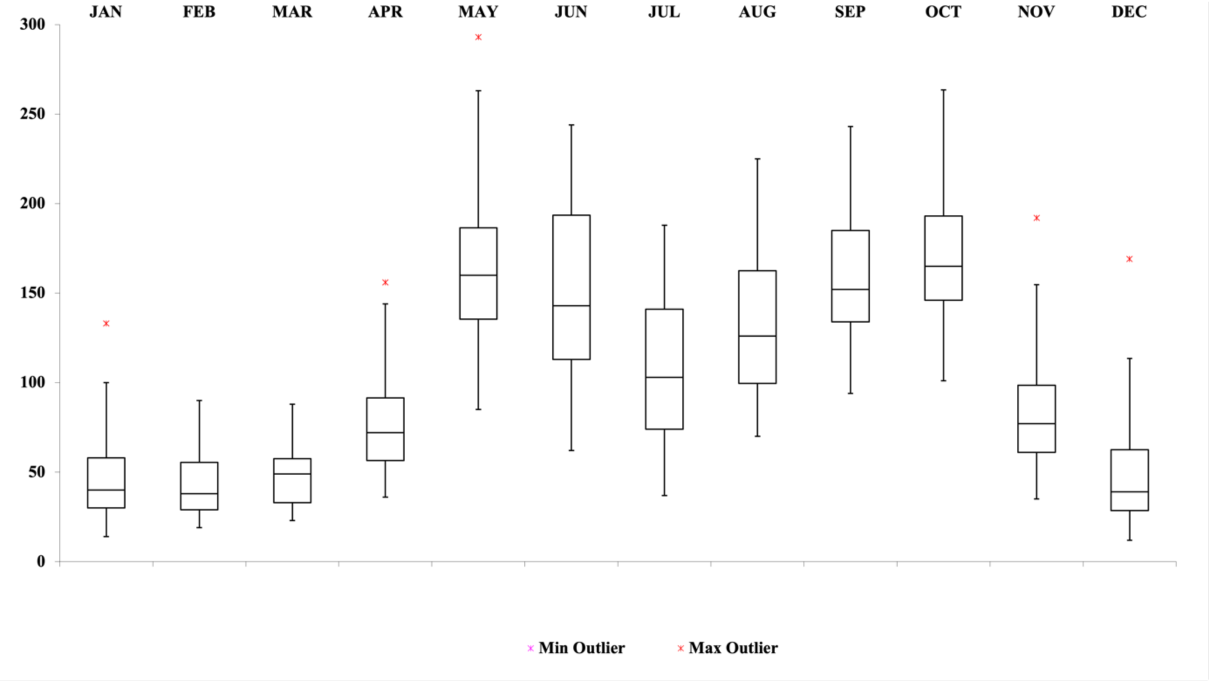

5. Genus *Piranhea* Baill., 1866

Genus distribution (Köppen-Geiger climates)

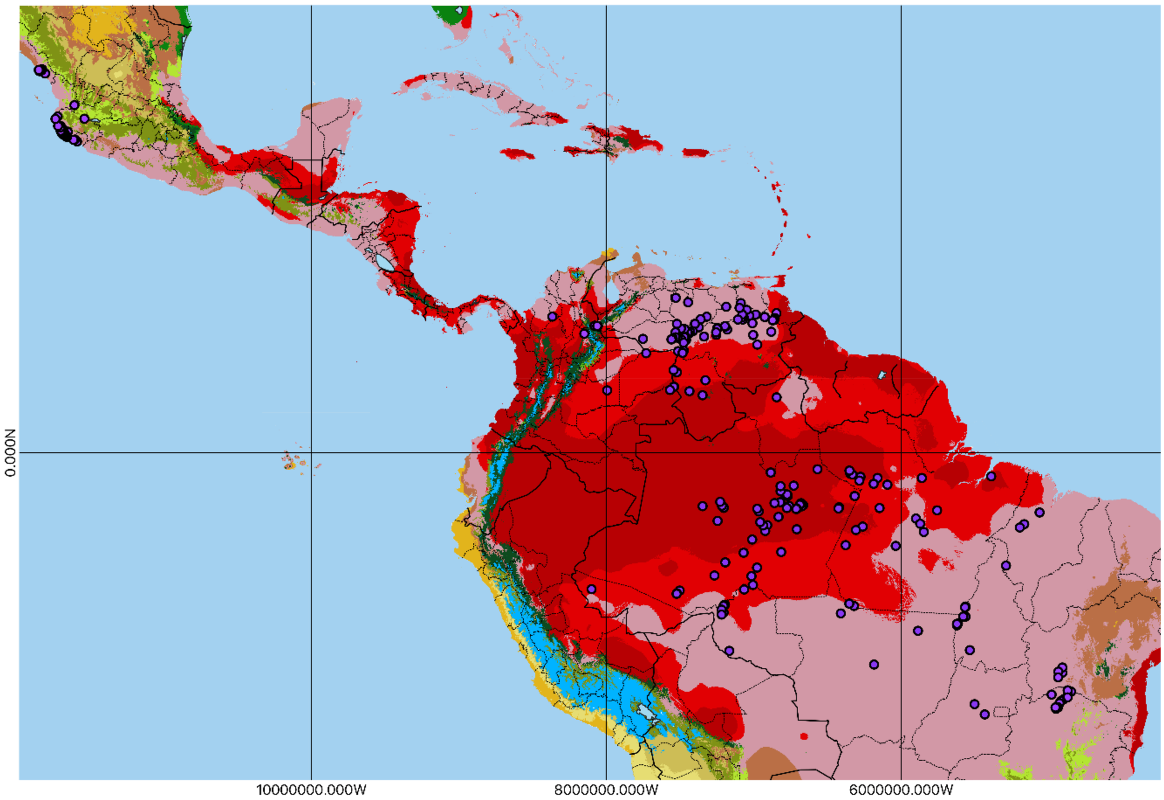

Genus distribution (Biomes)

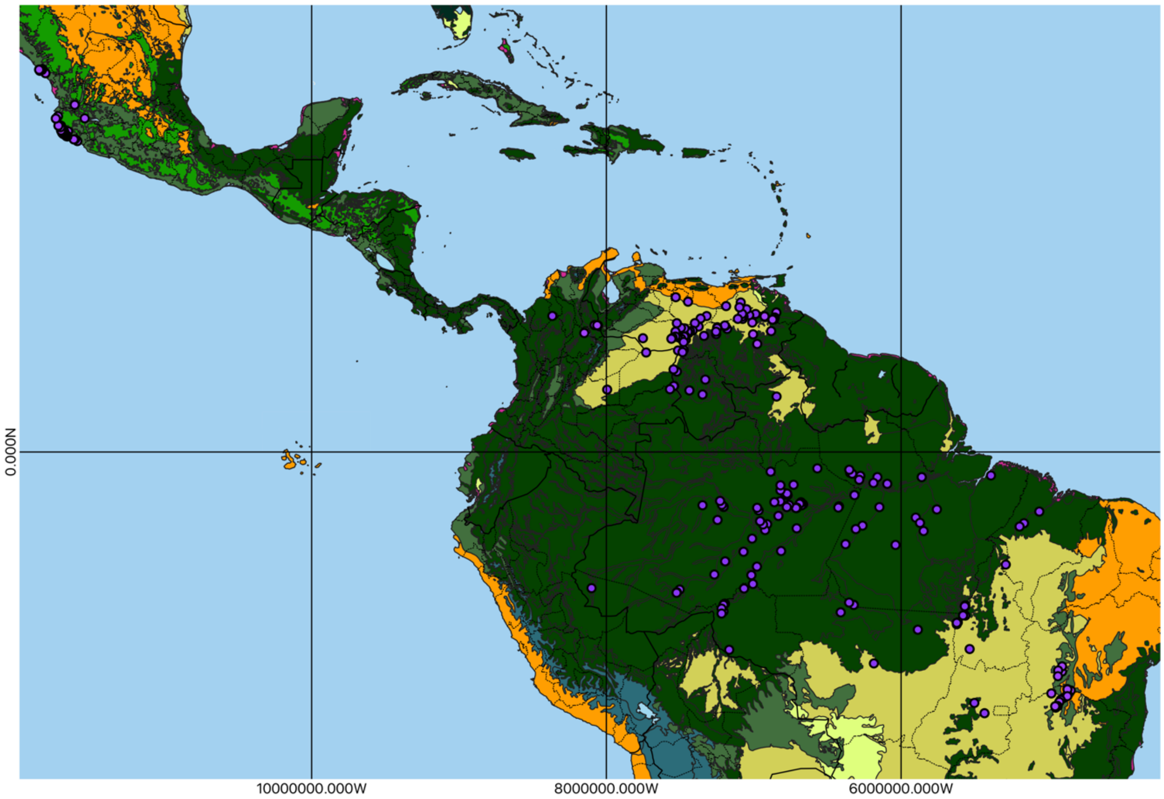

5.1. Species *Piranhea longepedunculata* Jabl., 1967

5.1.1. Köppen profile, distribution, and climate map – GBIF occurrences of *Piranhea longepedunculata*; herbarium specimens excluding duplicate occurrences (preserved specimens, n = 21).

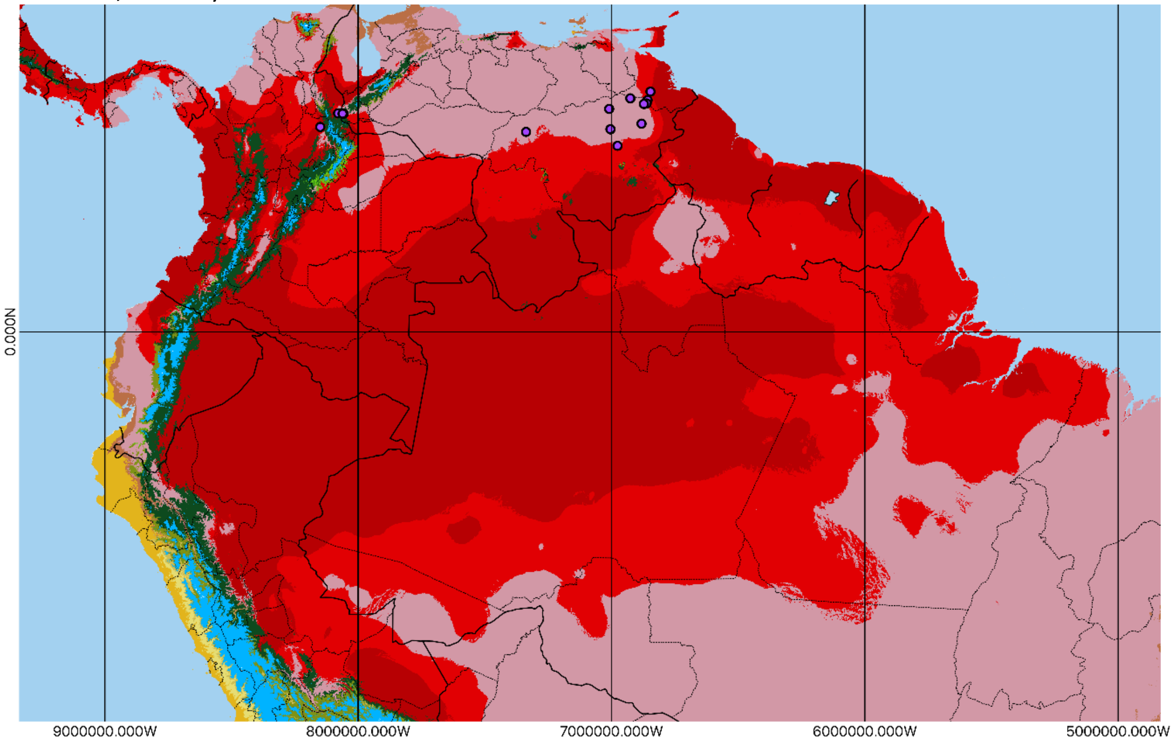

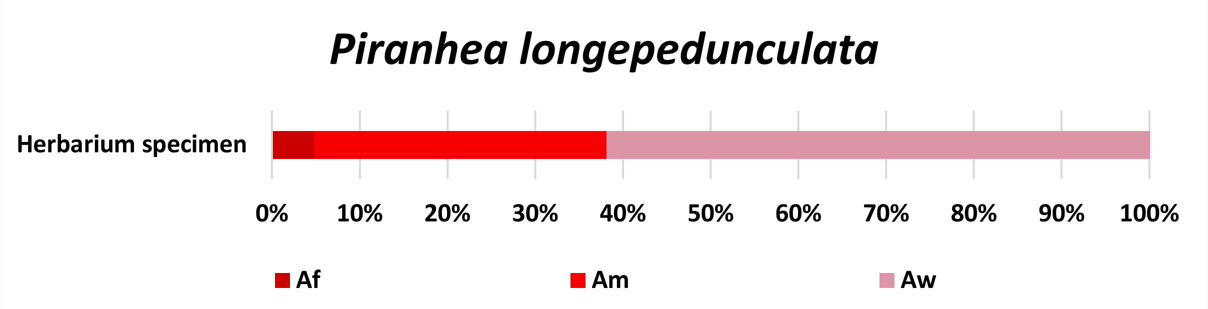

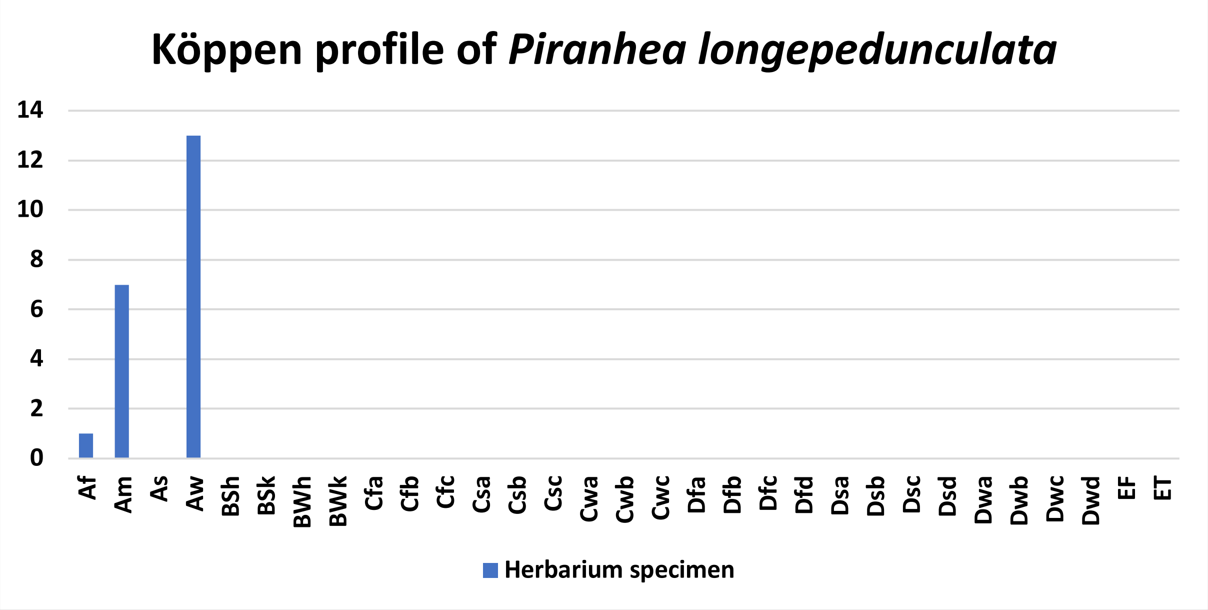

5.1.2. Biome profile, distribution, and climate map – GBIF occurrences of *Piranhea longepedunculata*; herbarium specimens excluding duplicate occurrences (preserved specimens, n = 21).

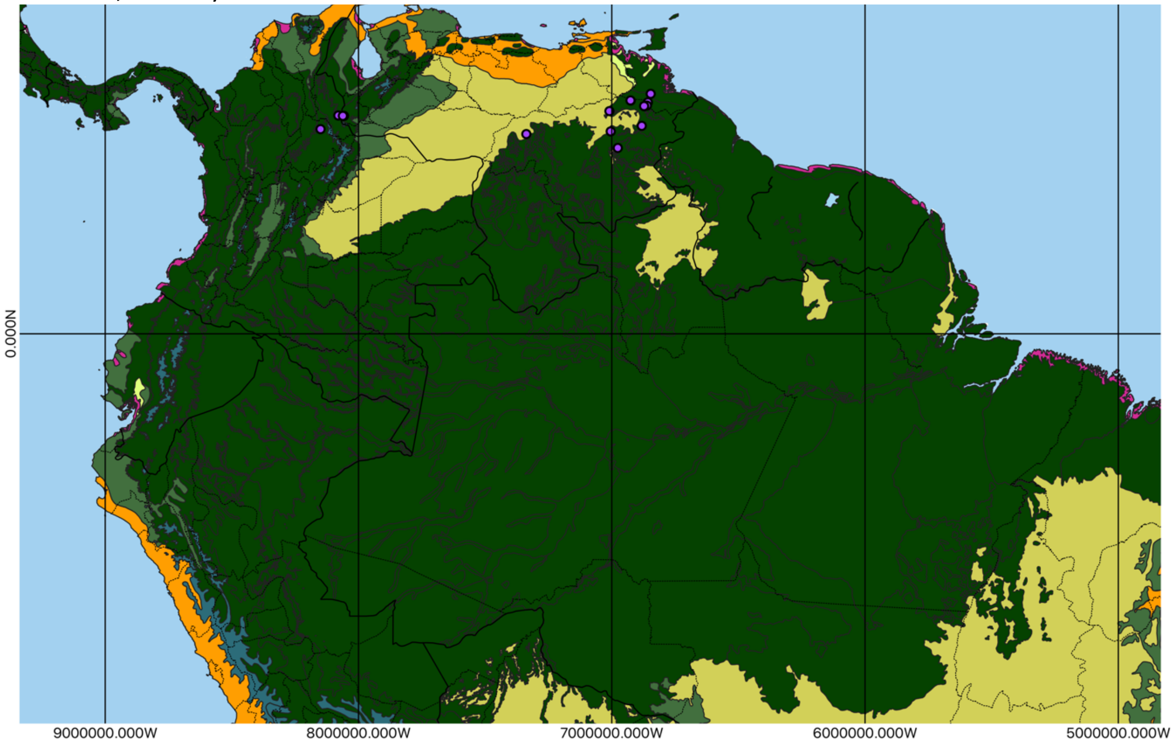

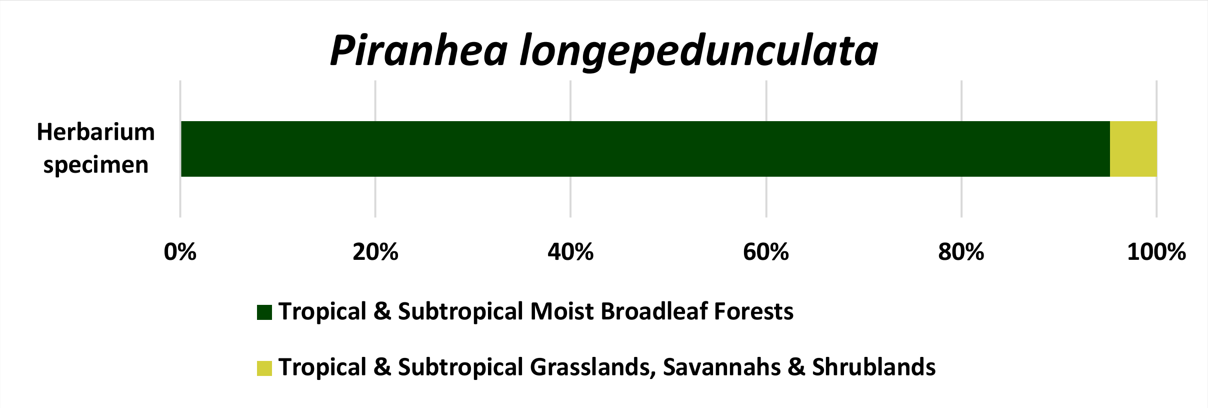

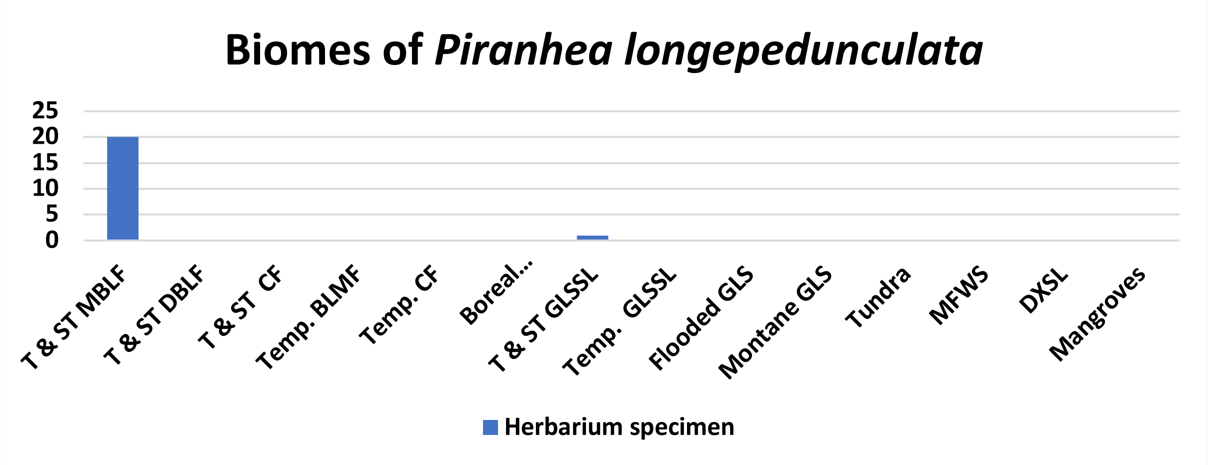

5.1.3. Climate graphs - based on 23 *Piranhea longepedunculata* occurrences in GBIF

5.1.3.1. MMT [°C]

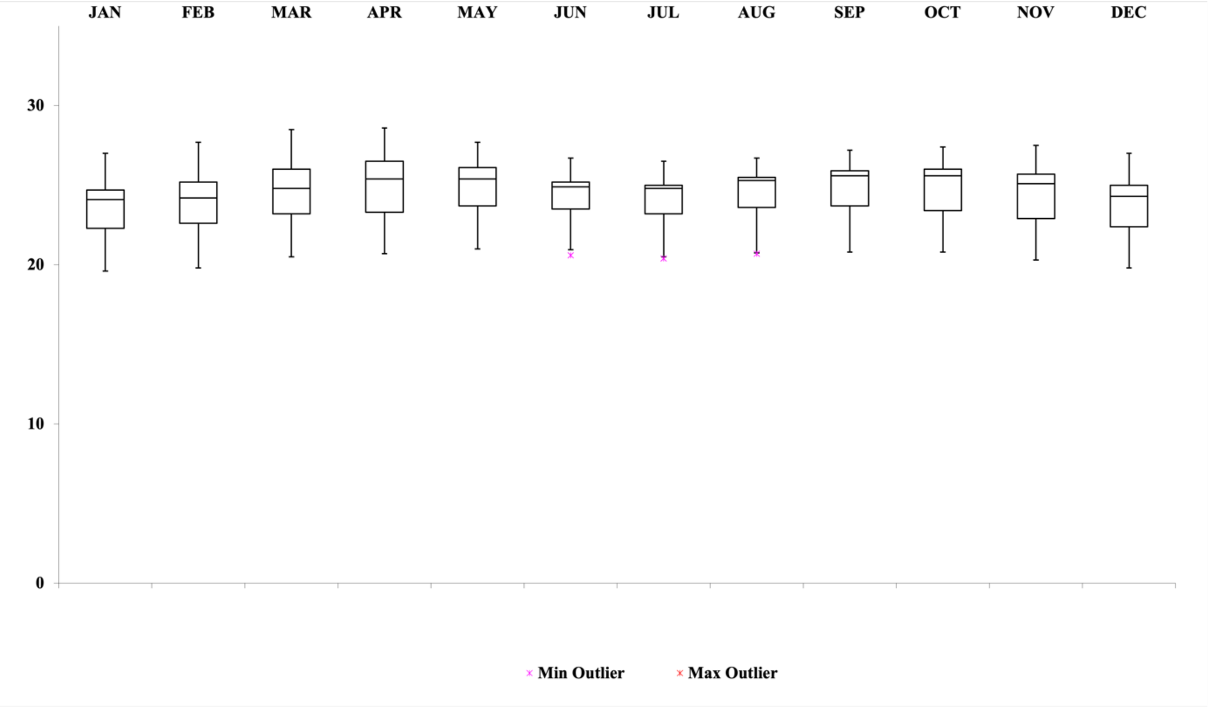

5.1.3.2. MaxMT [°C]

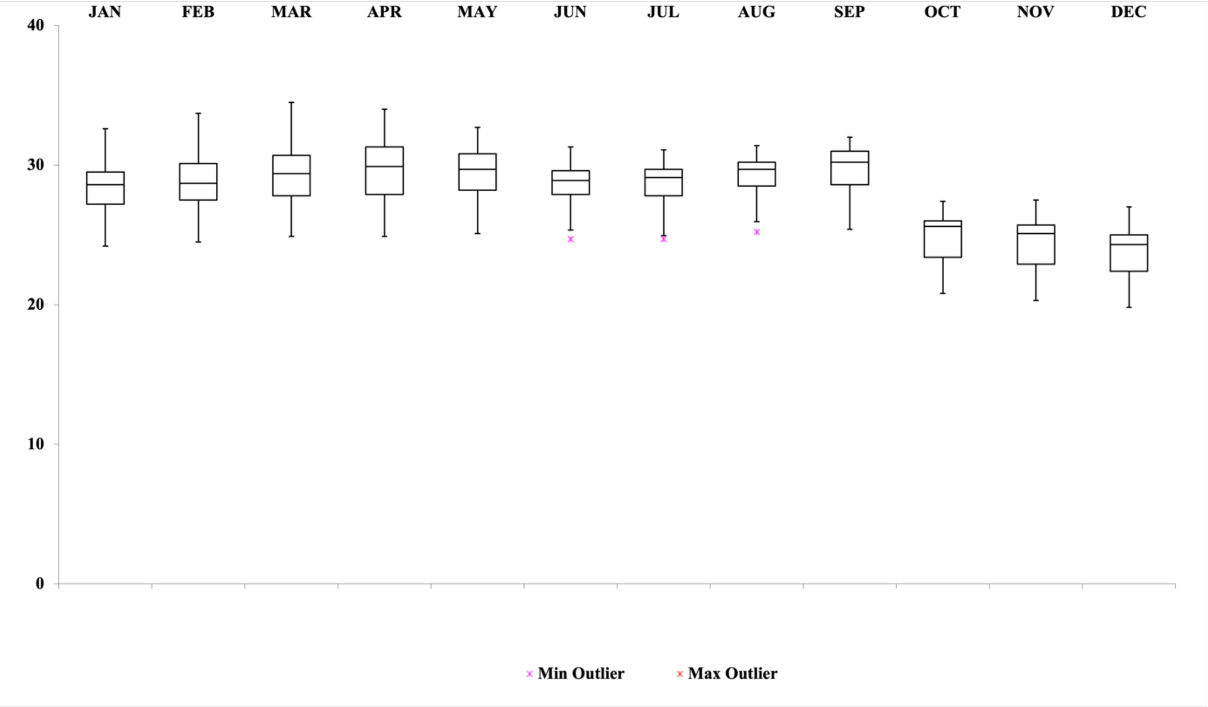

5.1.3.3. MinMT [°C]

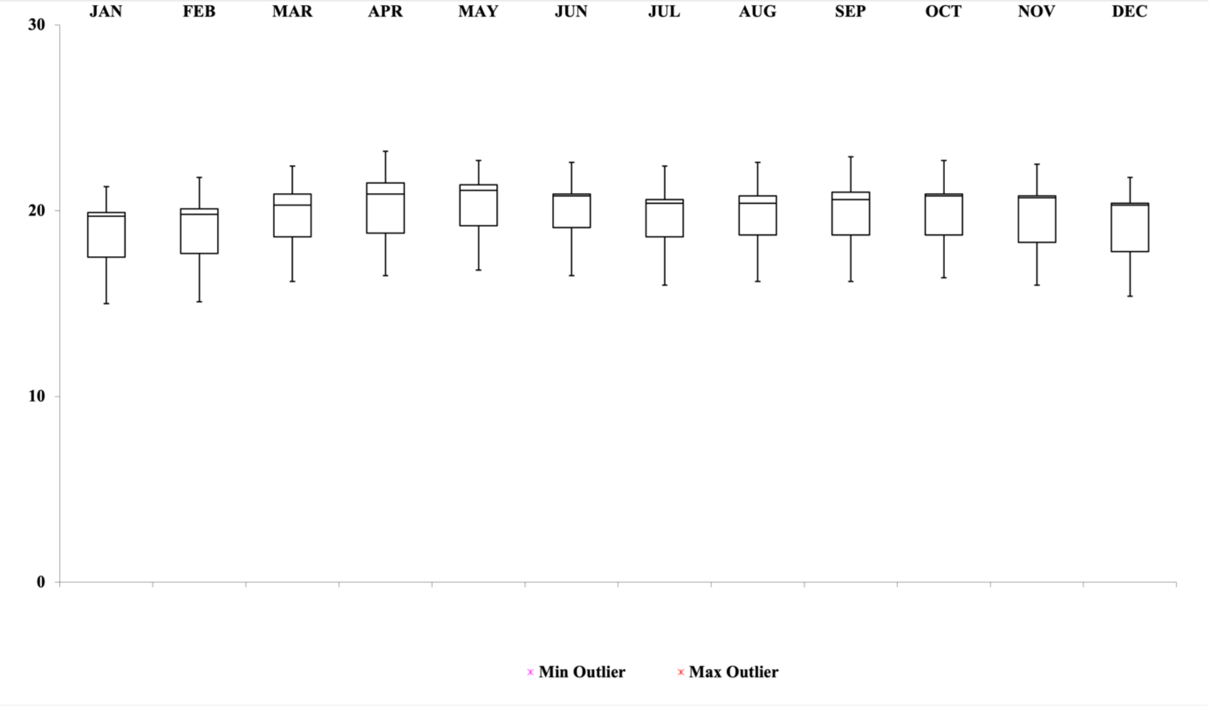

5.1.3.4. MMP [mm]

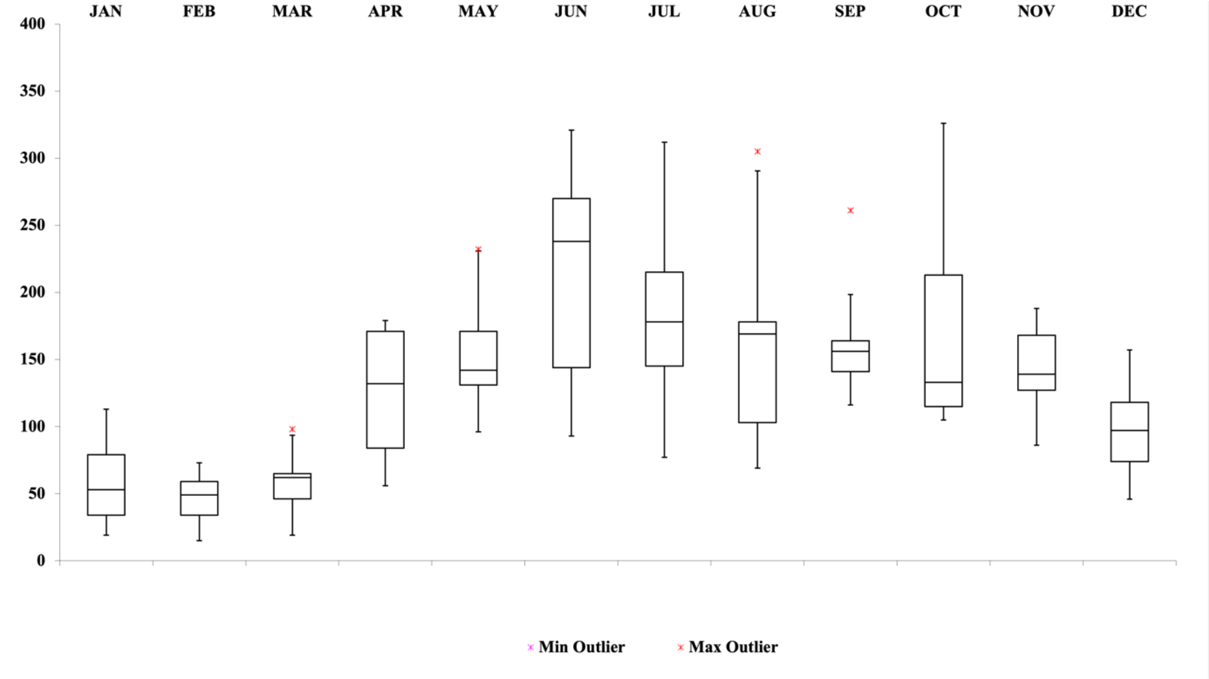

5.2. Species *Piranhea mexicana* (Standl., 1927) Radcl.-Sm., 1996

5.2.1. Köppen profile, distribution, and climate map – GBIF occurrences of *Piranhea mexicana*; all specimens (herbarium and human observation) excluding duplicate occurrences (n = 123; preserved specimens, n = 58).

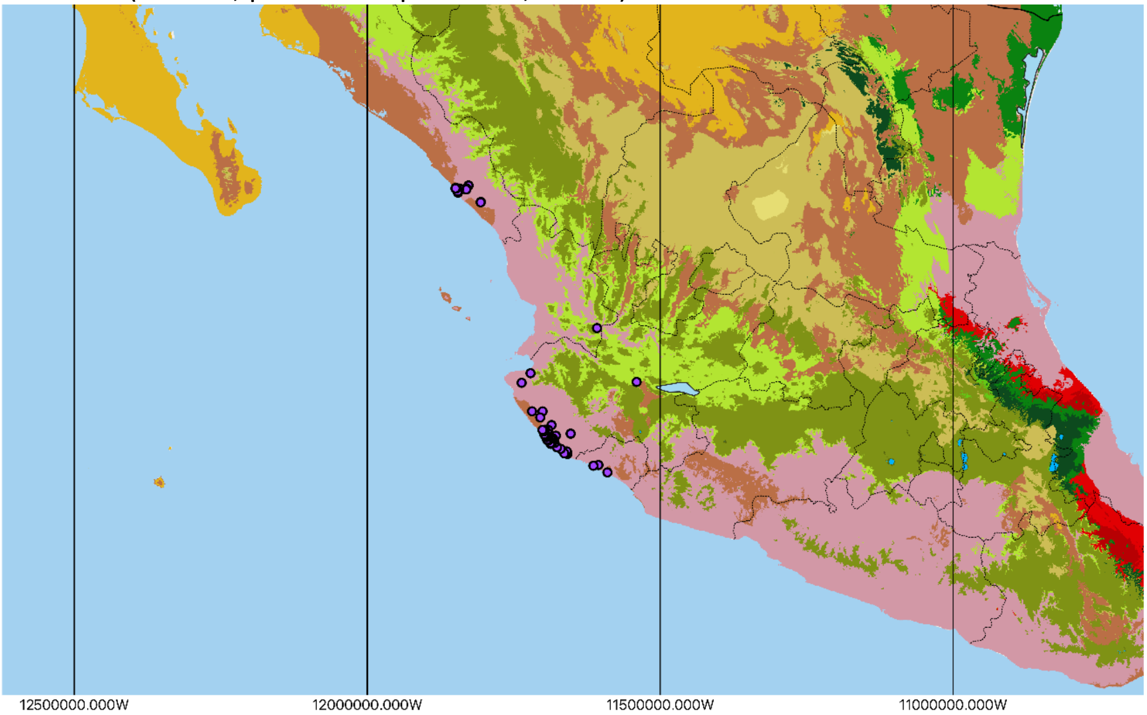

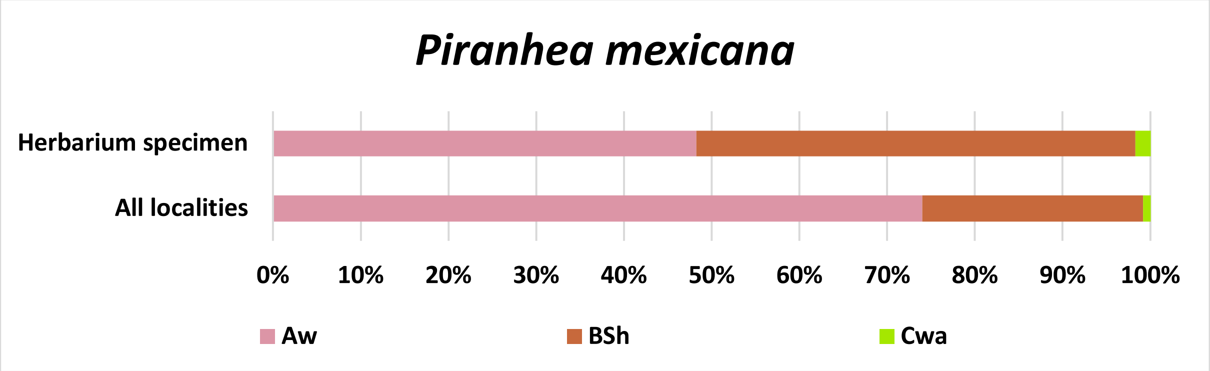

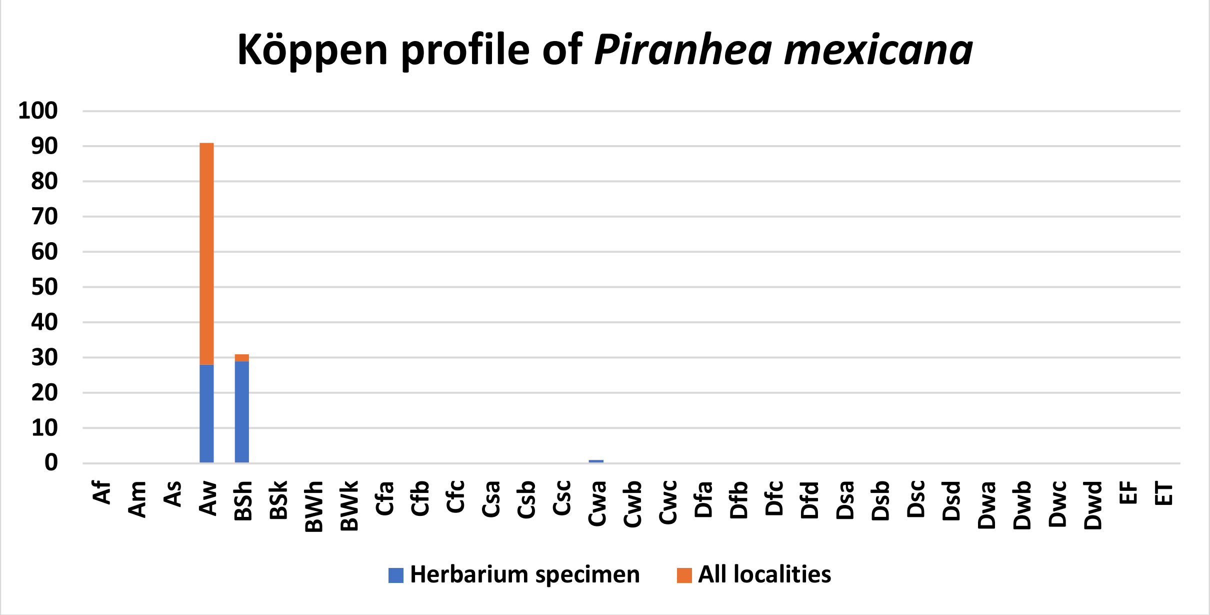

5.2.2. Biome profile, distribution, and climate map – GBIF occurrences of *Piranhea mexicana*; all specimens (herbarium and human observation) excluding duplicate occurrences (n = 122; preserved specimens, n = 57).

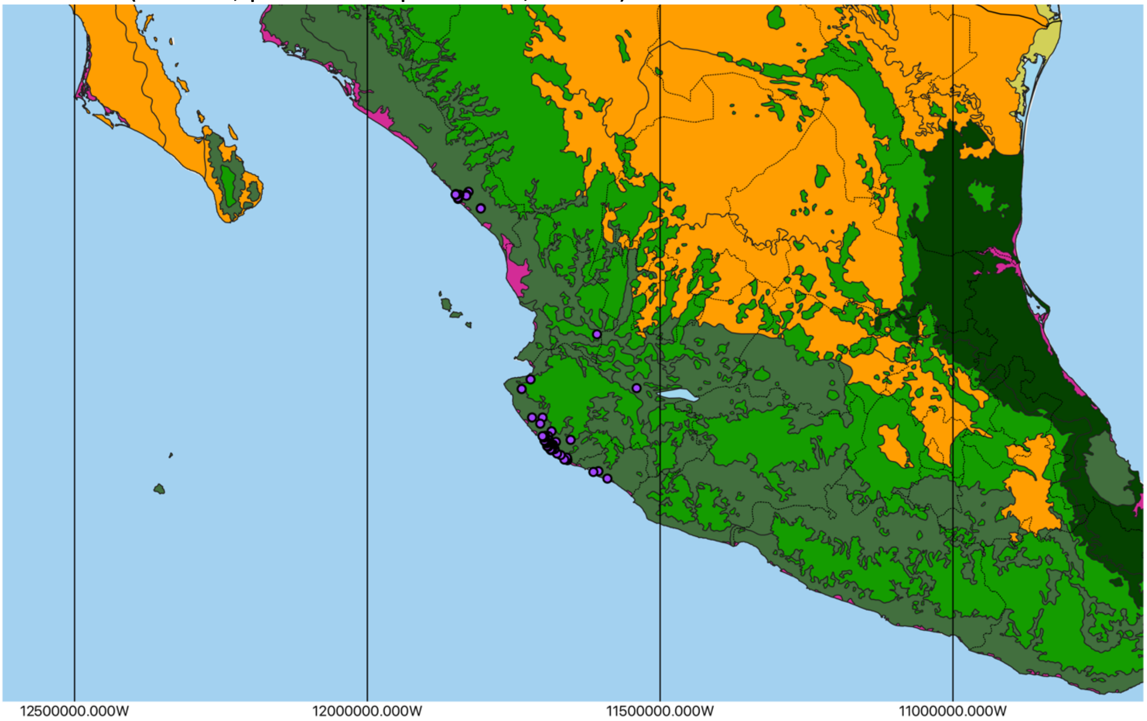

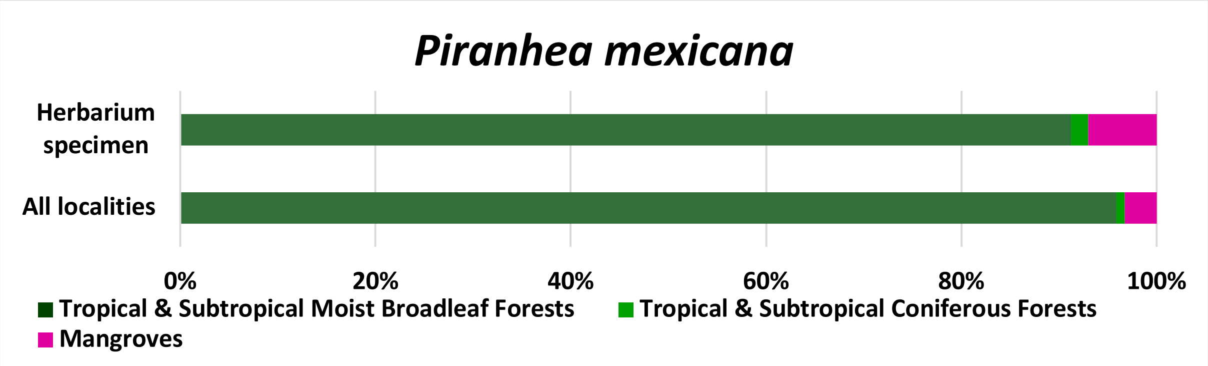

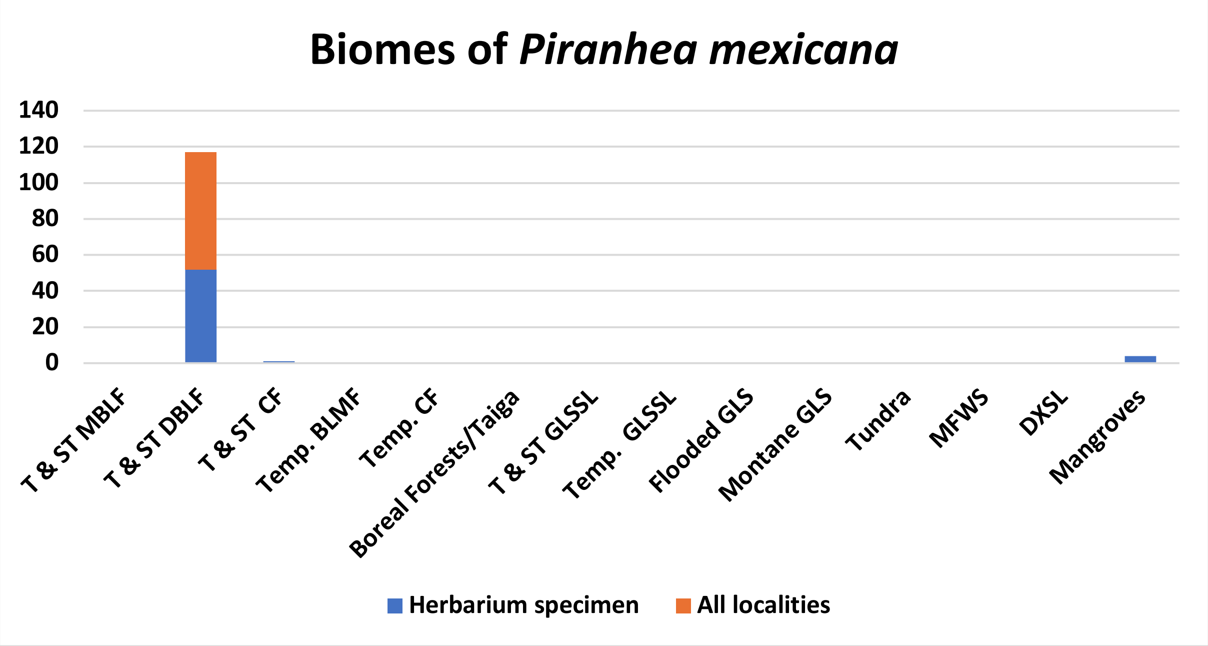

5.2.3. Climate graphs - based on 122 *Piranhea mexicana* occurrences in GBIF

5.2.3.1. MMT [°C]

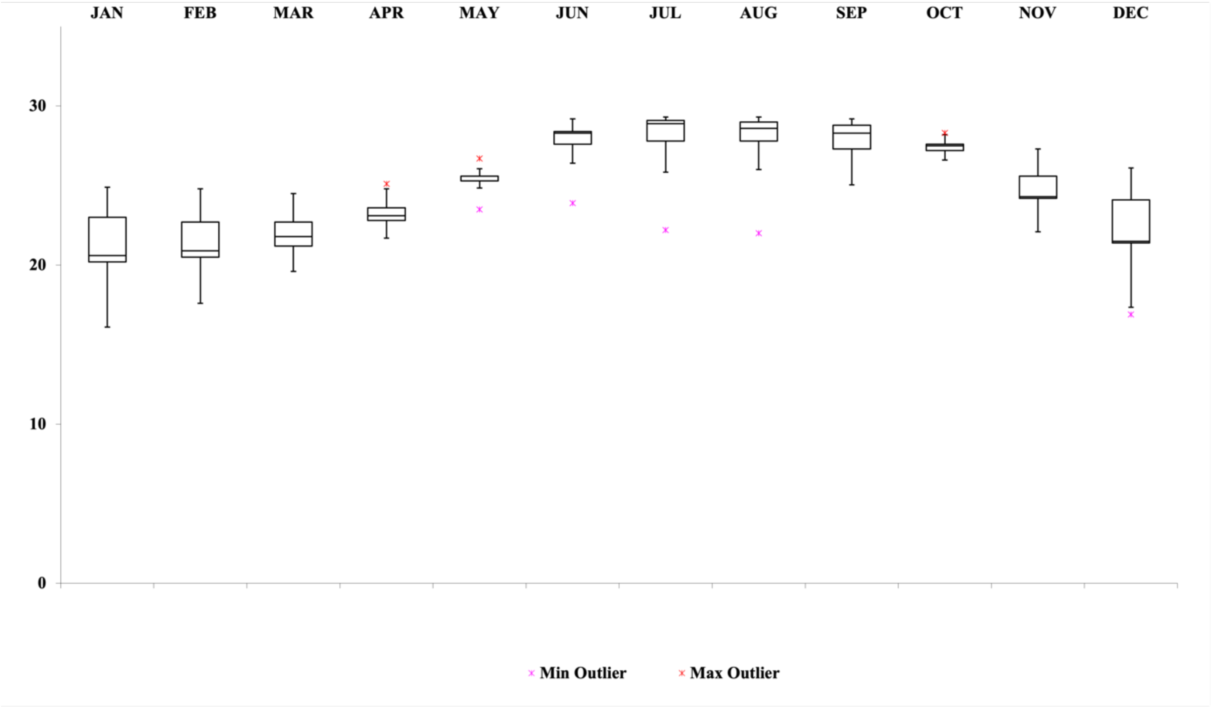

5.2.3.2. MaxMT [°C]

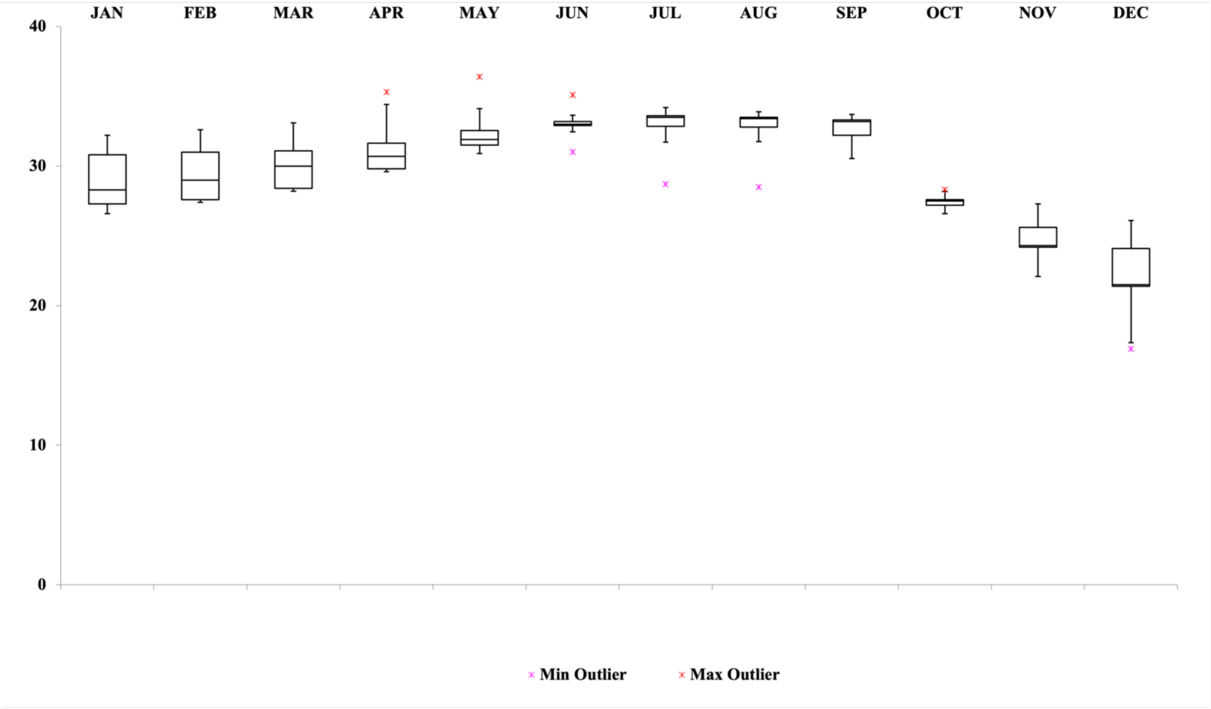

5.2.3.3. MinMT [°C]

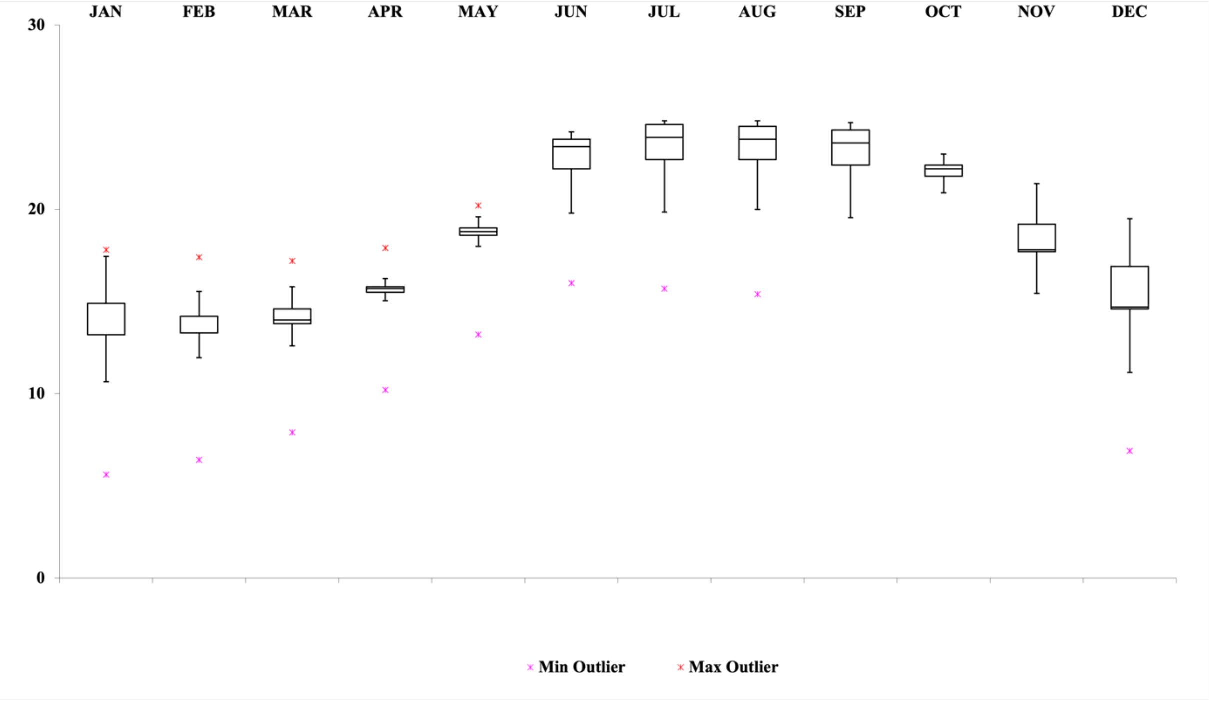

5.2.3.4. MMP [mm]

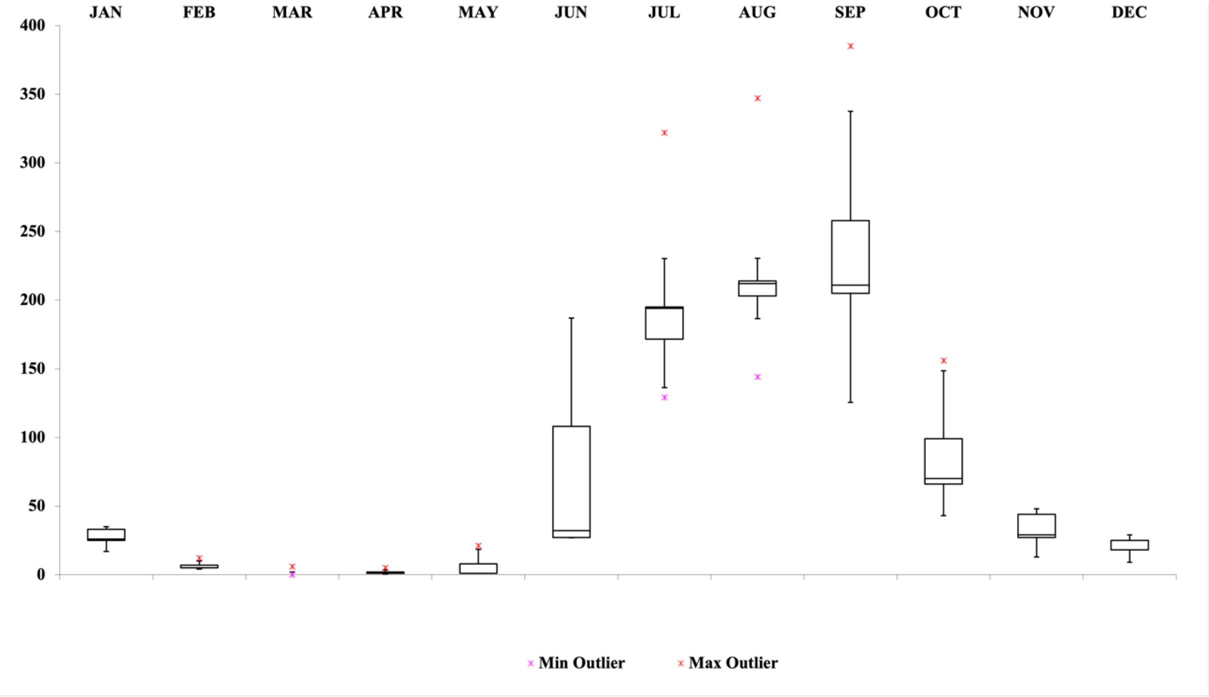

5.3. Species *Piranhea securinega* Radcl.-Sm. et. Ratter, 1996

5.3.1. Köppen profile, distribution, and climate map – GBIF occurrences of *Piranhea securinega*; herbarium specimens excluding duplicate occurrences (preserved specimens, n = 21).

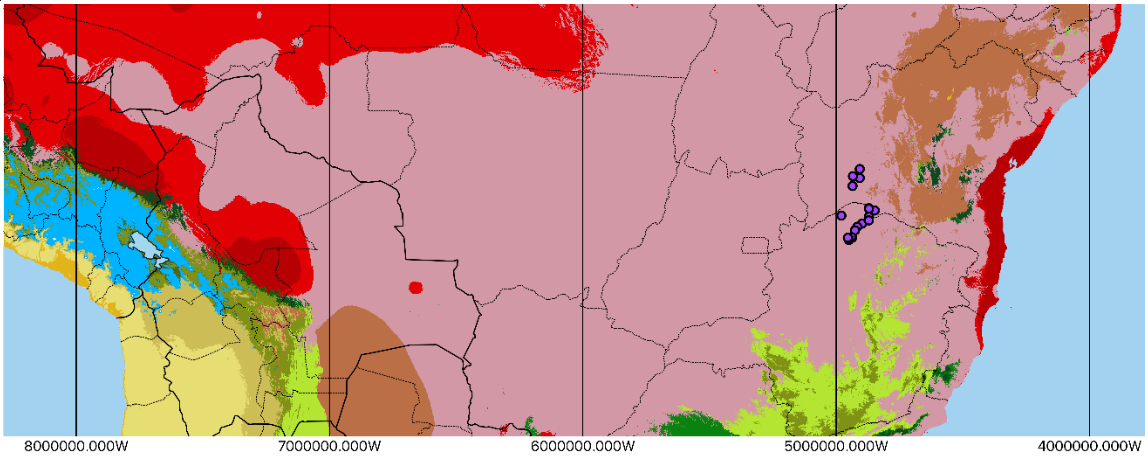

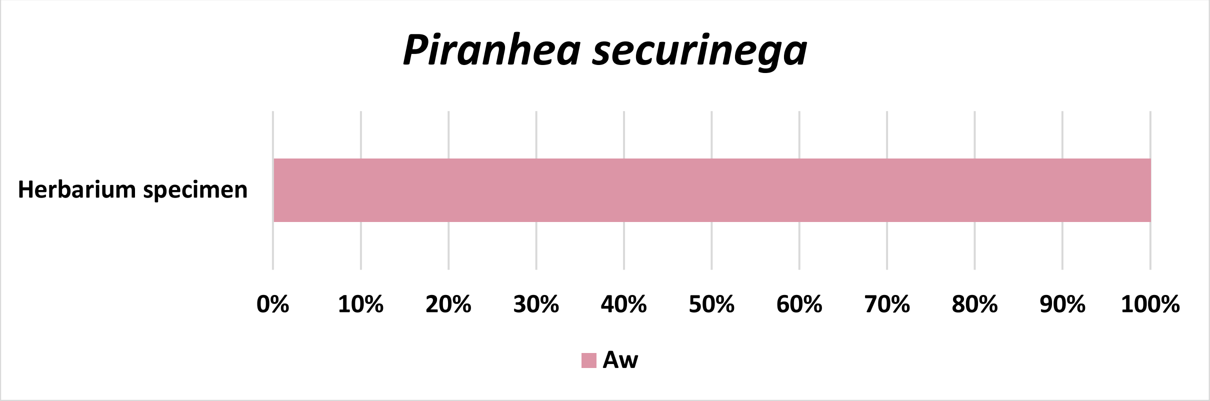

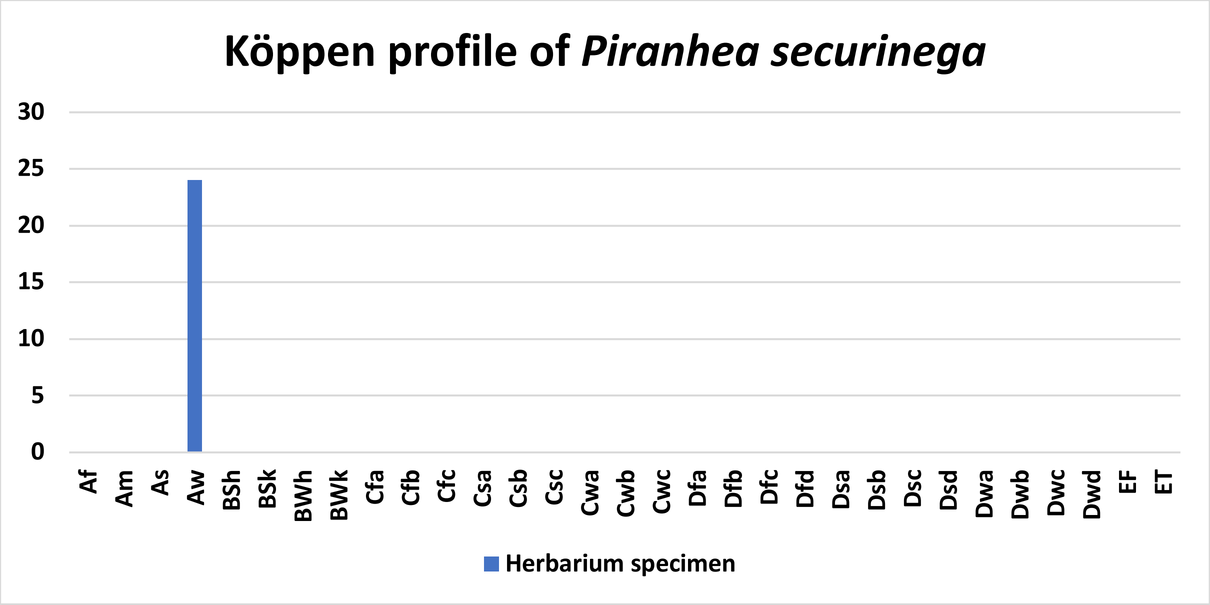

5.3.2. Biome profile, distribution, and climate map – GBIF occurrences of *Piranhea securinega*; herbarium specimens excluding duplicate occurrences (preserved specimens, n = 21).

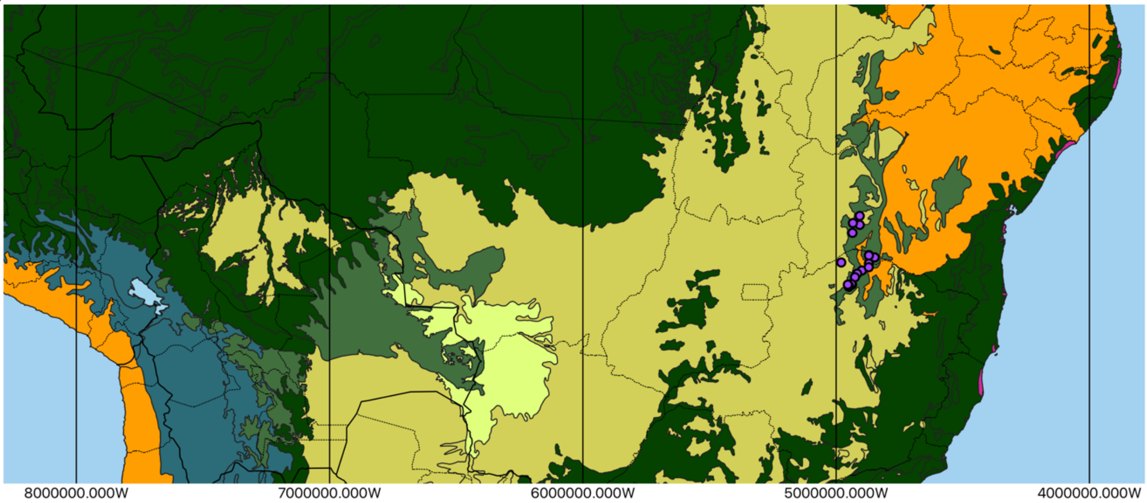

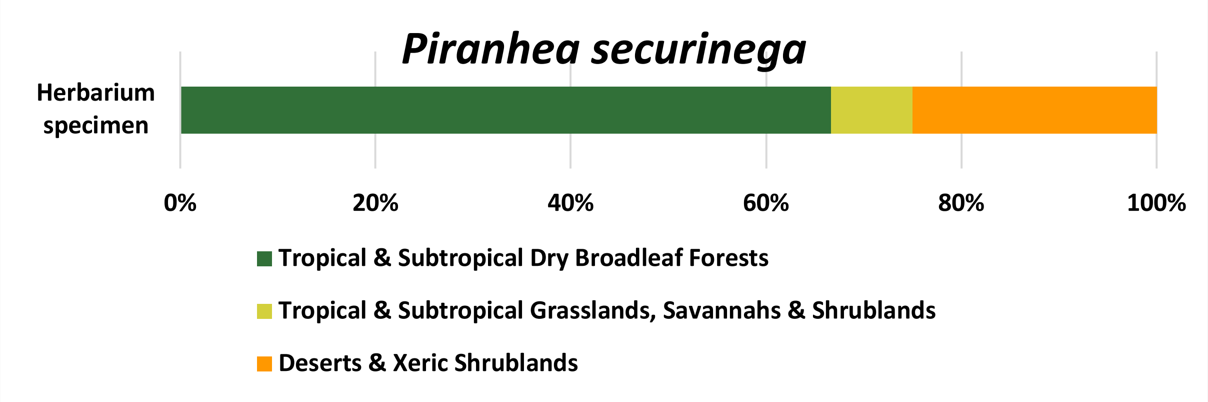

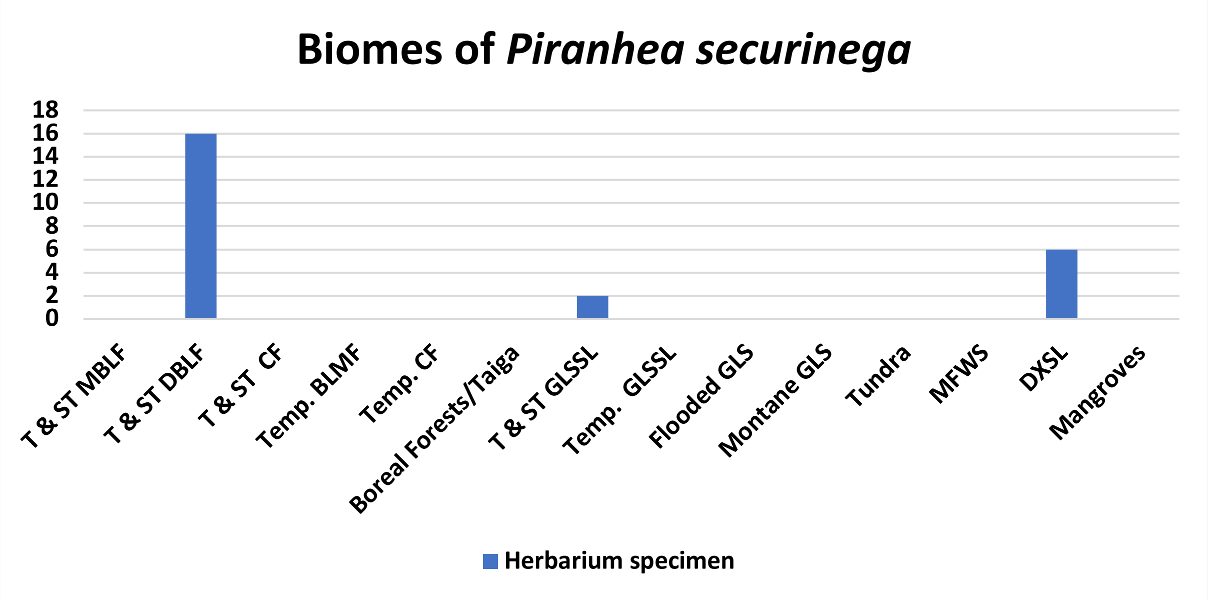

5.3.3. Climate graphs - based on 21 *Piranhea securinega* occurrences in GBIF

5.3.3.1. MMT [°C]

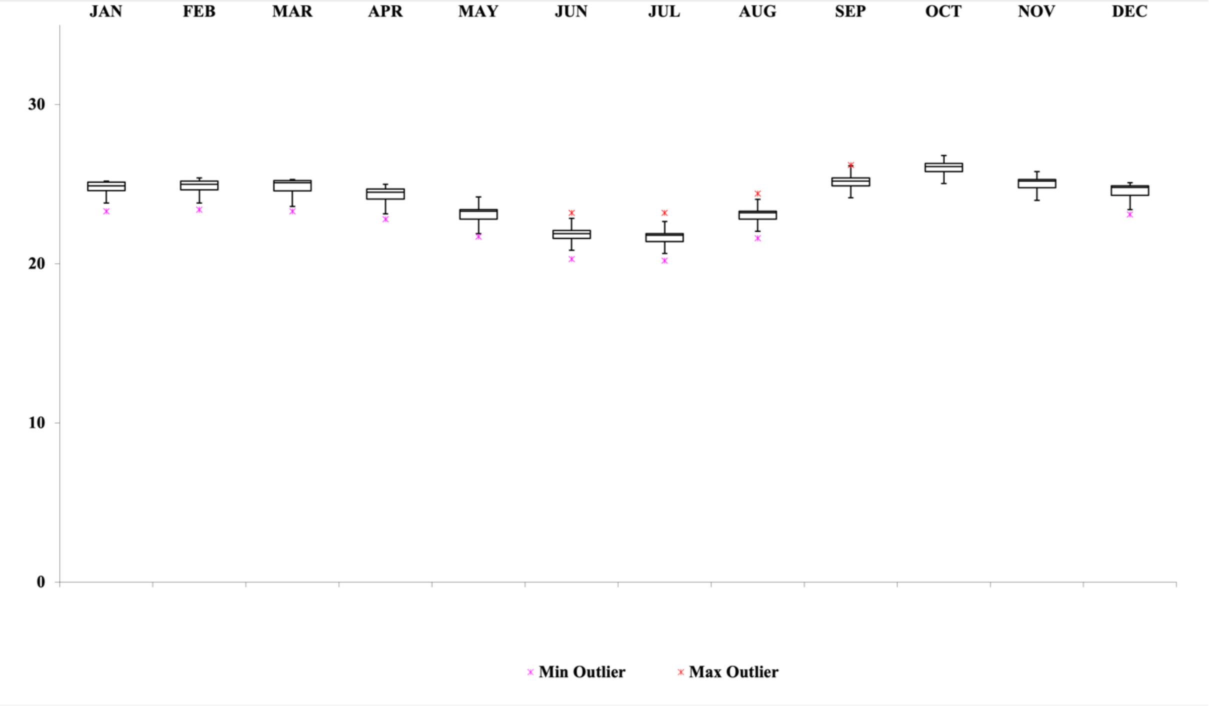

5.3.3.2. MaxMT [°C]

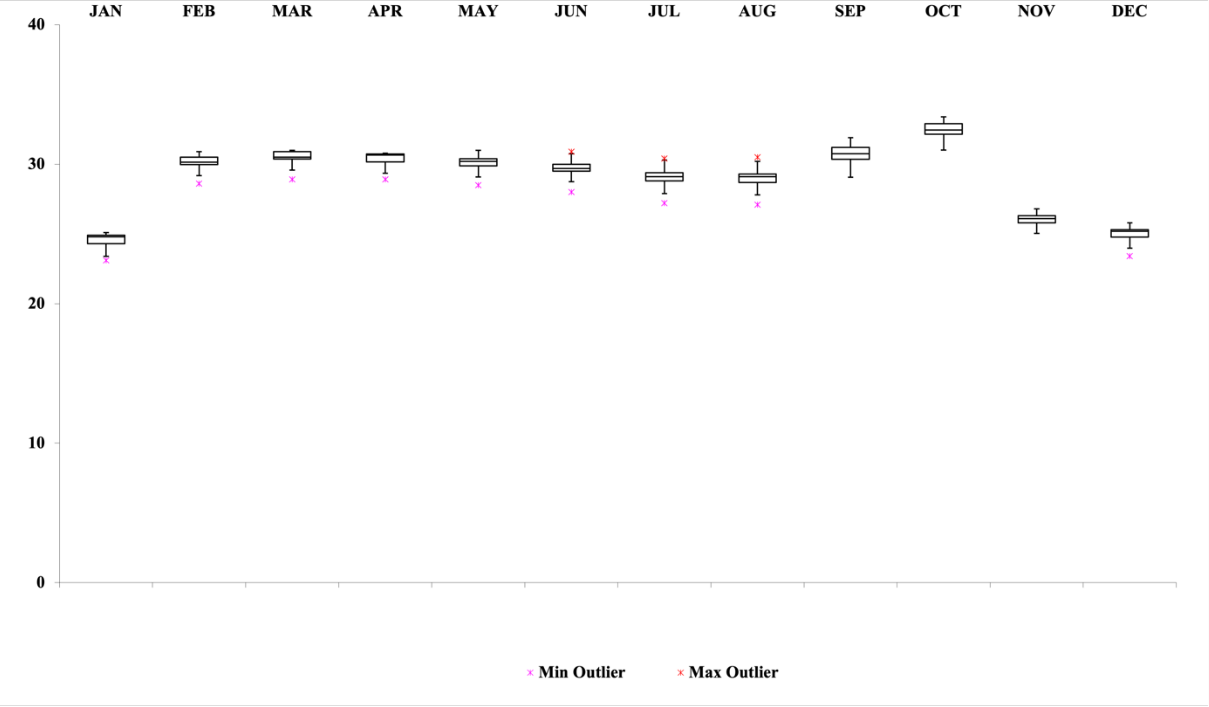

5.3.3.3. MinMT [°C]

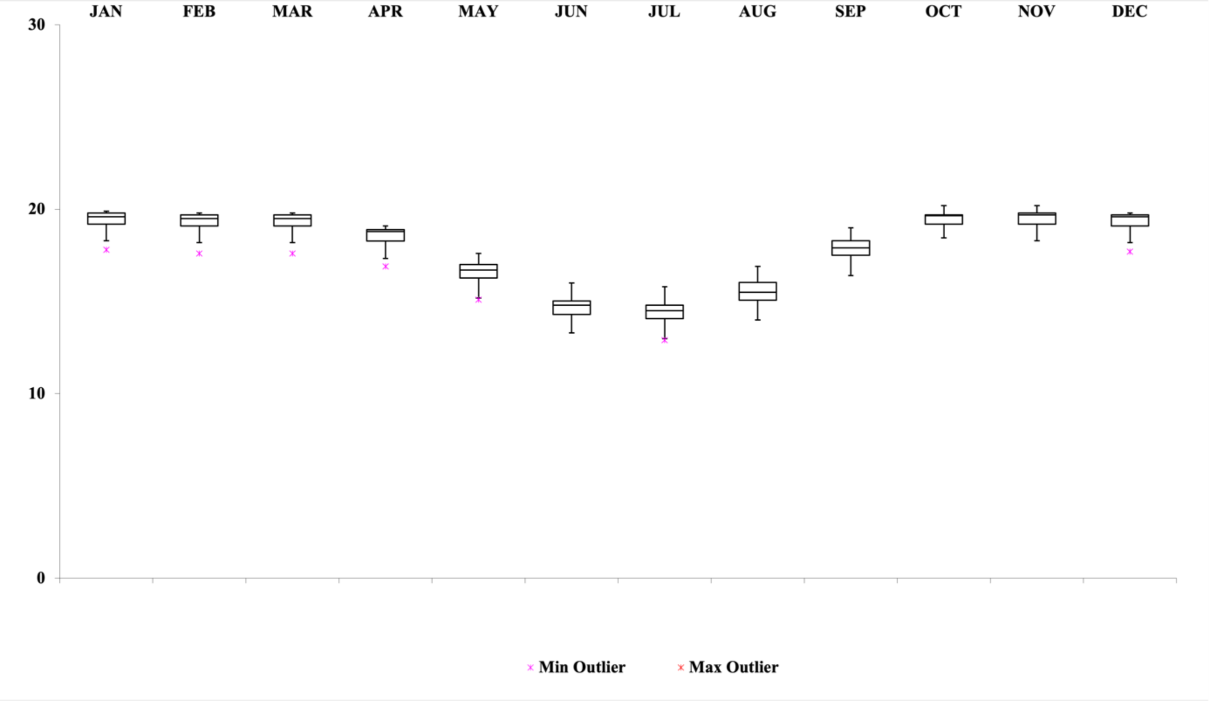

5.3.3.4. MMP [mm]

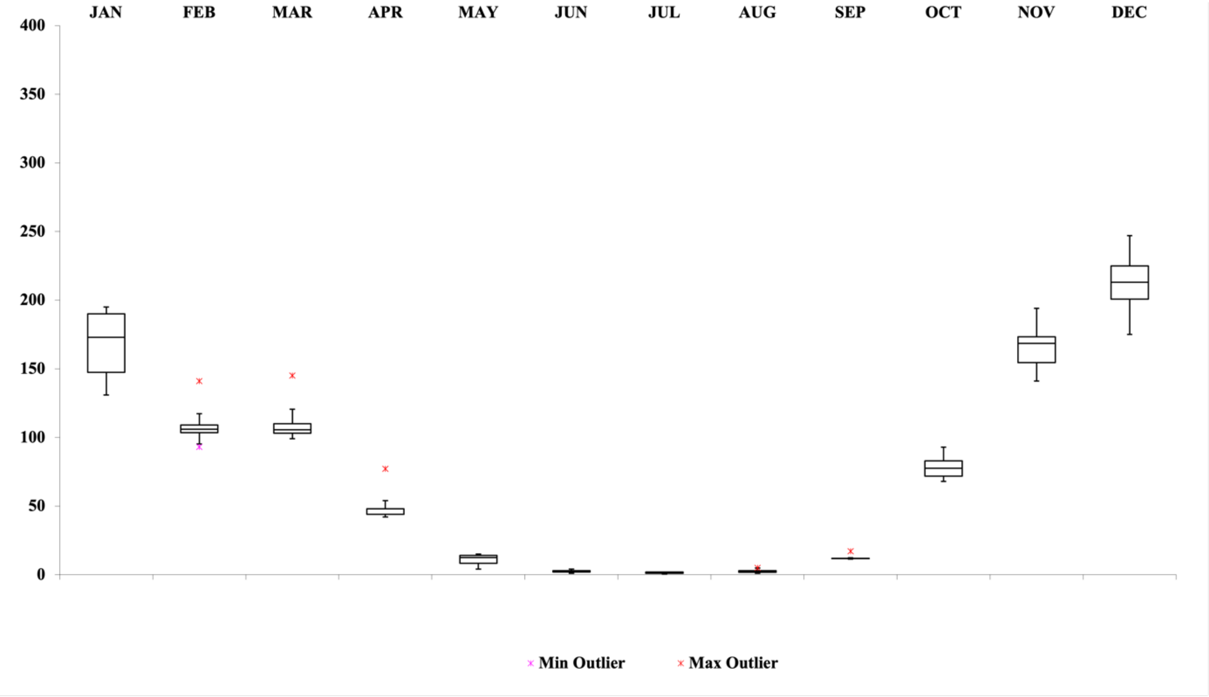

5.4. Species *Piranhea trifoliolata* Baill., 1866

5.4.1. Köppen profile, distribution, and climate map – GBIF occurrences of *Piranhea trifoliolata*; all specimens (herbarium and human observation) excluding duplicate occurrences (n = 225; preserved specimens, n = 221).

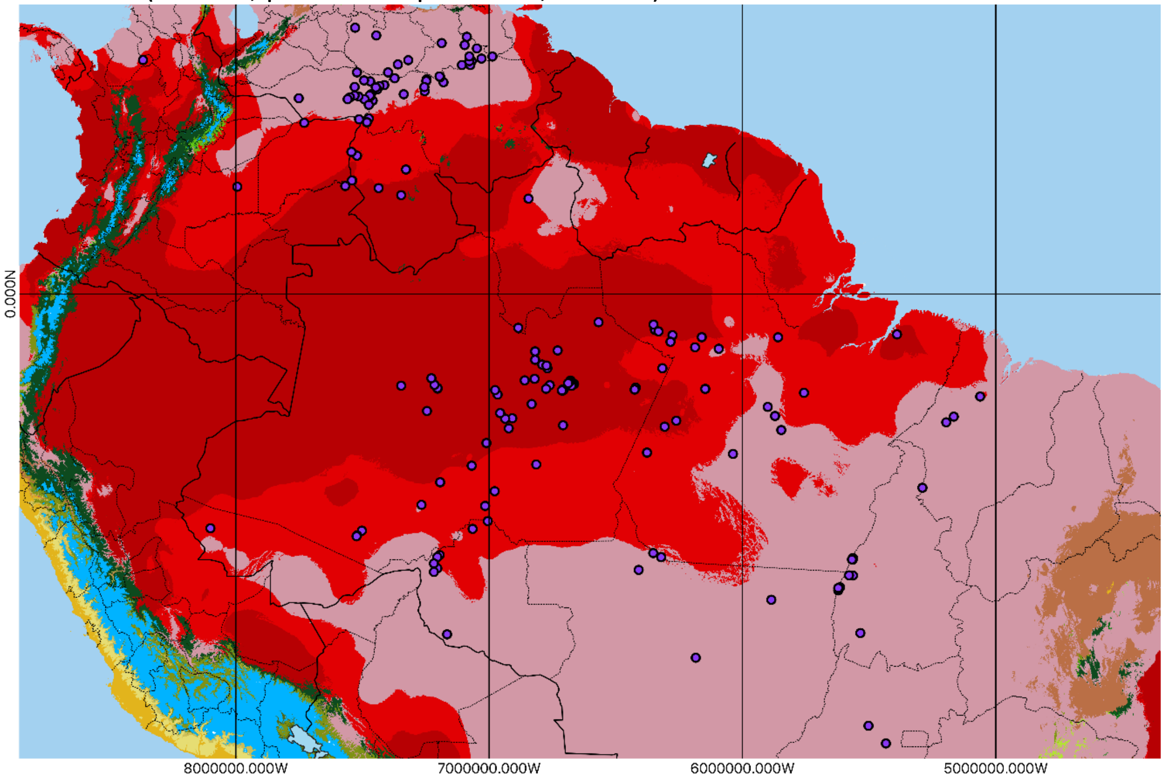

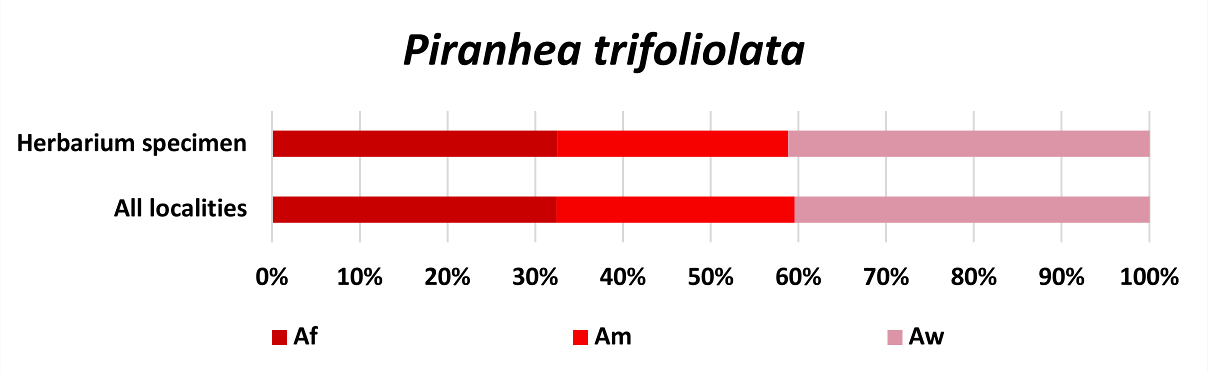

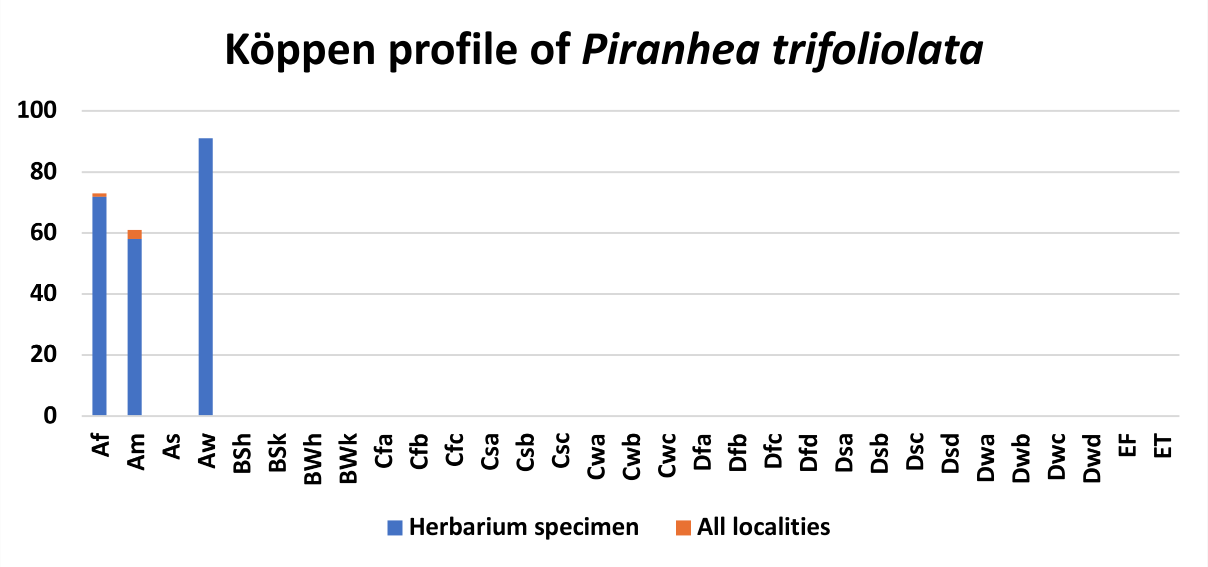

5.4.2. Biome profile, distribution, and climate map – GBIF occurrences of *Piranhea trifoliolata*; all specimens (herbarium and human observation) excluding duplicate occurrences (n = 225; preserved specimens, n = 221).

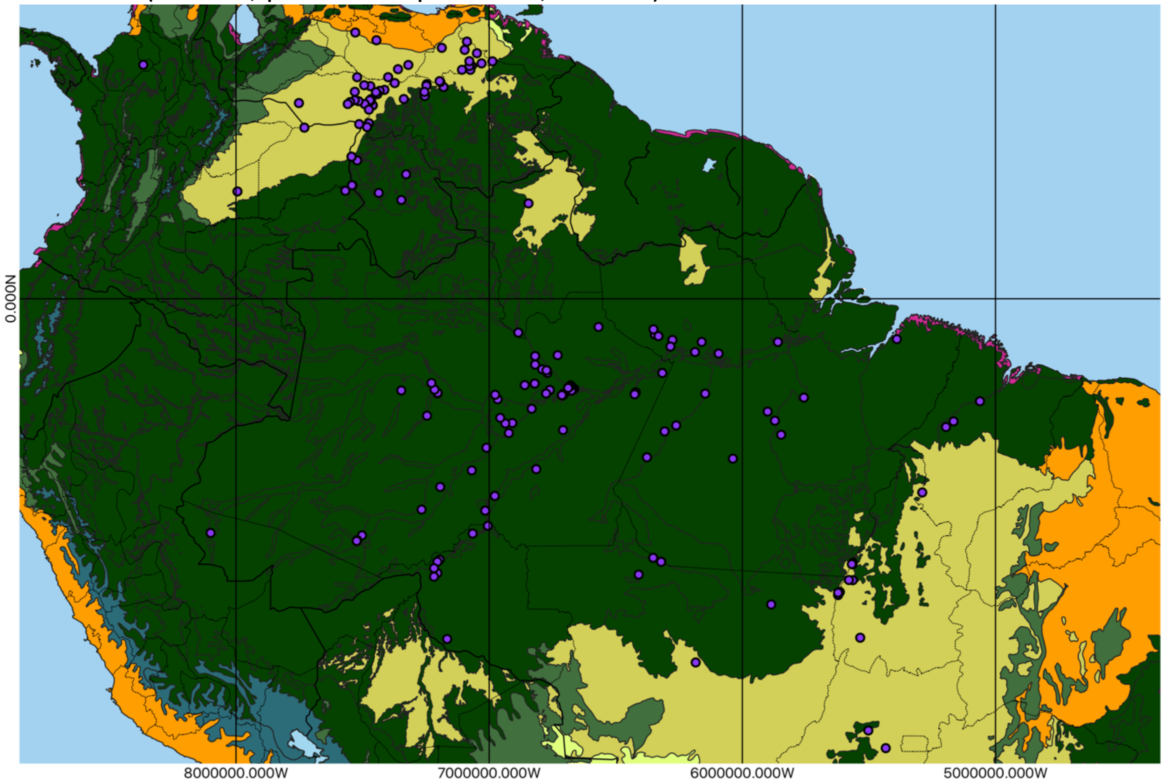

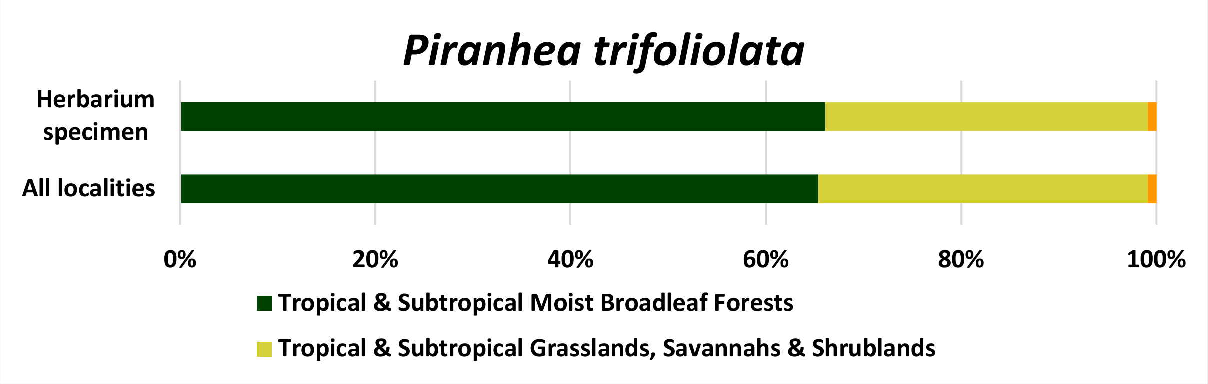

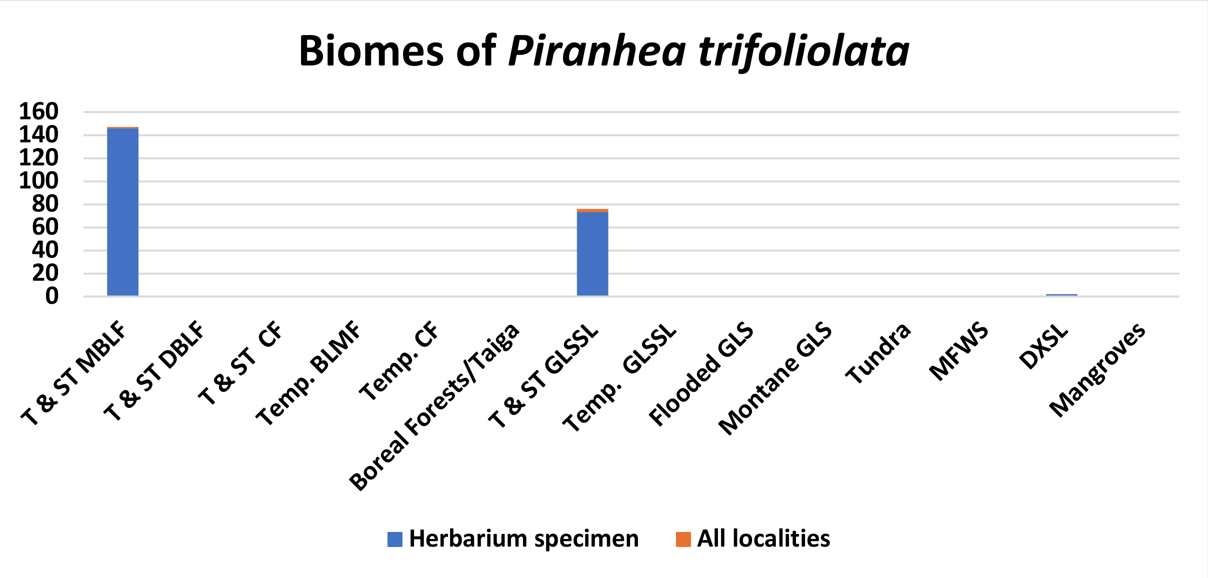

5.4.3. Climate graphs - based on 225 *Piranhea trifoliolata* occurrences in GBIF

5.4.3.1. MMT [°C]

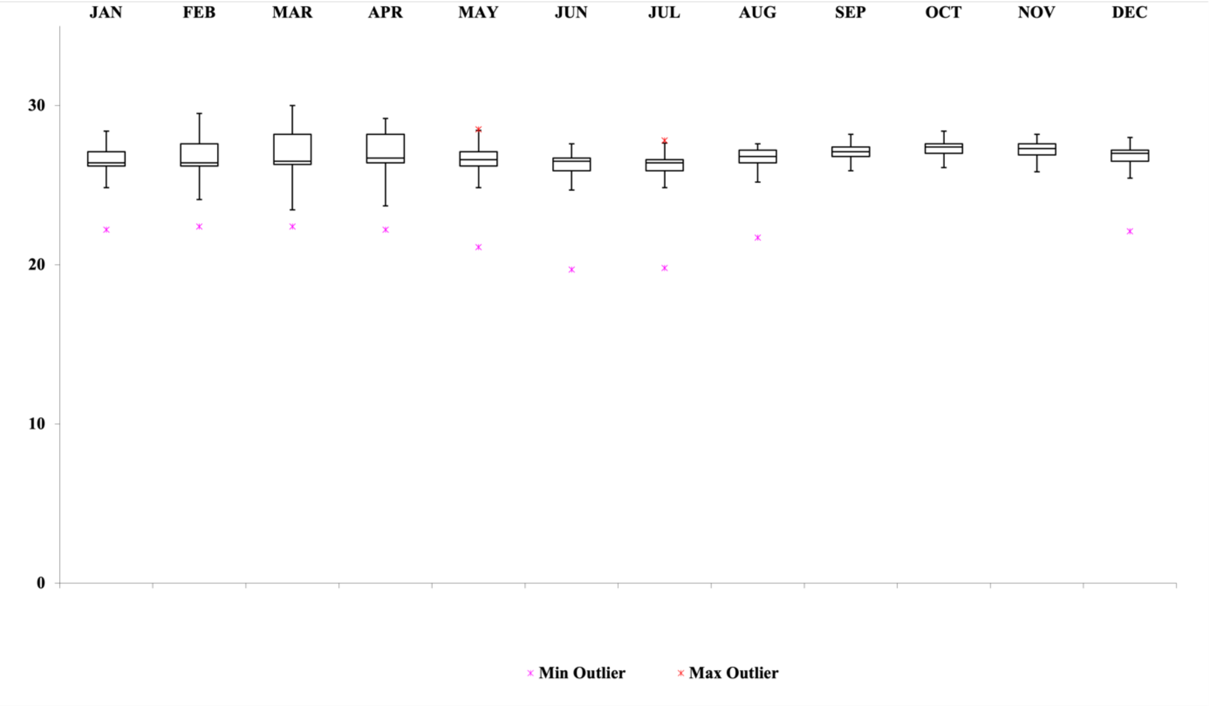

5.4.3.2. MaxMT [°C]

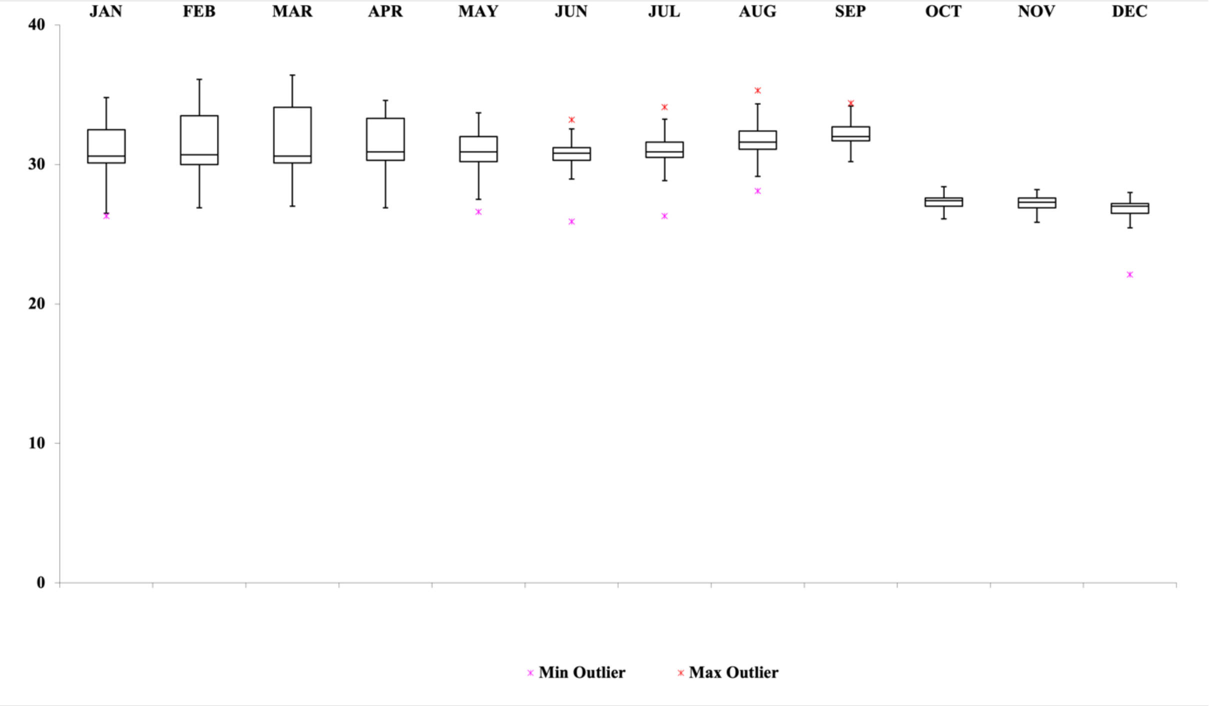

5.4.3.3. MinMT [°C]

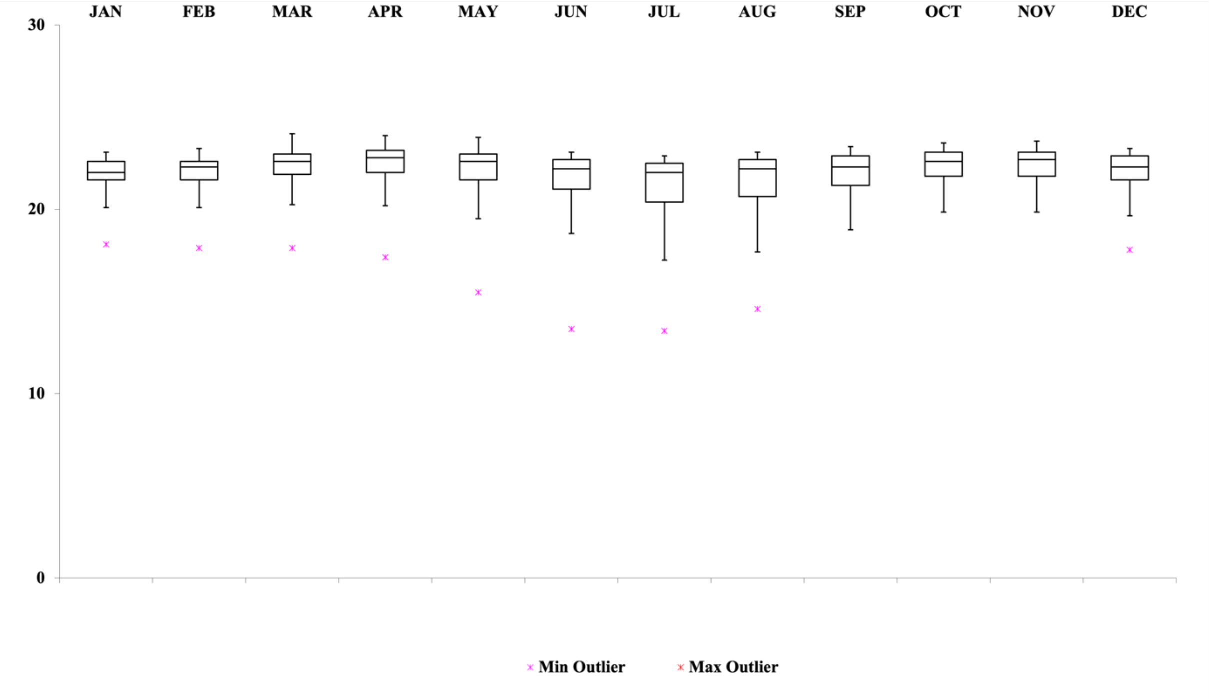

5.4.3.4. MMP [mm]

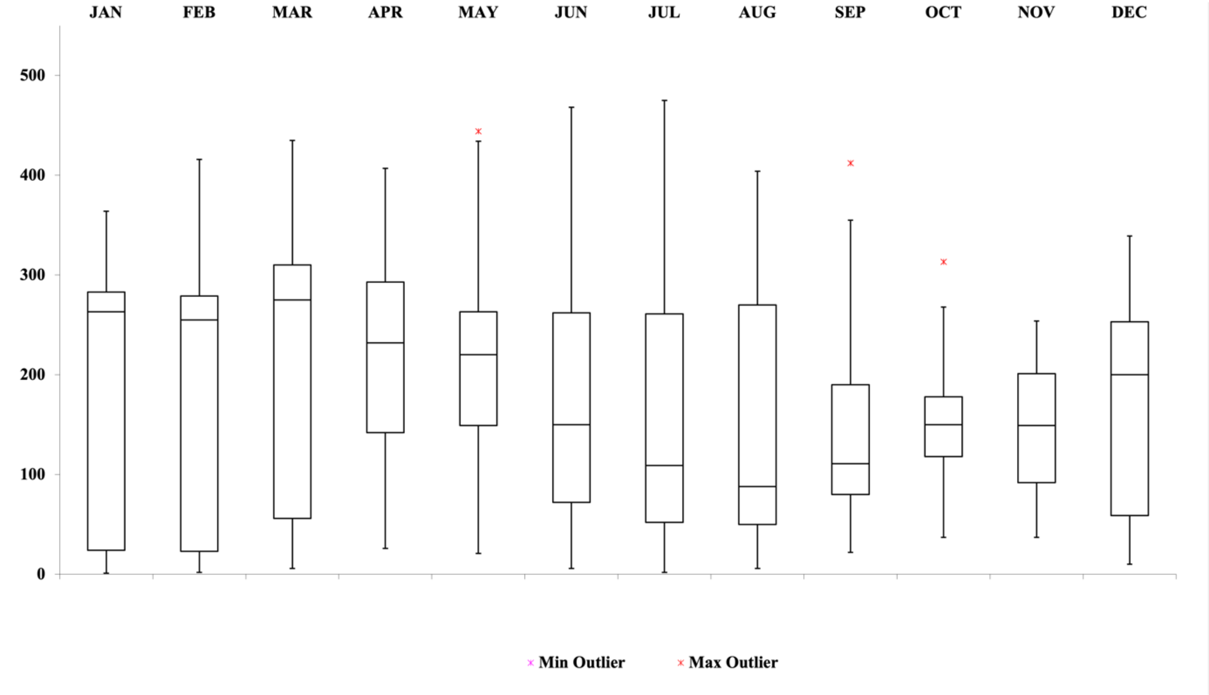

6. Genus *Tetracoccus* Engelm. ex Parry, 1885

Genus distribution (Köppen-Geiger climates)

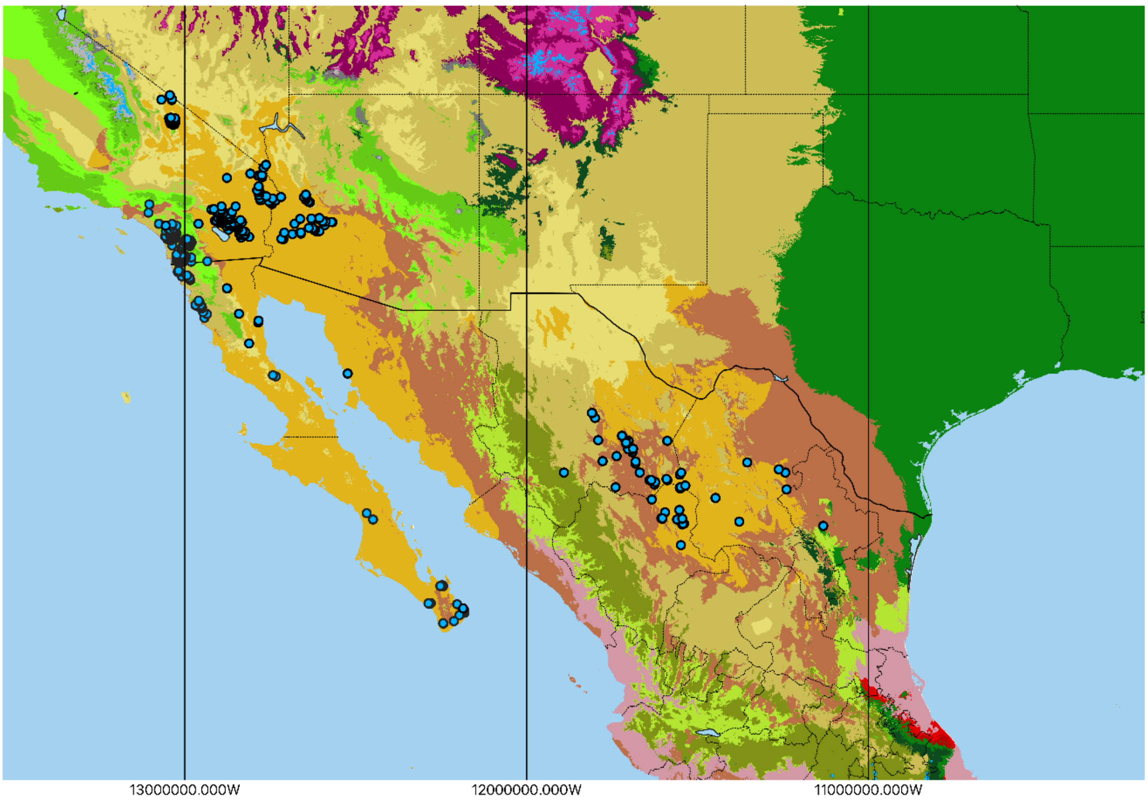

Genus distribution (Biomes)

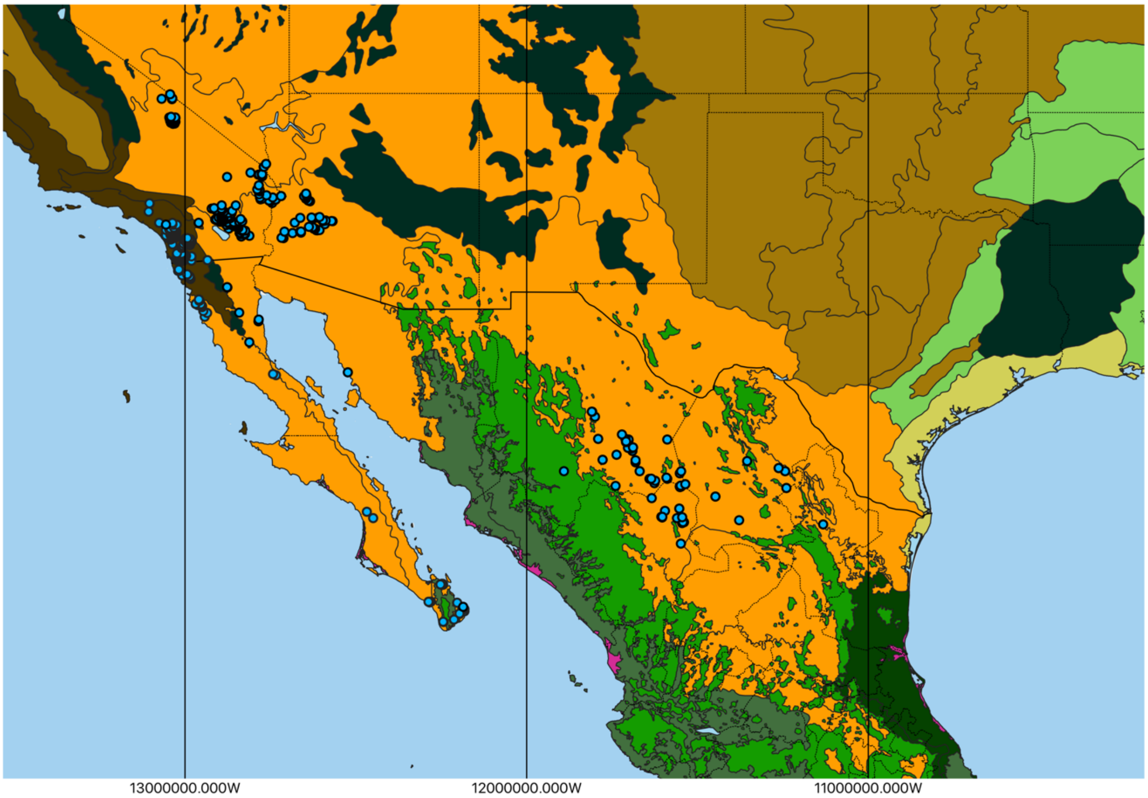

6.1. Species *Tetracoccus capensis* (I.M.Johnst., 1922) Croizat, 1942

6.1.1. Köppen profile, distribution, and climate map – GBIF occurrences of *Tetracoccus capensis*; all specimens (herbarium and human observation) excluding duplicate occurrences (n = 35; preserved specimens, n = 17).

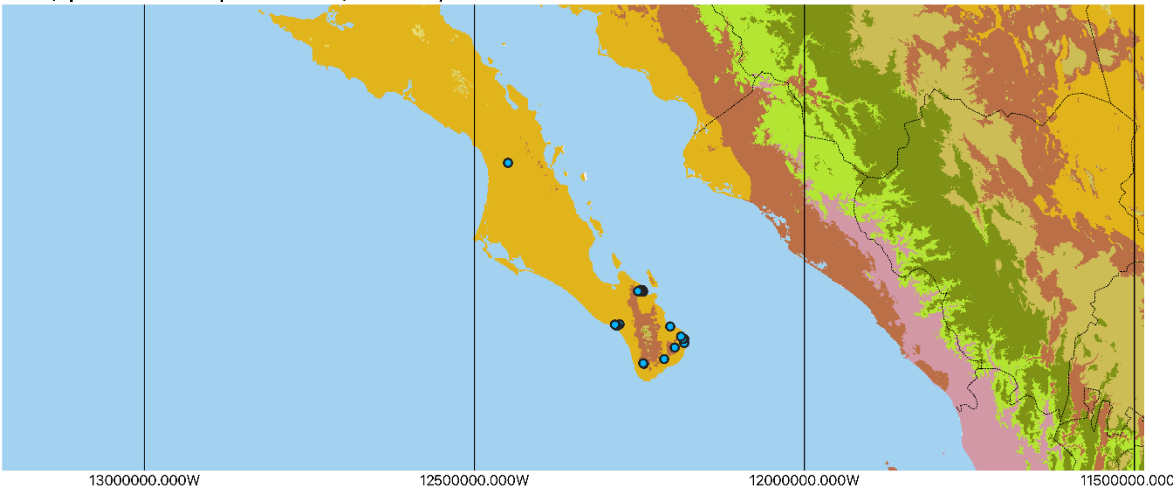

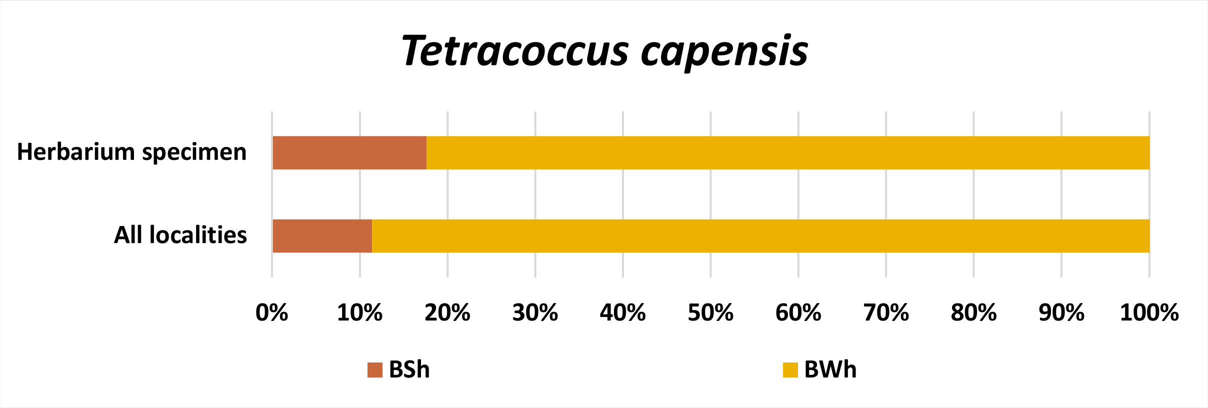

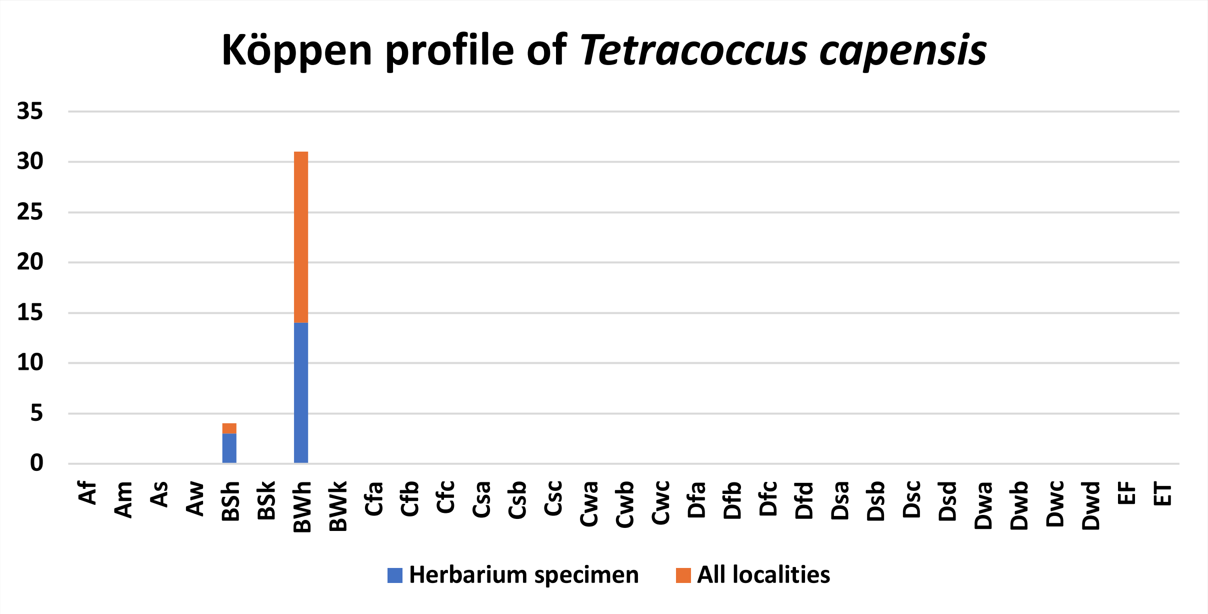

6.1.2. Biome profile, distribution, and climate map – GBIF occurrences of *Tetracoccus capensis*; all specimens (herbarium and human observation) excluding duplicate occurrences (n = 35; preserved specimens, n = 17).

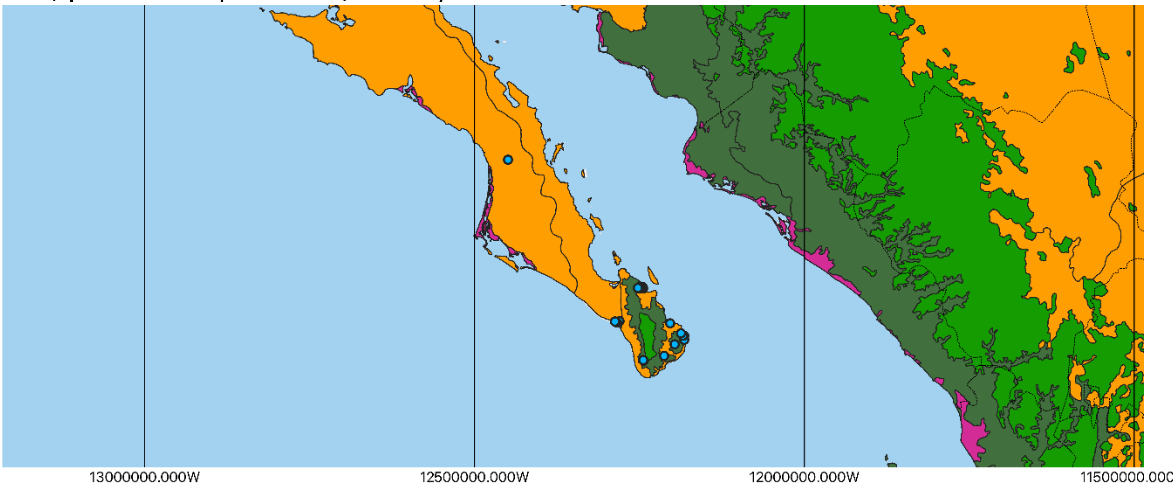

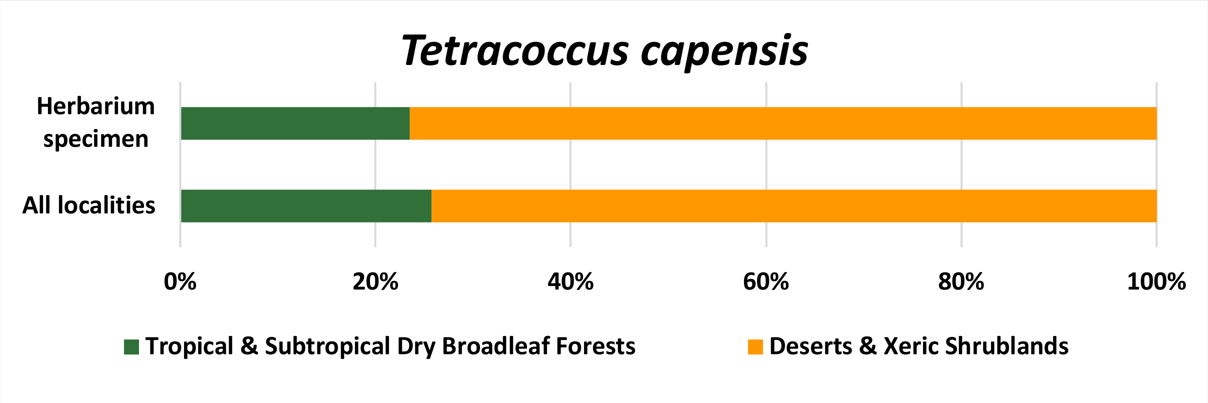

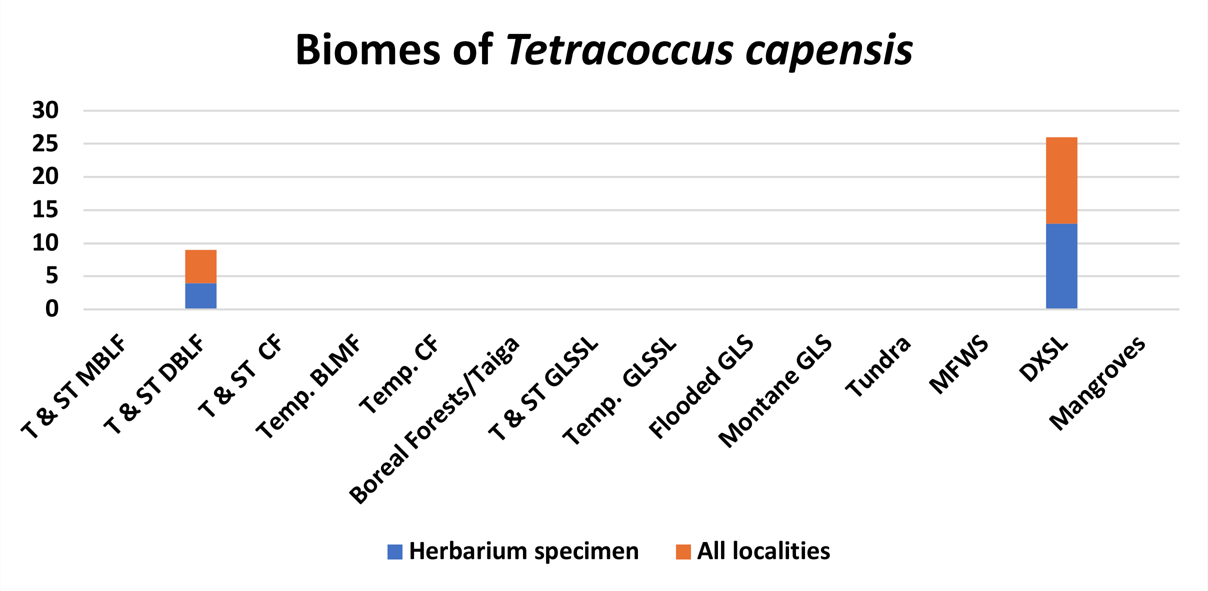

6.1.3. Climate graphs – based on 35 *Tetracoccus capensis* occurrences in GBIF

6.1.3.1. MMT [°C]

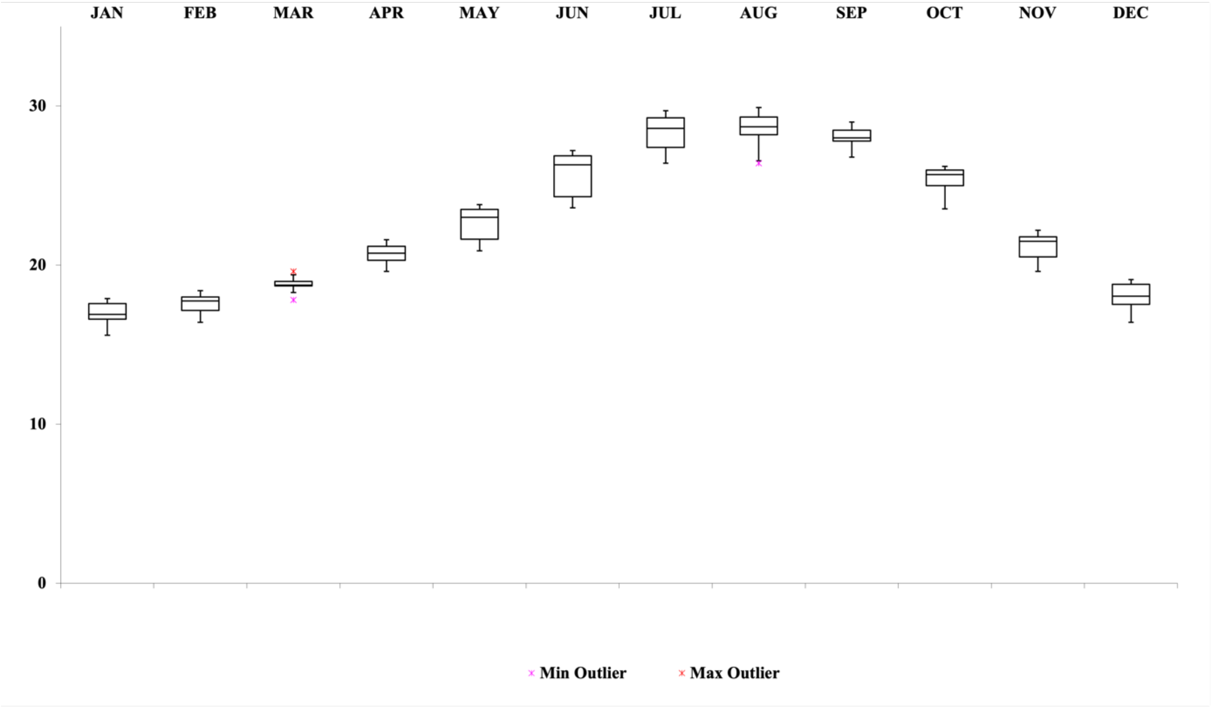

6.1.3.2. MaxMT [°C]

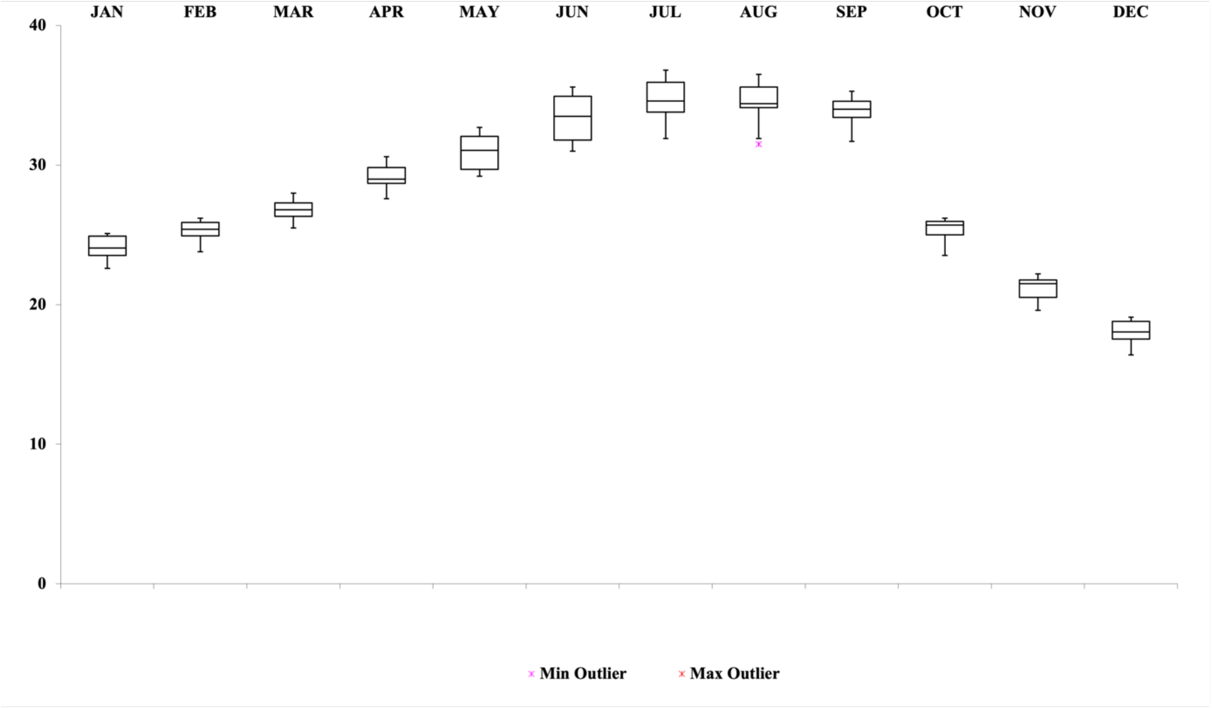

6.1.3.3. MinMT [°C]

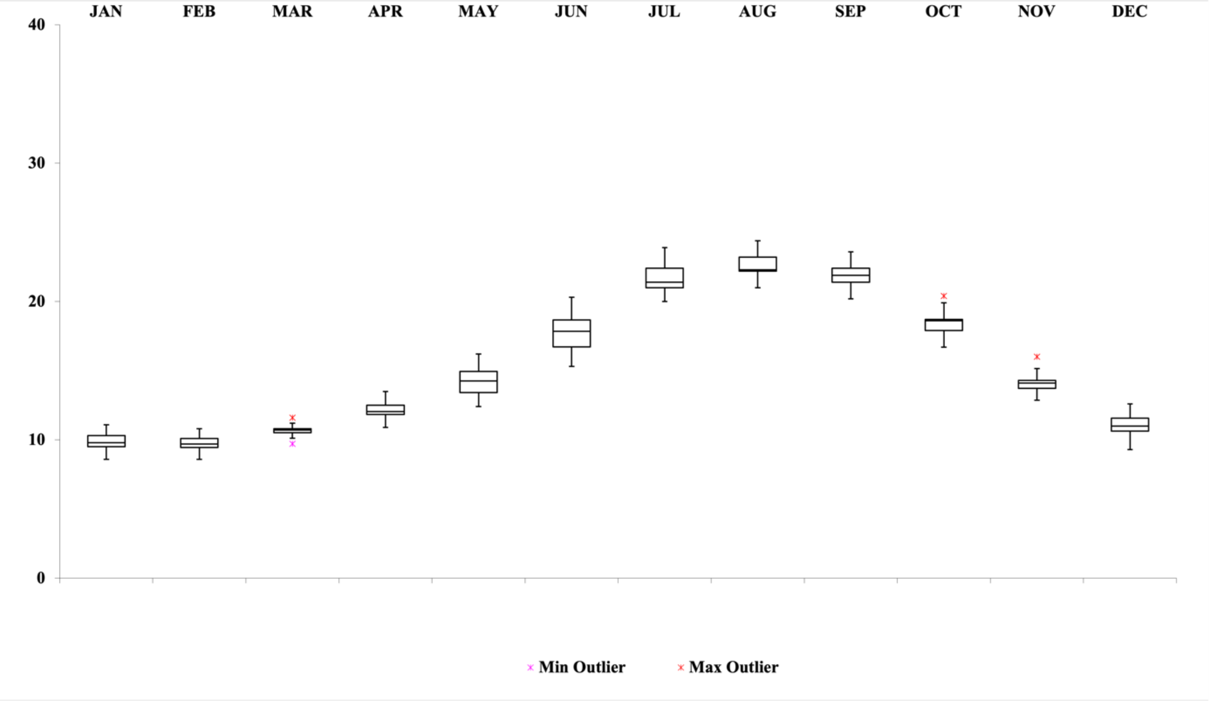

6.1.3.4. MMP [mm]

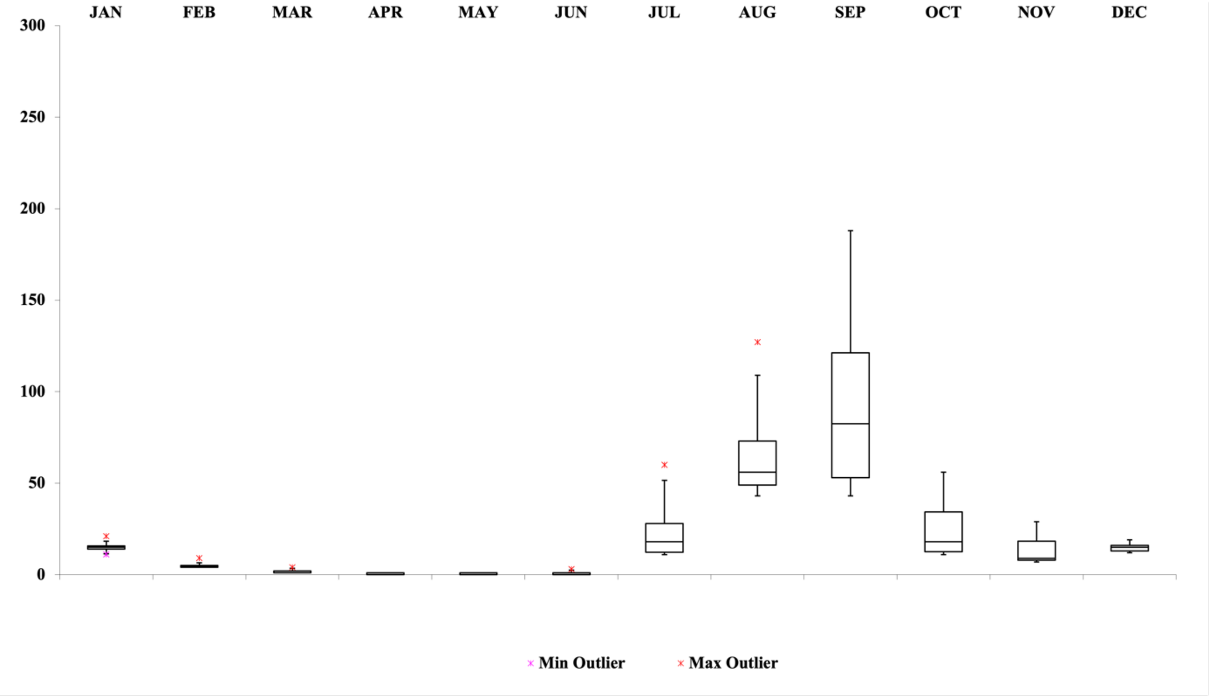

6.2. Species *Tetracoccus dioicus* Parry, 1885

6.2.1. Köppen profile, distribution, and climate map – GBIF occurrences of *Tetracoccus dioicus*; all specimens (herbarium and human observation) excluding duplicate occurrences (n = 328; preserved specimens, n = 136).

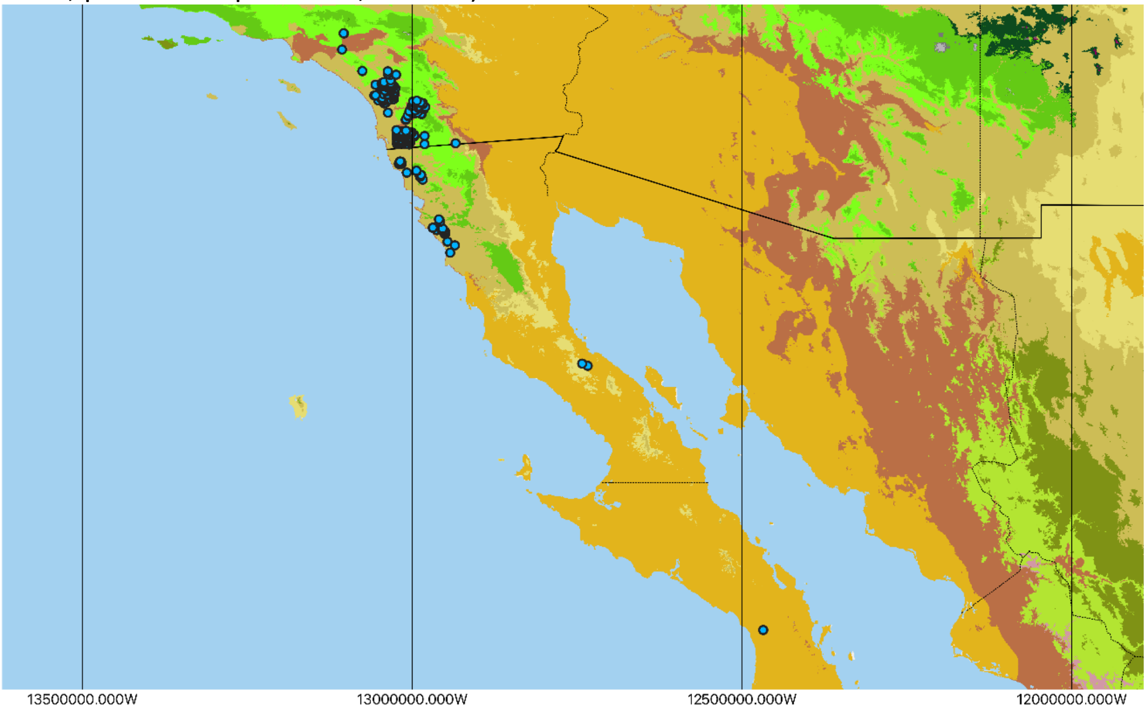

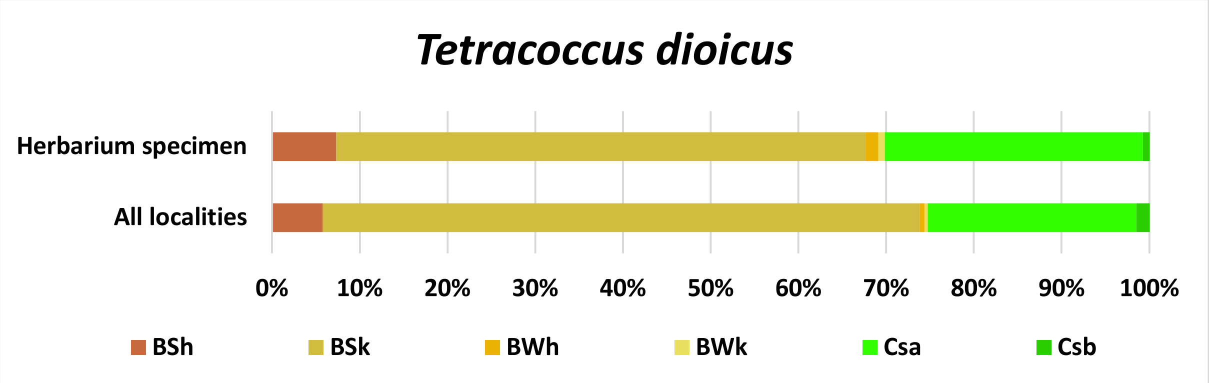

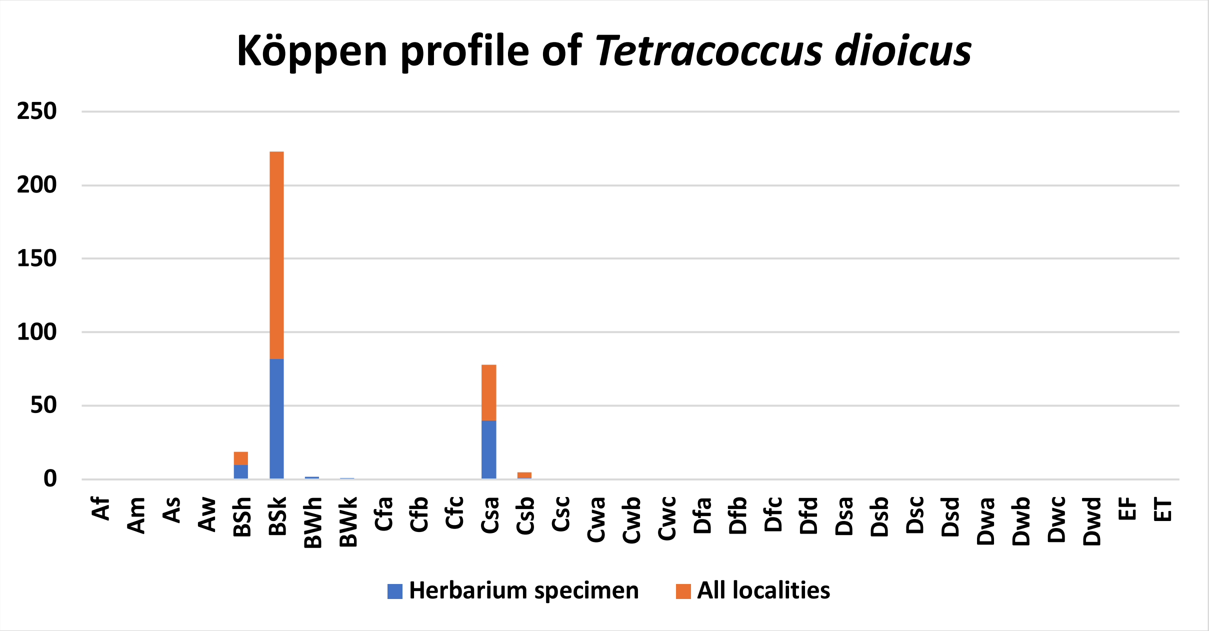

6.2.2. Biome profile, distribution, and climate map – GBIF occurrences *Tetracoccus dioicus*; all specimens (herbarium and human observation) excluding duplicate occurrences (n = 328; preserved specimens, n = 136).

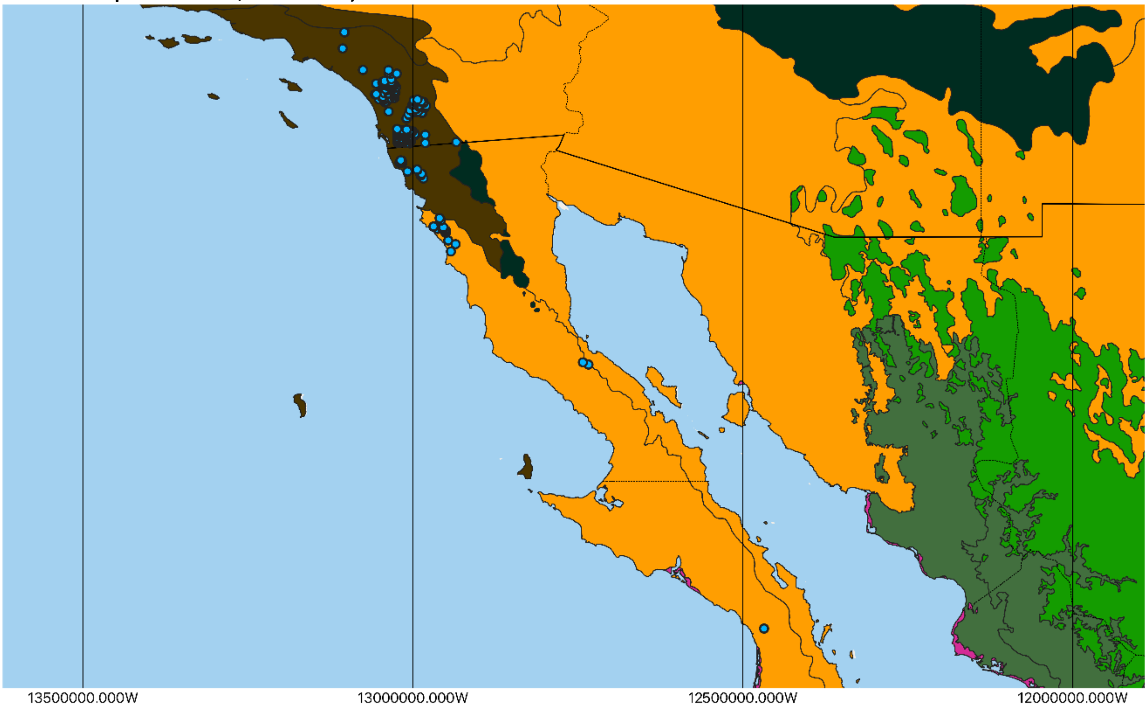

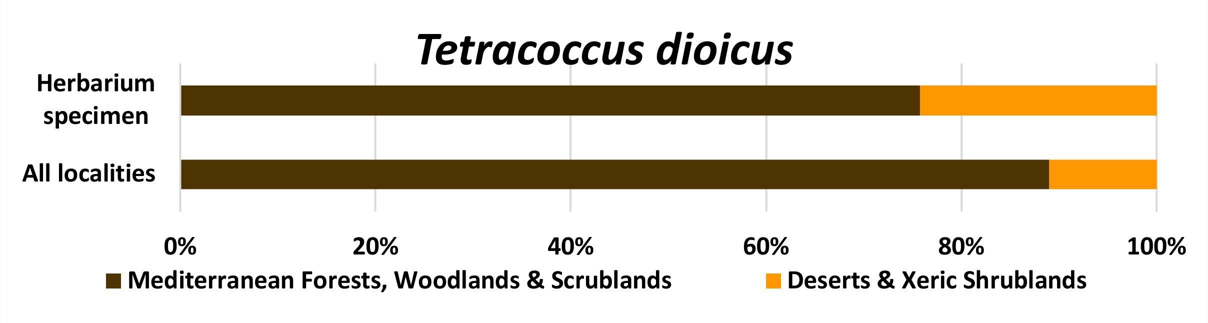

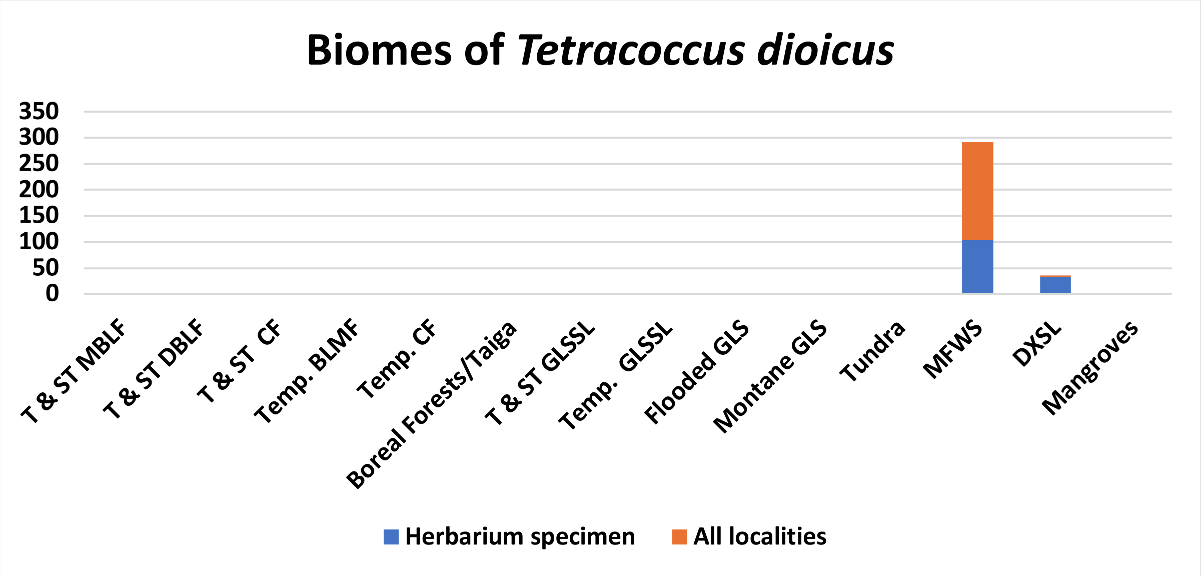

6.2.3. Climate graphs - based on 328 *Tetracoccus dioicus* occurrences in GBIF

6.2.3.1. MMT [°C]

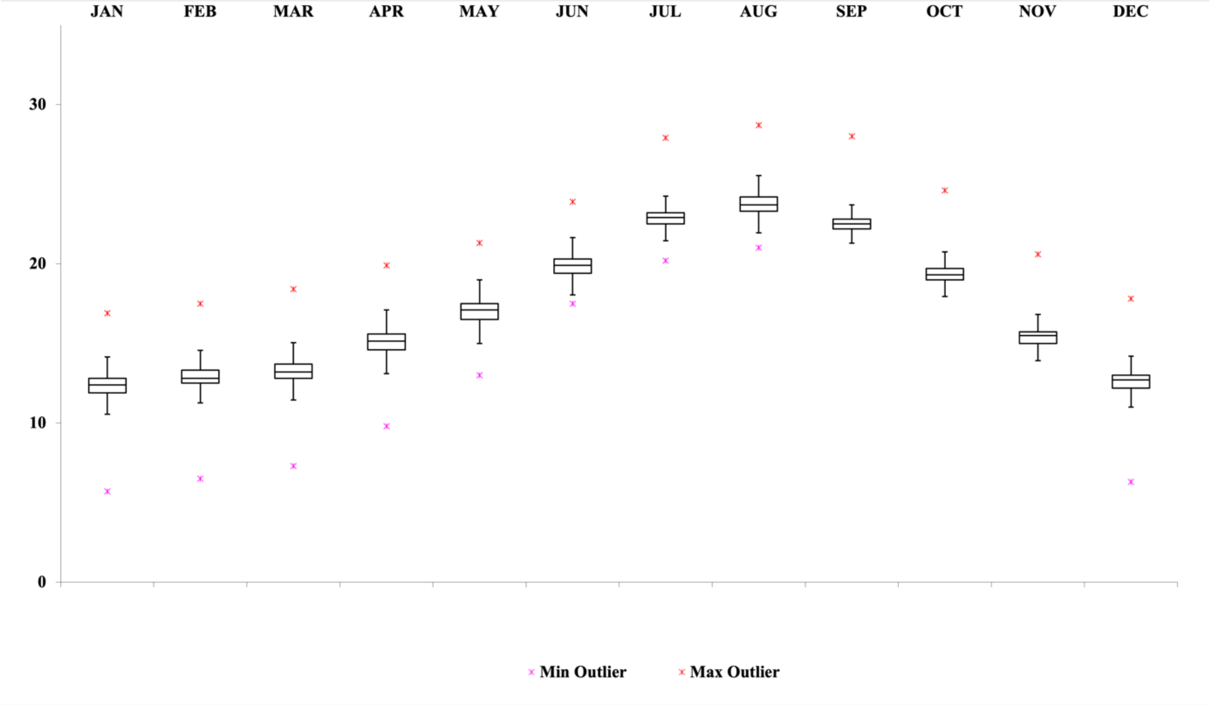

6.2.3.2. MaxMT [°C]

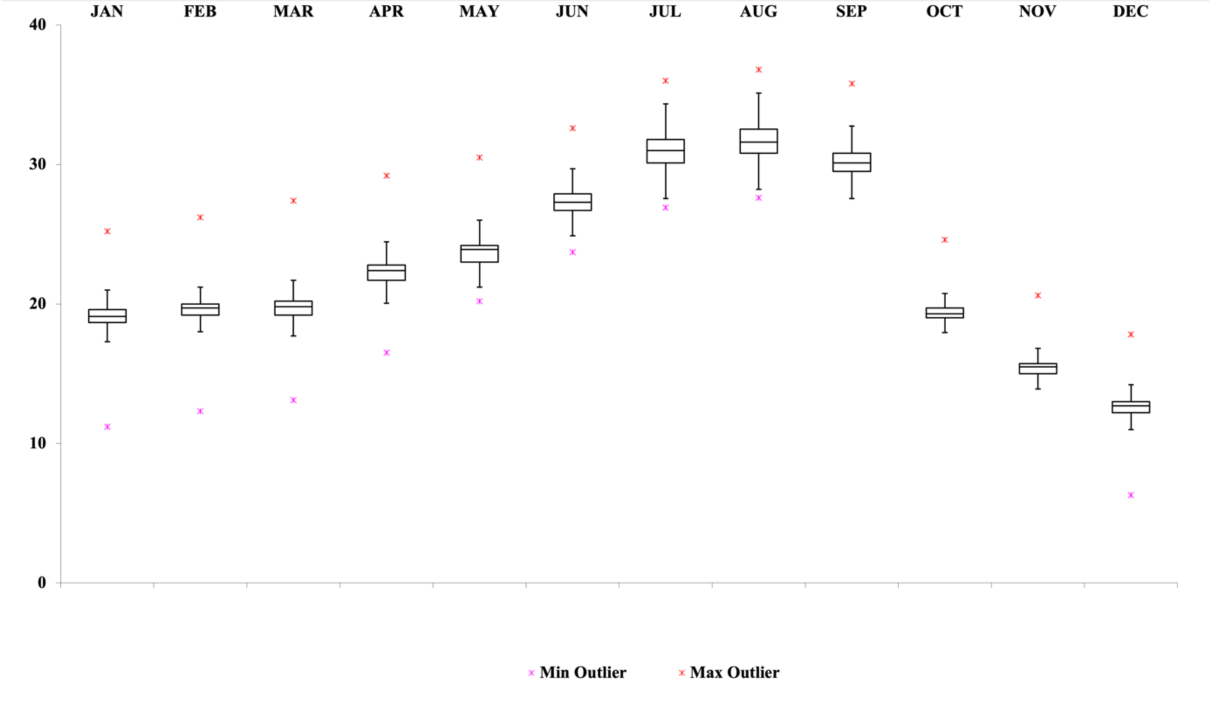

6.2.3.3. MinMT [°C]

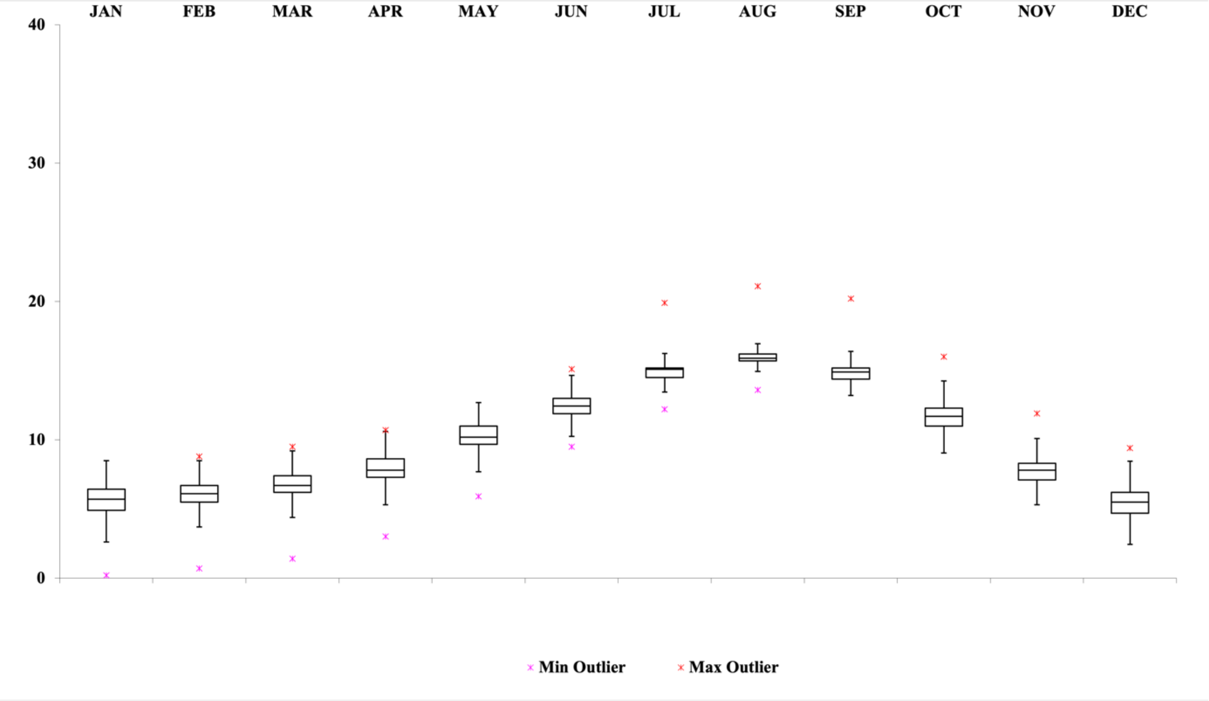

6.2.3.4. MMP [mm]

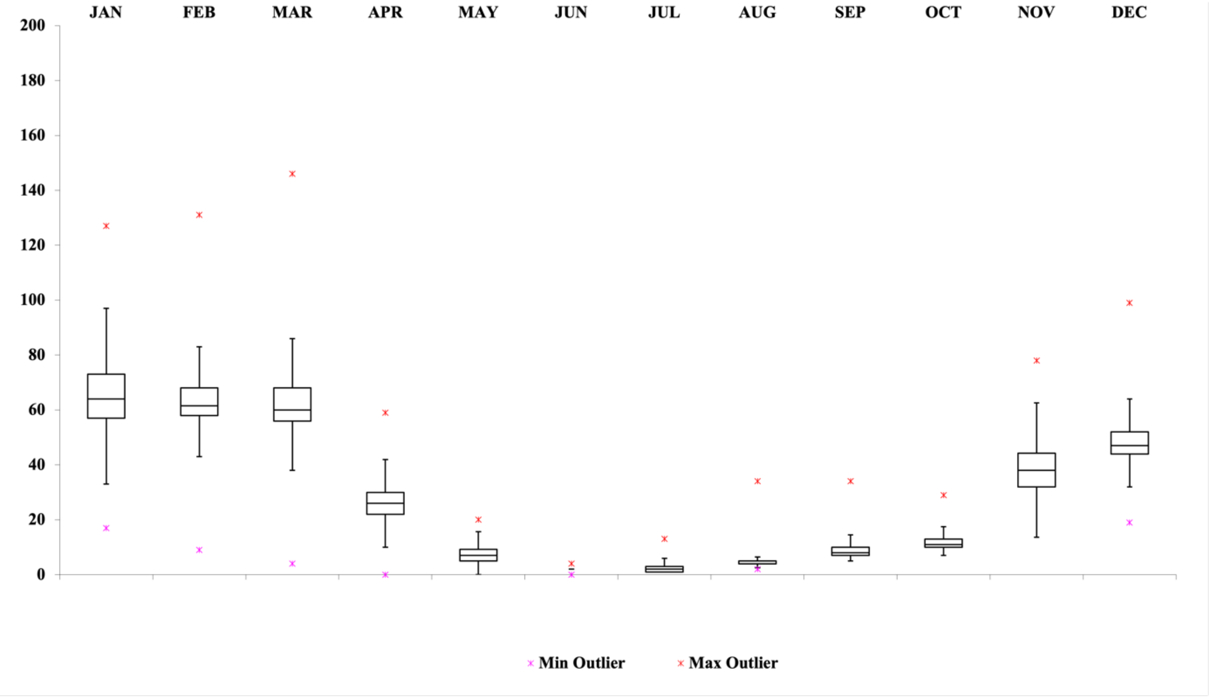

6.3. Species *Tetracoccus fasciculatus* (S.Watson, 1883) Croizat, 1942

6.3.1. Köppen profile, distribution, and climate map – GBIF occurrences of *Tetracoccus fasciculatus*; all specimens (herbarium and human observation) excluding duplicate occurrences (n = 93; preserved specimens, n = 91).

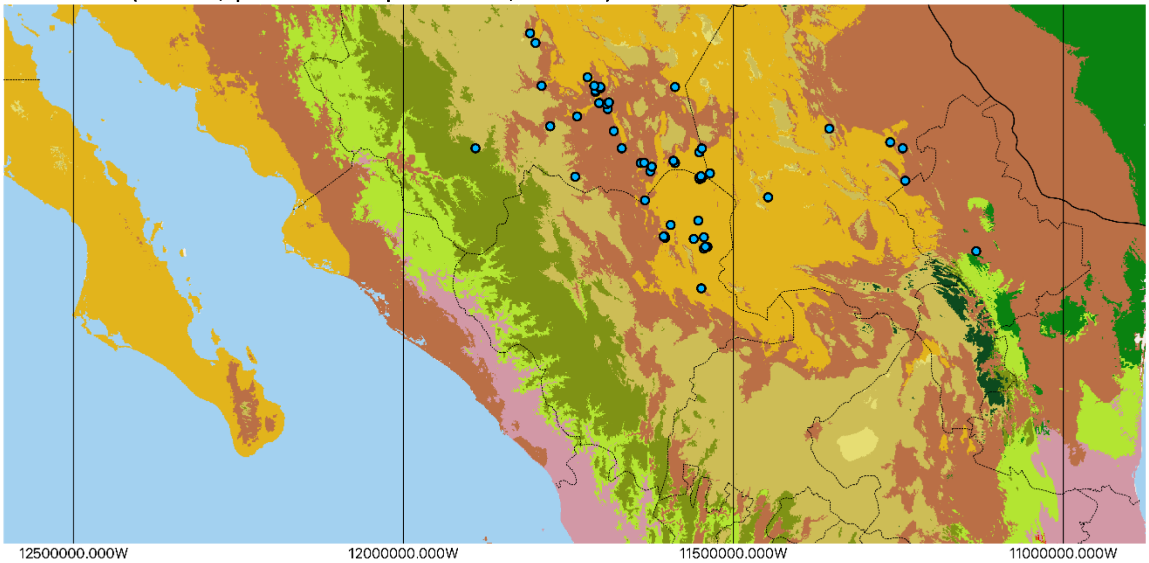

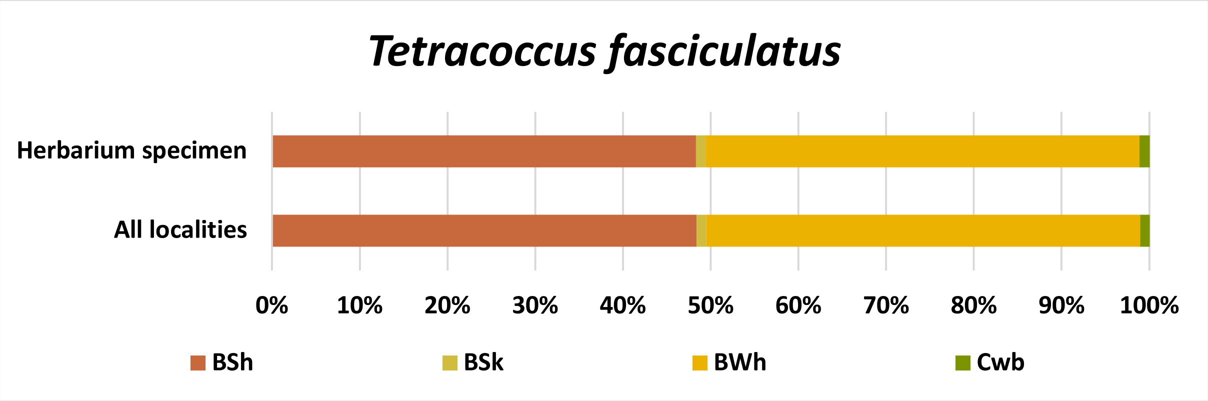

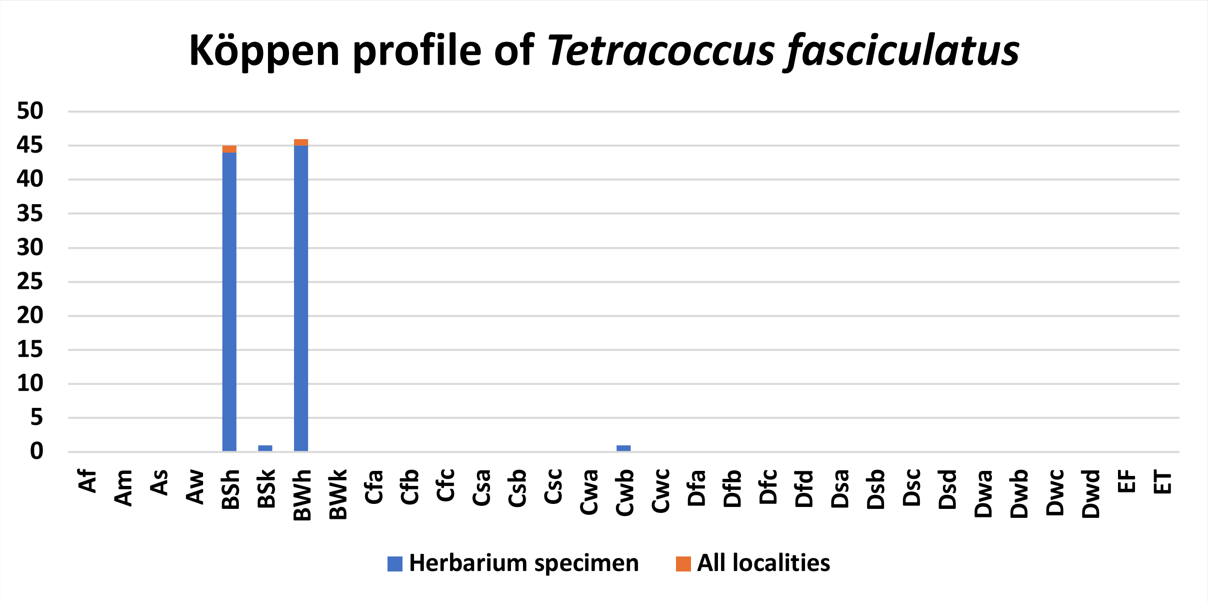

6.3.2. Biome profile, distribution, and climate map – GBIF occurrences of *Tetracoccus fasciculatus*; all specimens (herbarium and human observation) excluding duplicate occurrences (n = 93; preserved specimens, n = 91).

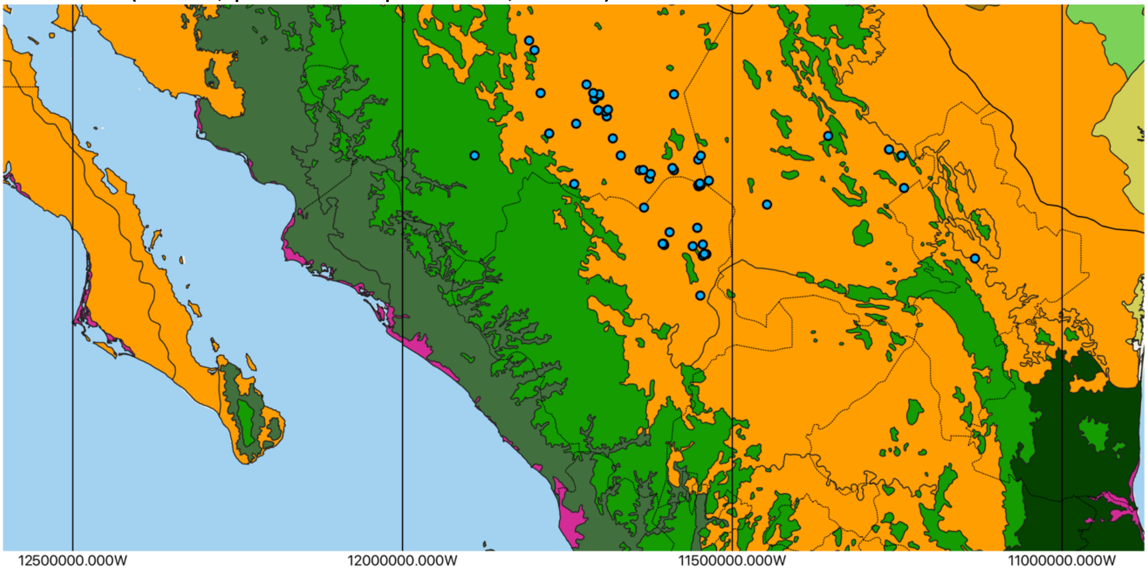

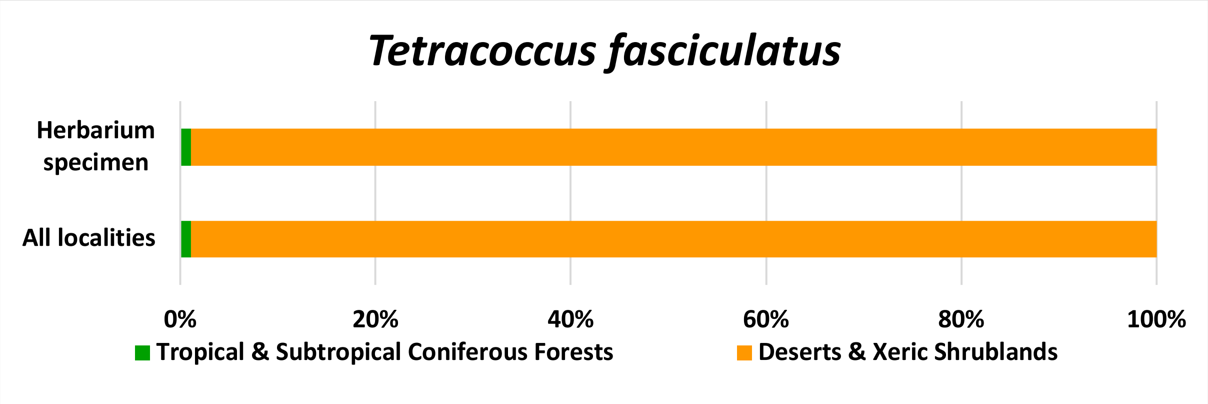

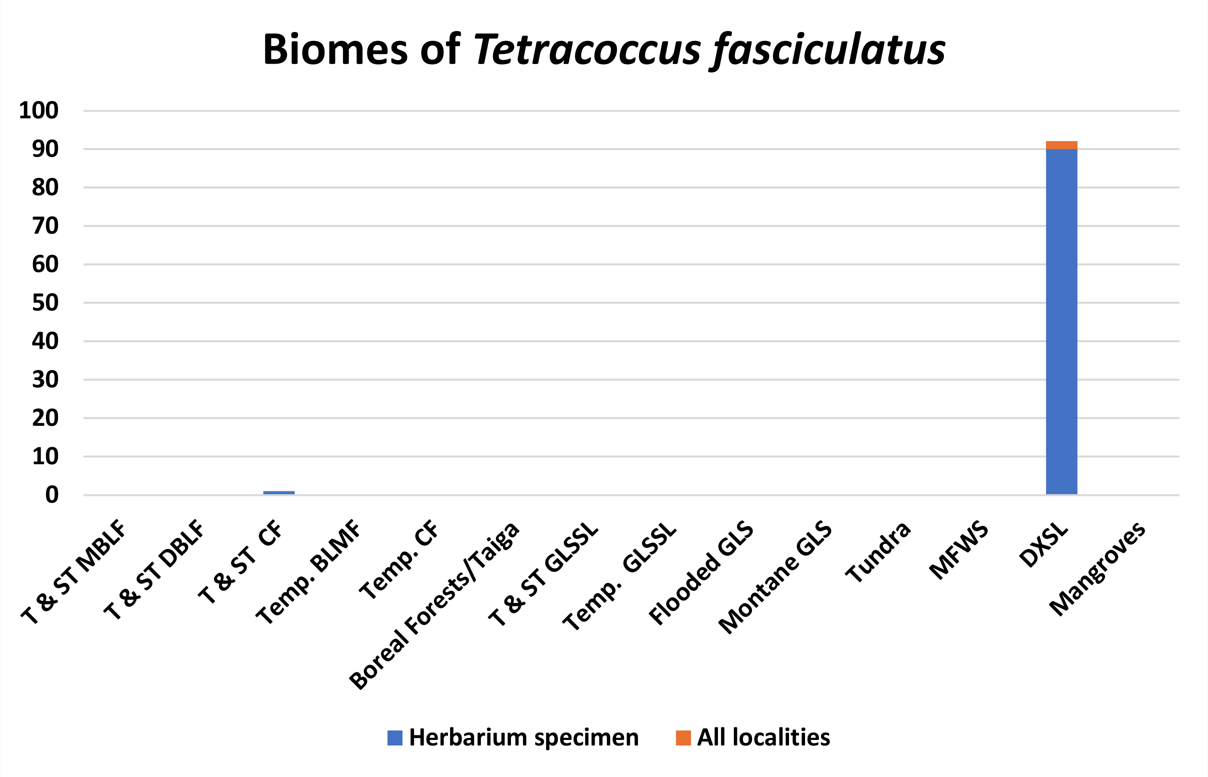

6.3.3. Climate graphs - based on 93 *Tetracoccus fasciculatus* occurrences in GBIF

6.3.3.1. MMT [°C]

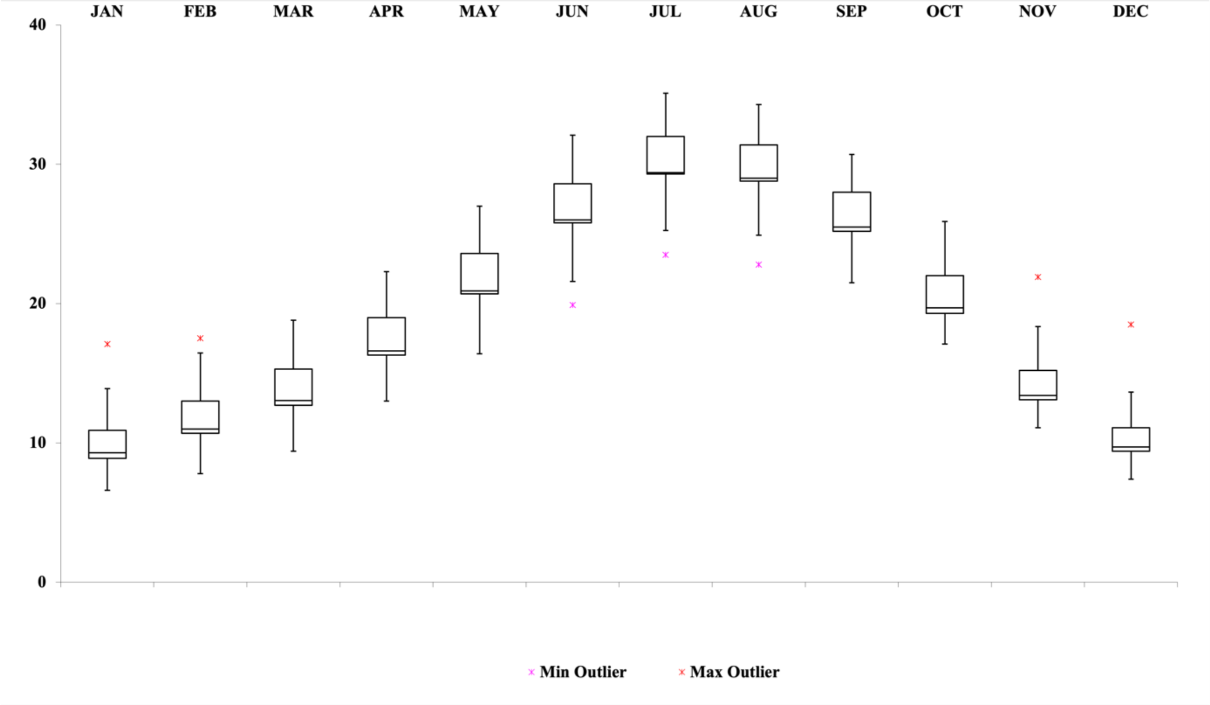

6.3.3.2. MaxMT [°C]

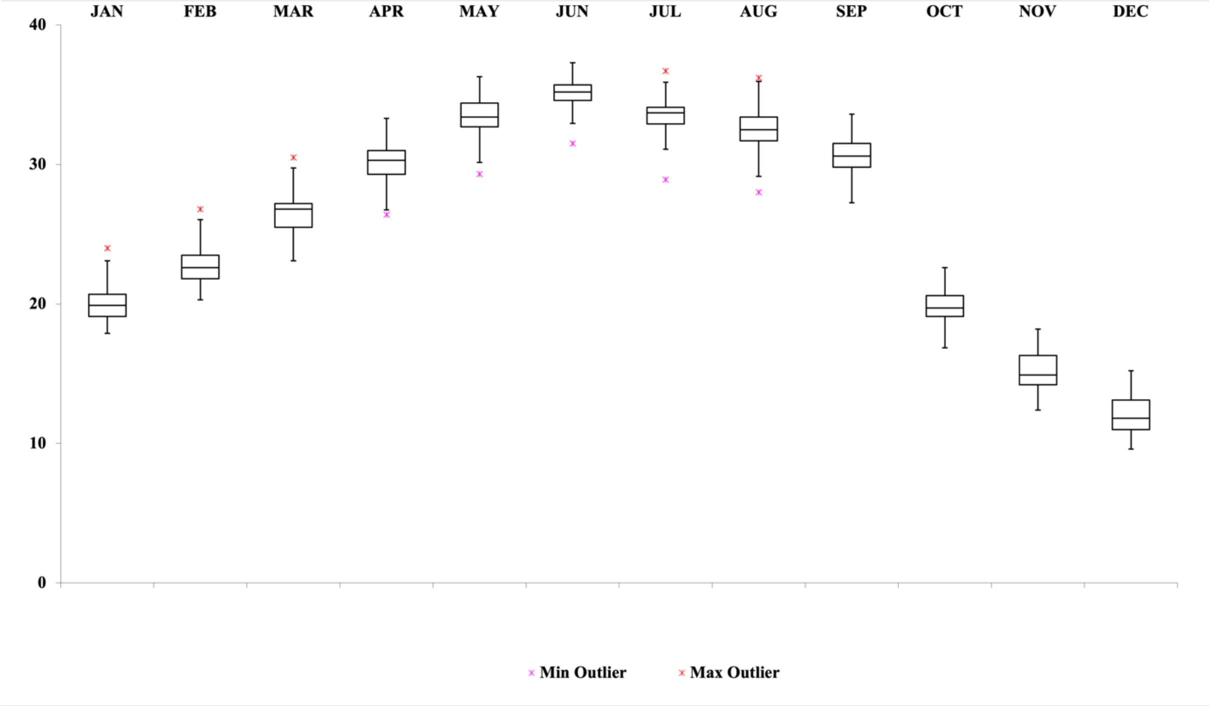

6.3.3.3. MinMT [°C]

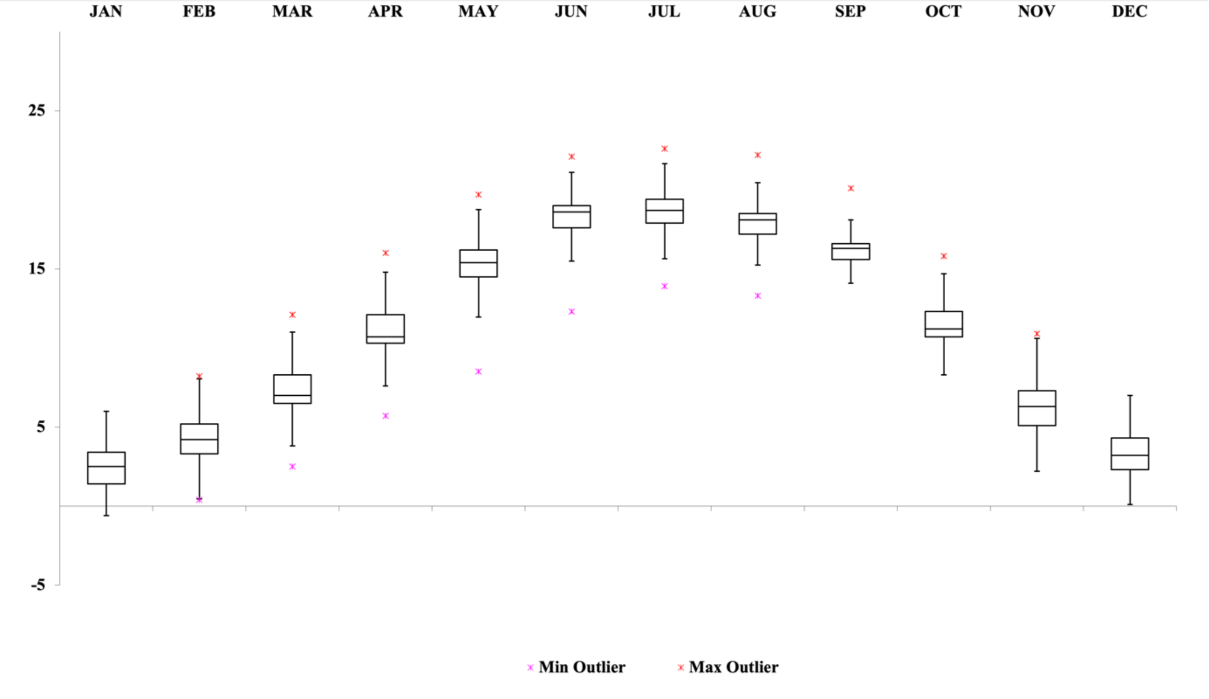

6.3.3.4. MMP [mm]

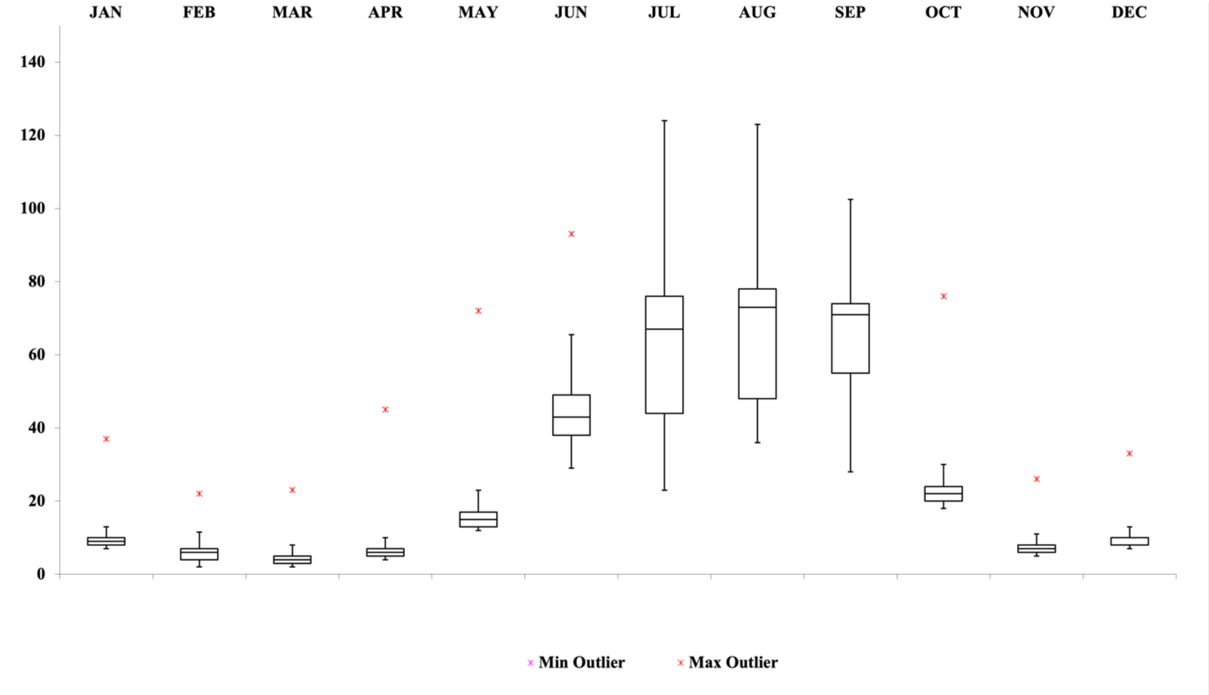

6.4. Species *Tetracoccus hallii* Brandegee, 1906

6.4.1. Köppen profile, distribution, and climate map – GBIF occurrences of *Tetracoccus hallii*; all specimens (herbarium and human observation) excluding duplicate occurrences (n = 566; preserved specimens, n = 213).

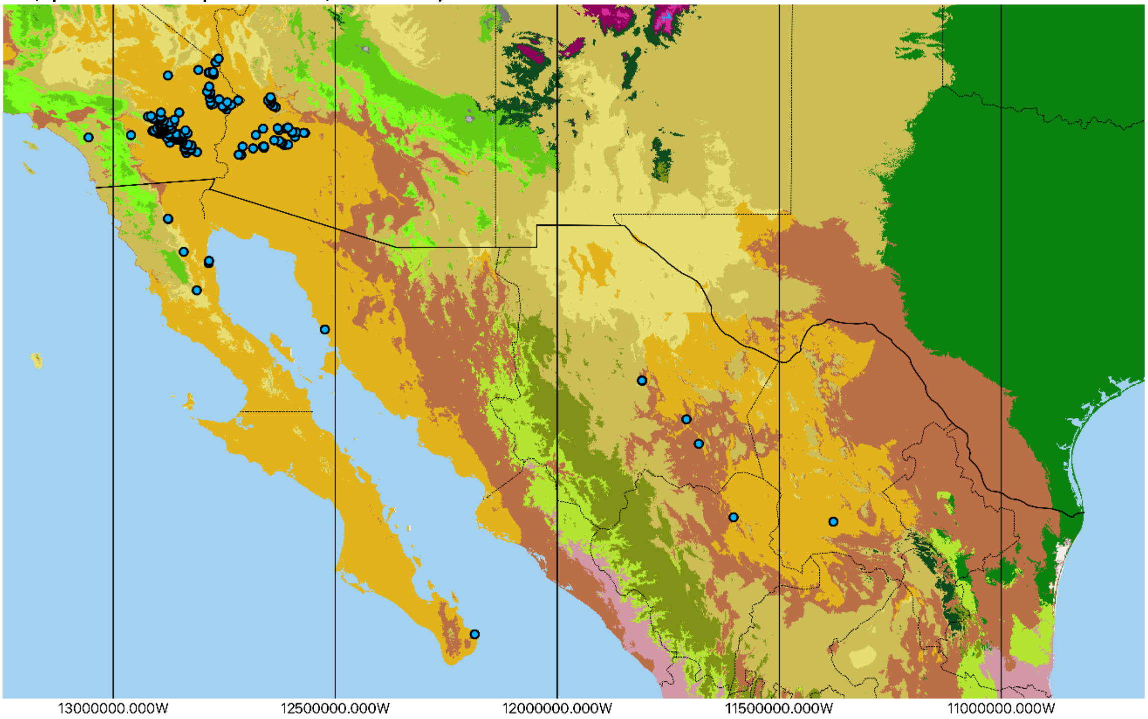

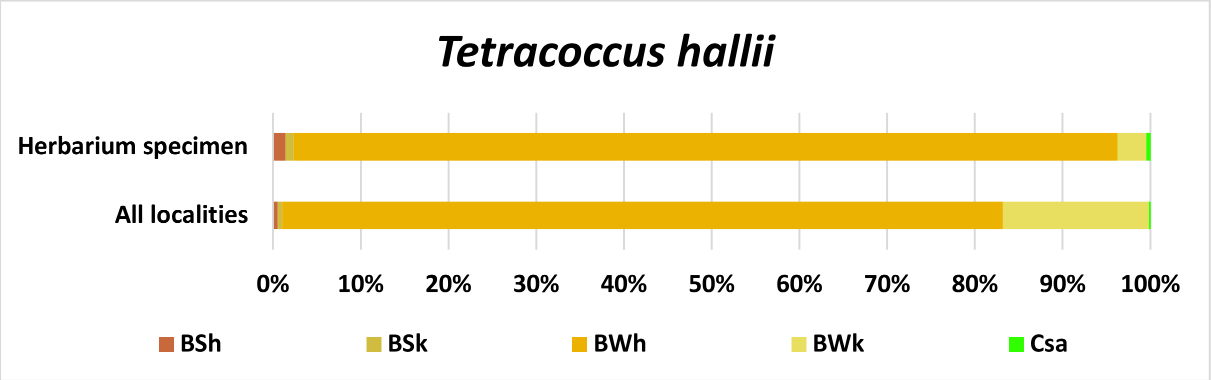

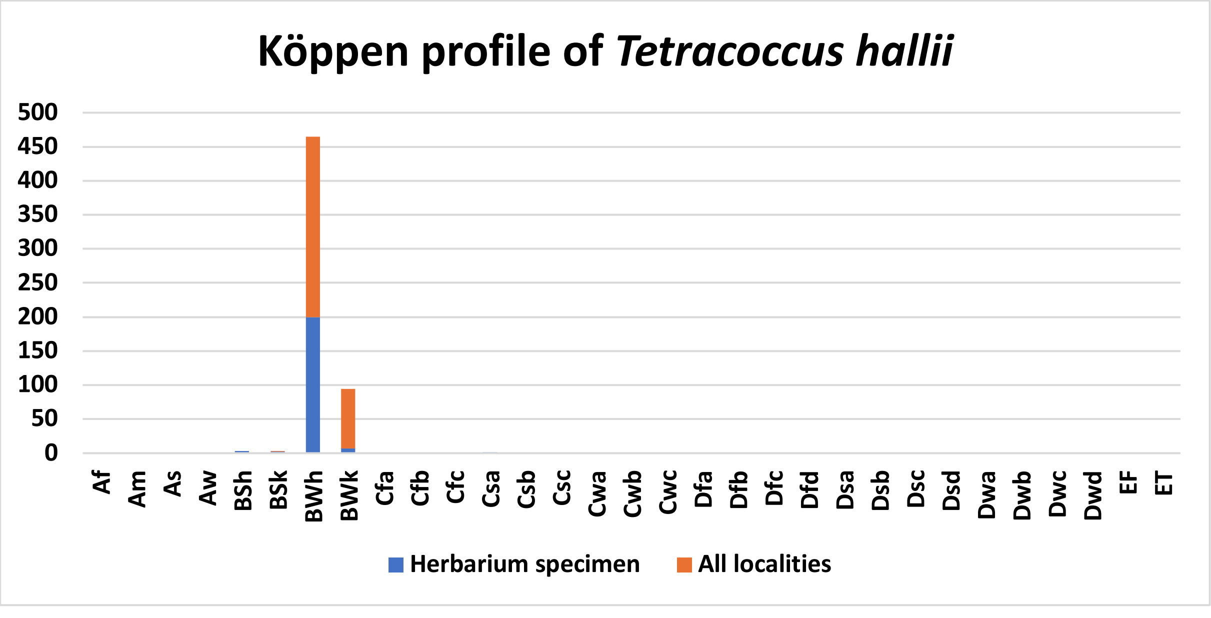

6.4.2. Biome profile, distribution, and climate map – GBIF occurrences of *Tetracoccus hallii*; all specimens (herbarium and human observation) excluding duplicate occurrences (n = 566; preserved specimens, n = 213).

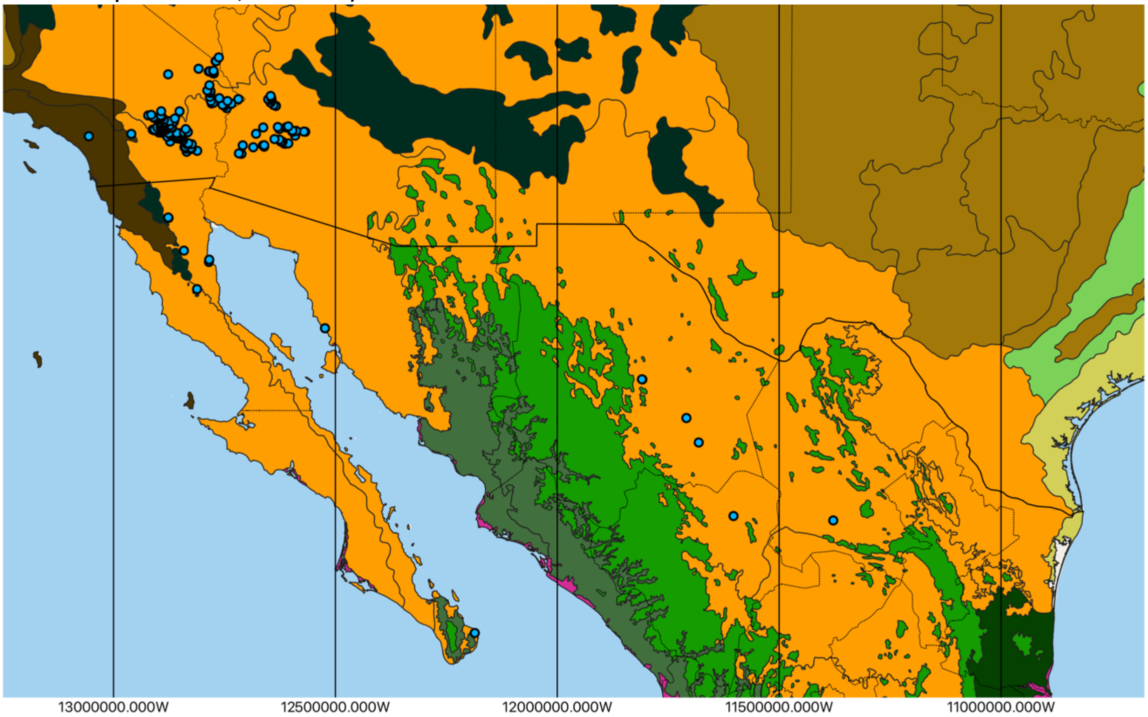

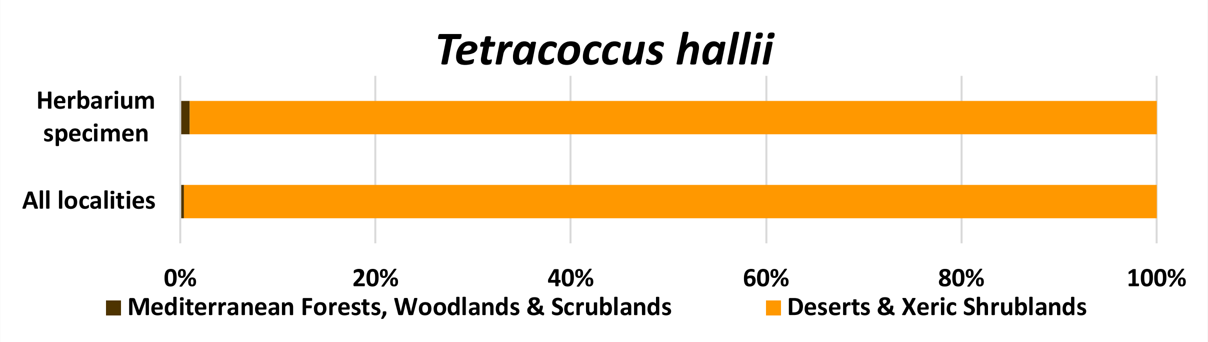

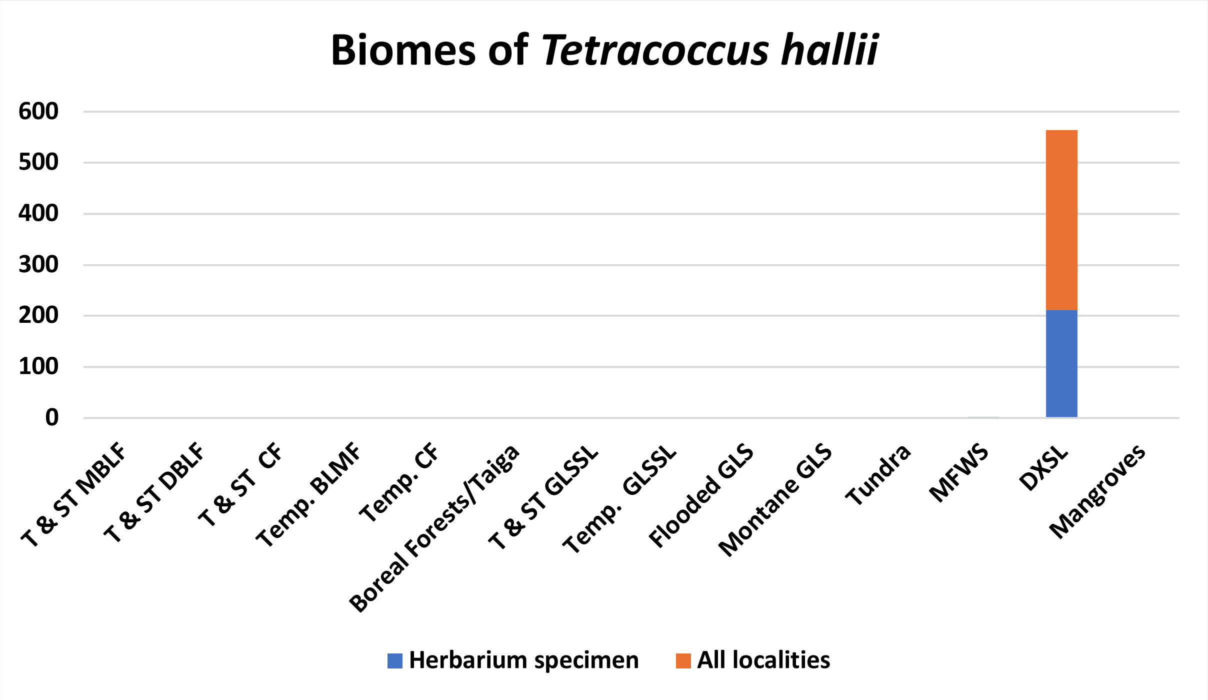

6.4.3. Climate graphs - based on 566 *Tetracoccus hallii* occurrences in GBIF

6.4.3.1. MMT [°C]

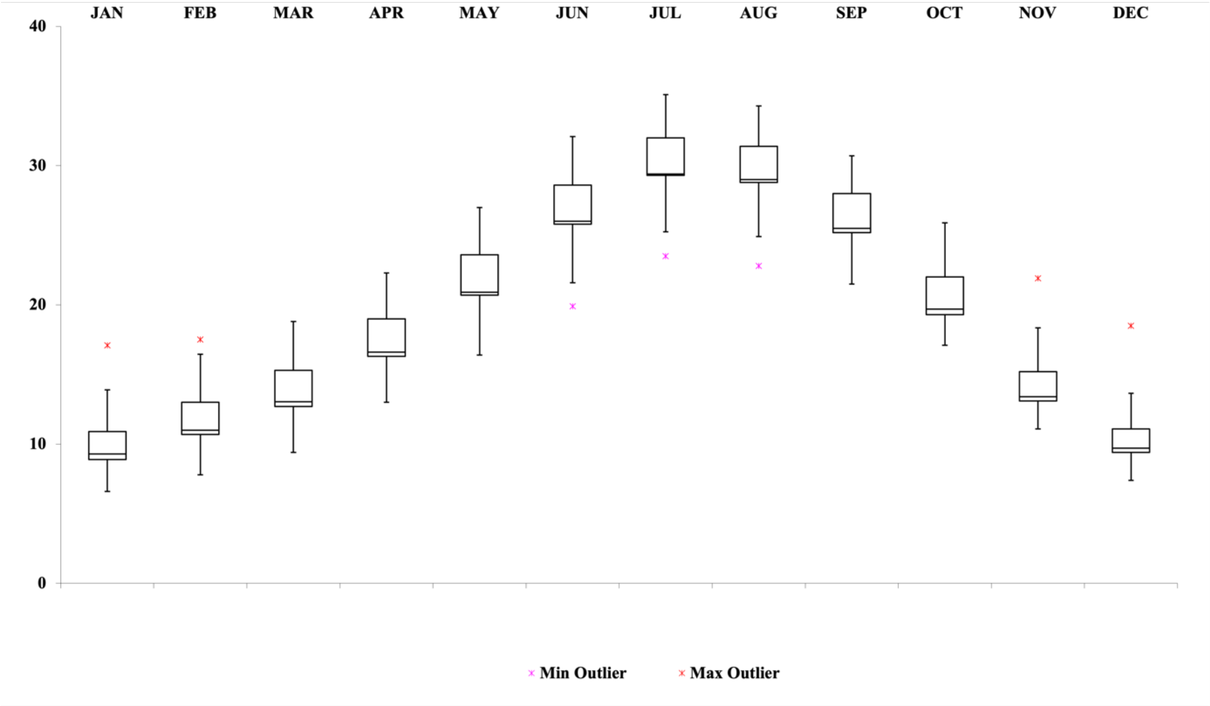

6.4.3.2. MaxMT [°C]

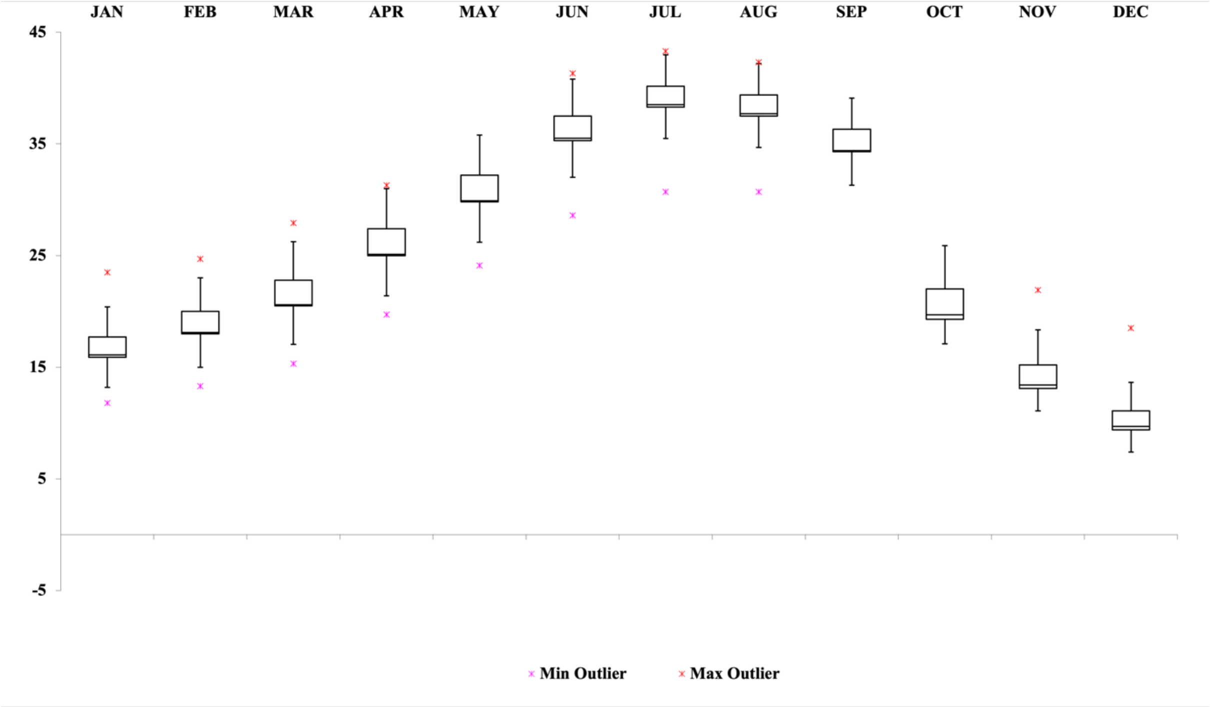

6.4.3.3. MinMT [°C]

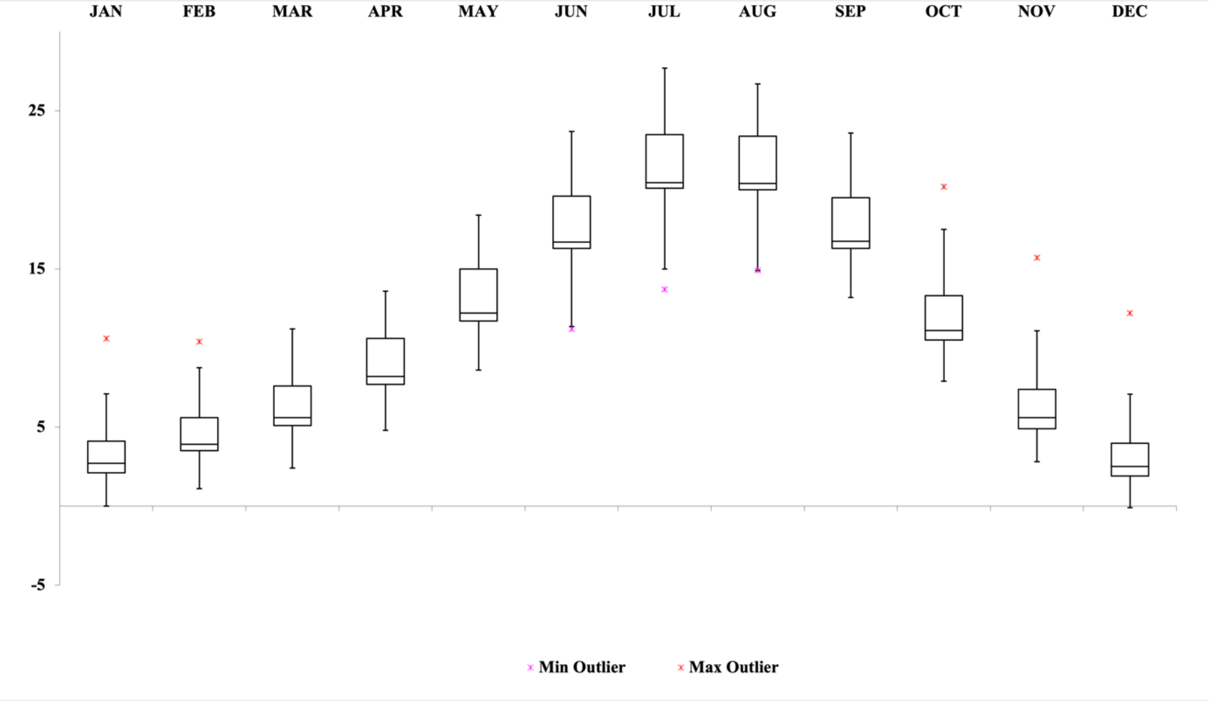

6.4.3.4. MMP [mm]

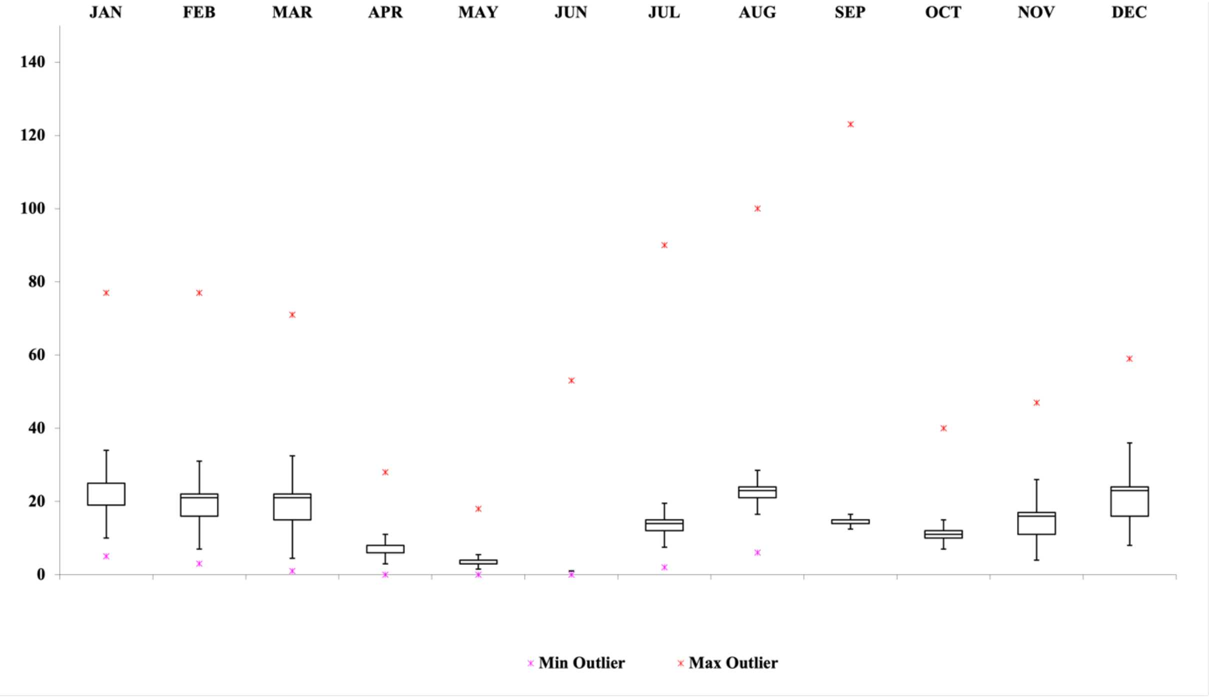

6.5. Species *Tetracoccus ilicifolius* Brandegee, 1906

6.5.1. Köppen profile, distribution, and climate map – GBIF occurrences of *Tetracoccus ilicifolius*; all specimens (herbarium and human observation) excluding duplicate occurrences (n = 37; preserved specimens, n = 23).

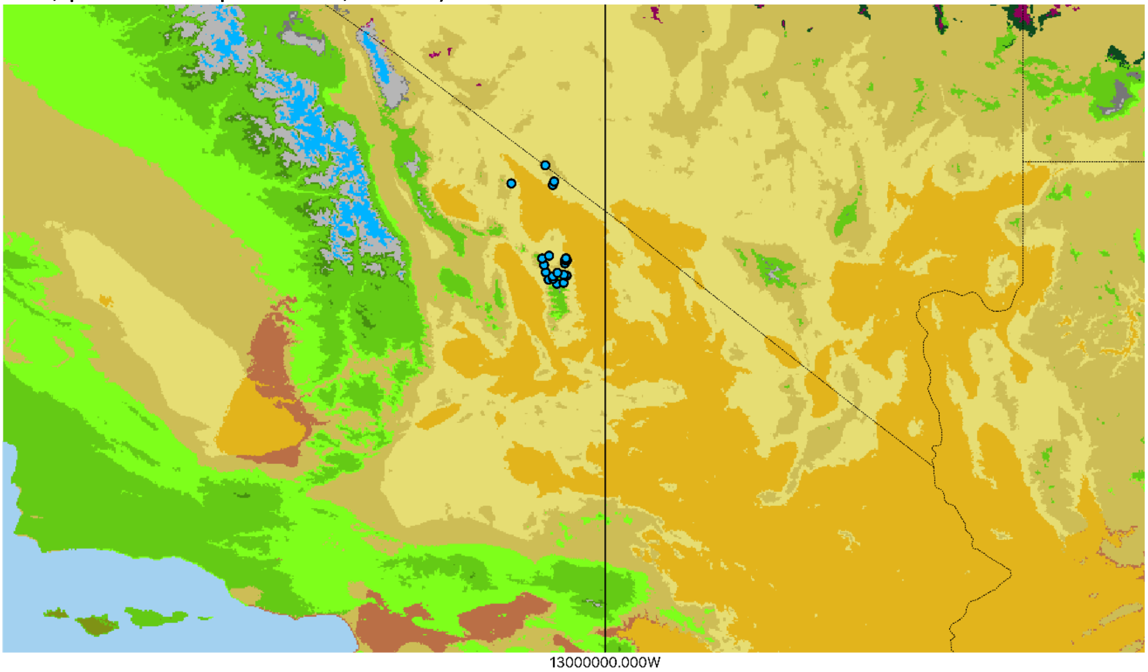

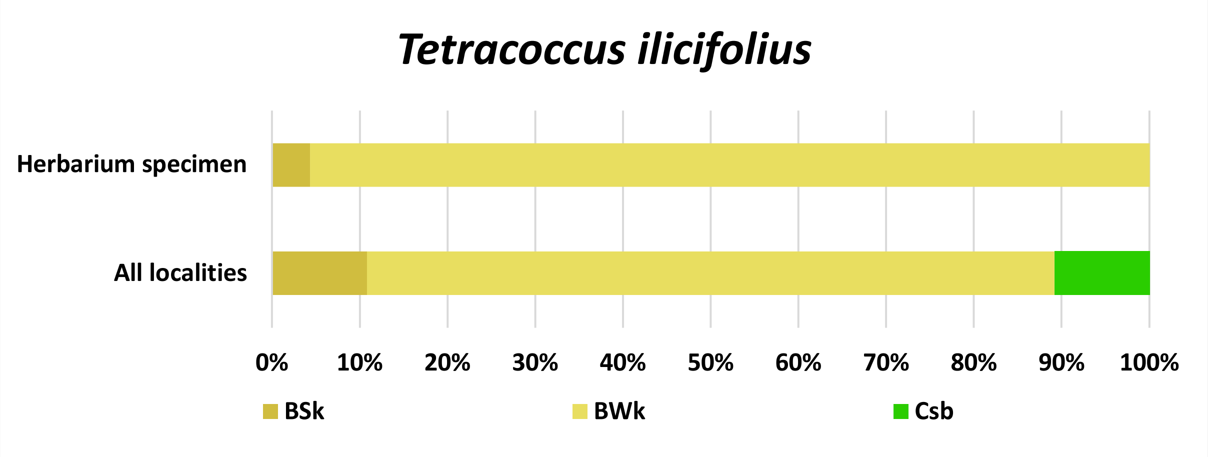

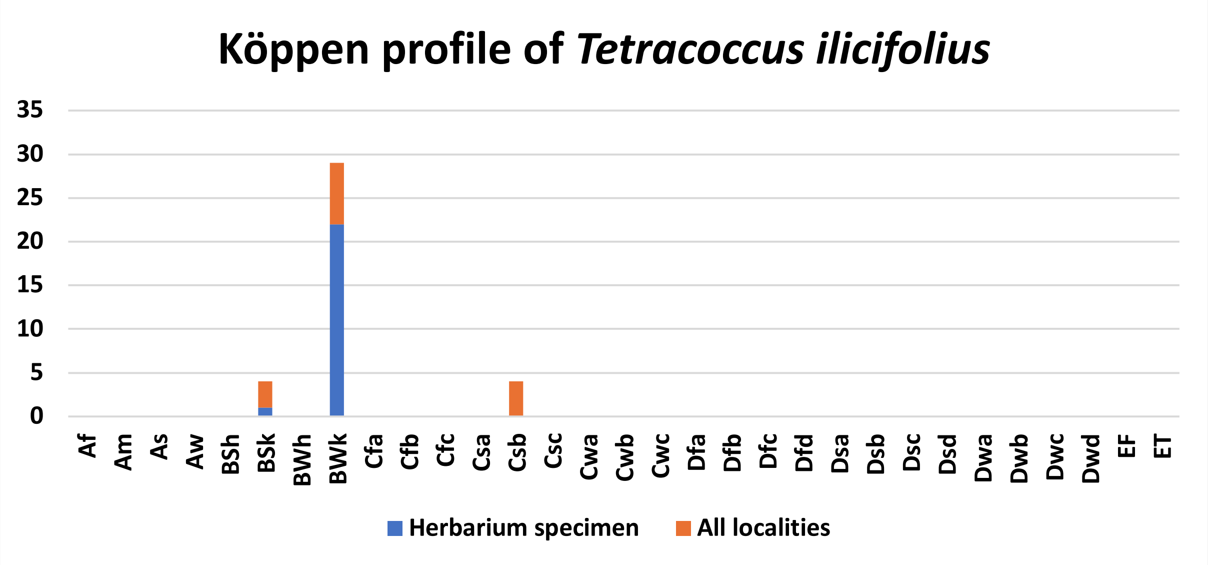

6.5.2. Biome profile, distribution, and climate map – GBIF occurrences of *Tetracoccus ilicifolius*; all specimens (herbarium and human observation) excluding duplicate occurrences (n = 37; preserved specimens, n = 23).

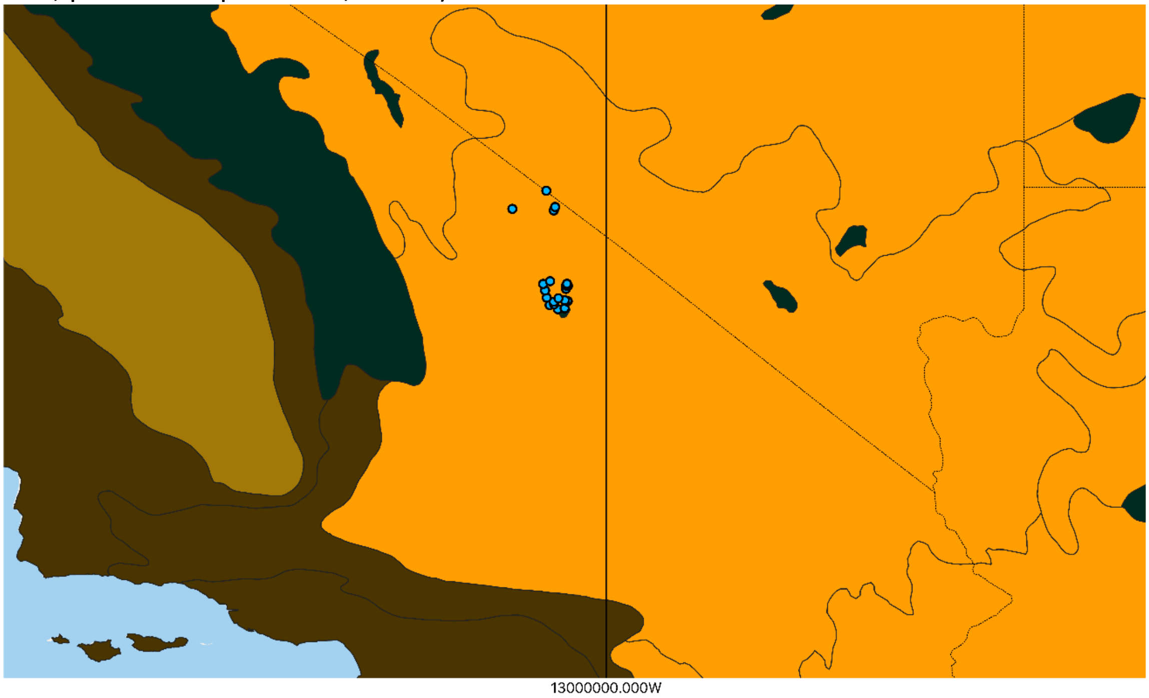

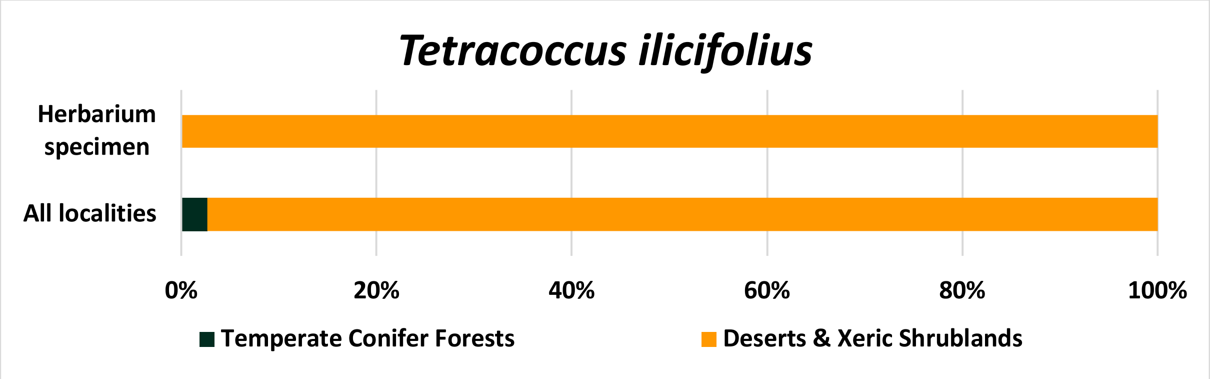

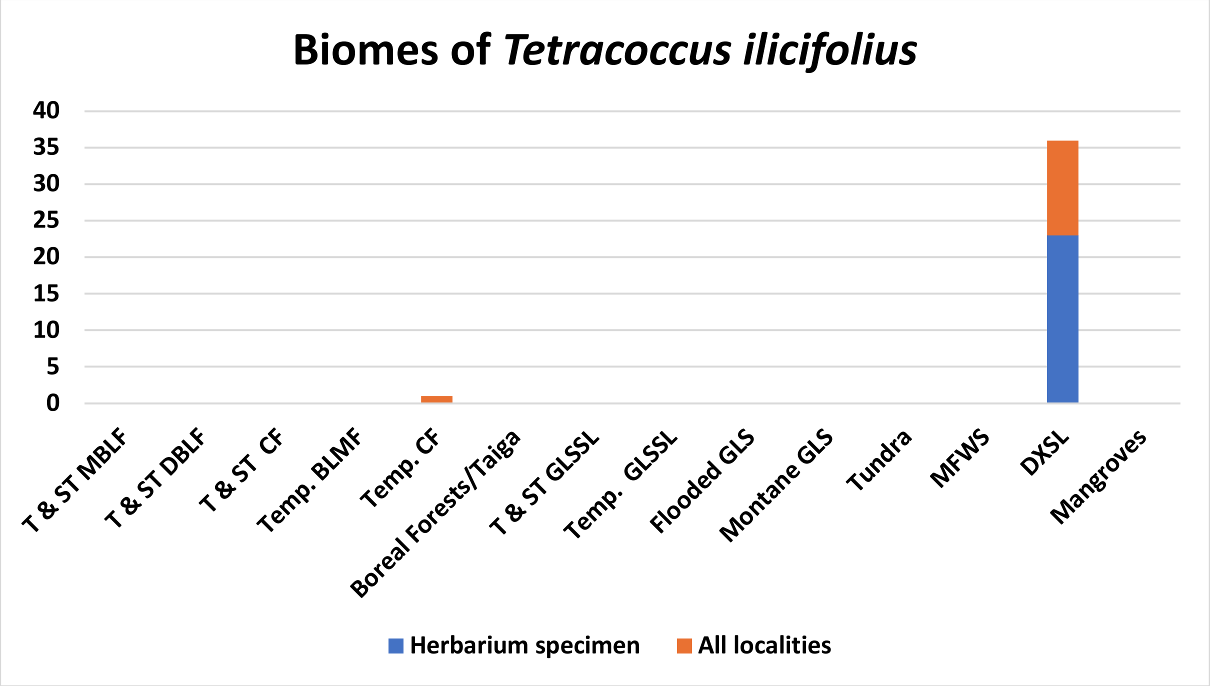

6.5.3. Climate graphs - based on 37 *Tetracoccus ilicifolius* occurrences in GBIF

6.5.3.1. MMT [°C]

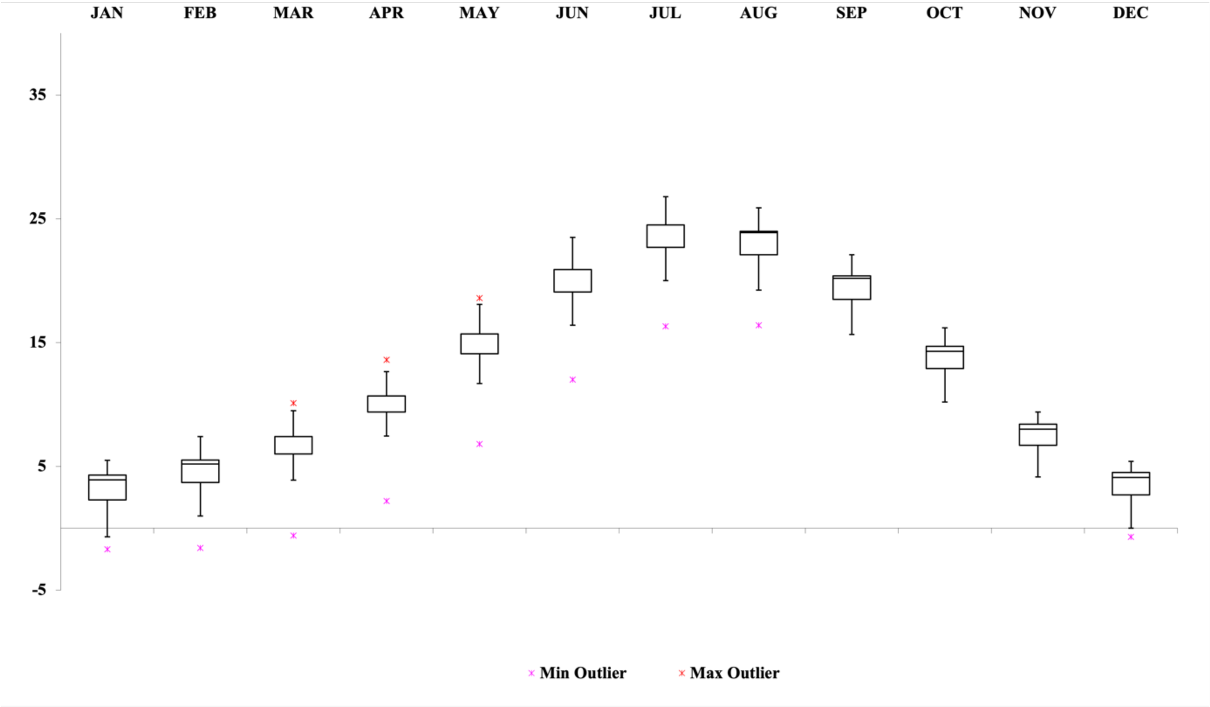

6.5.3.2. MaxMT [°C]

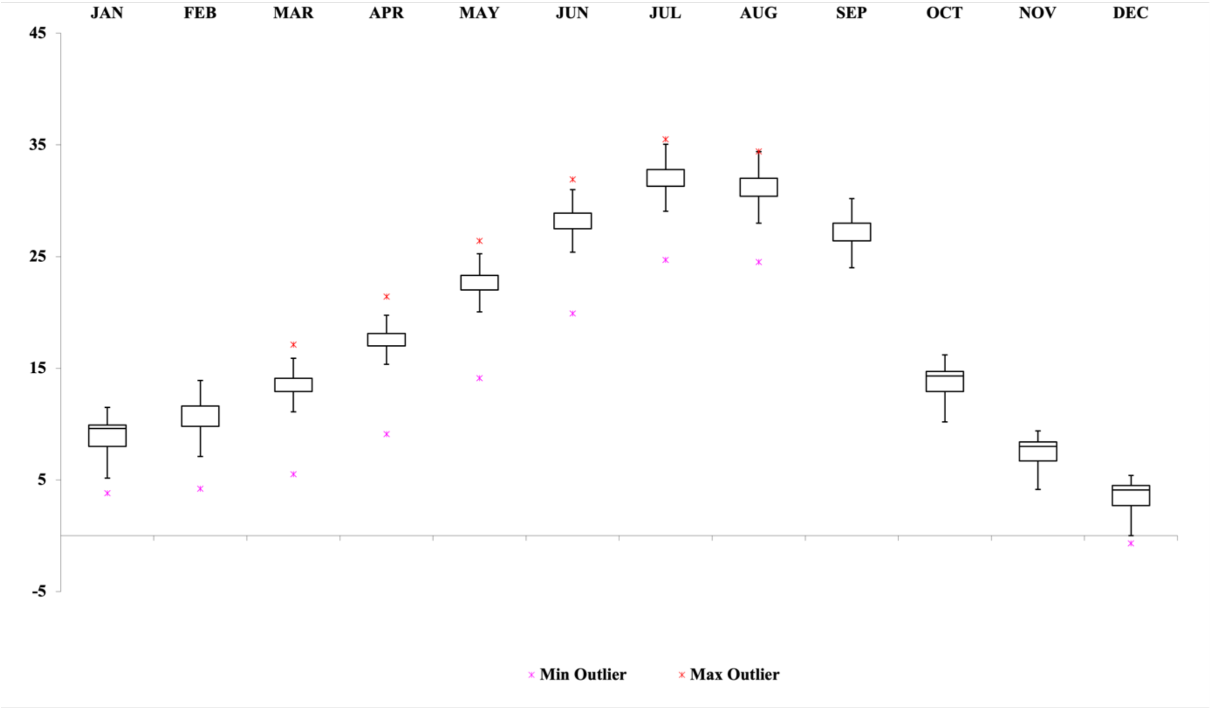

6.5.3.3. MinMT [°C]

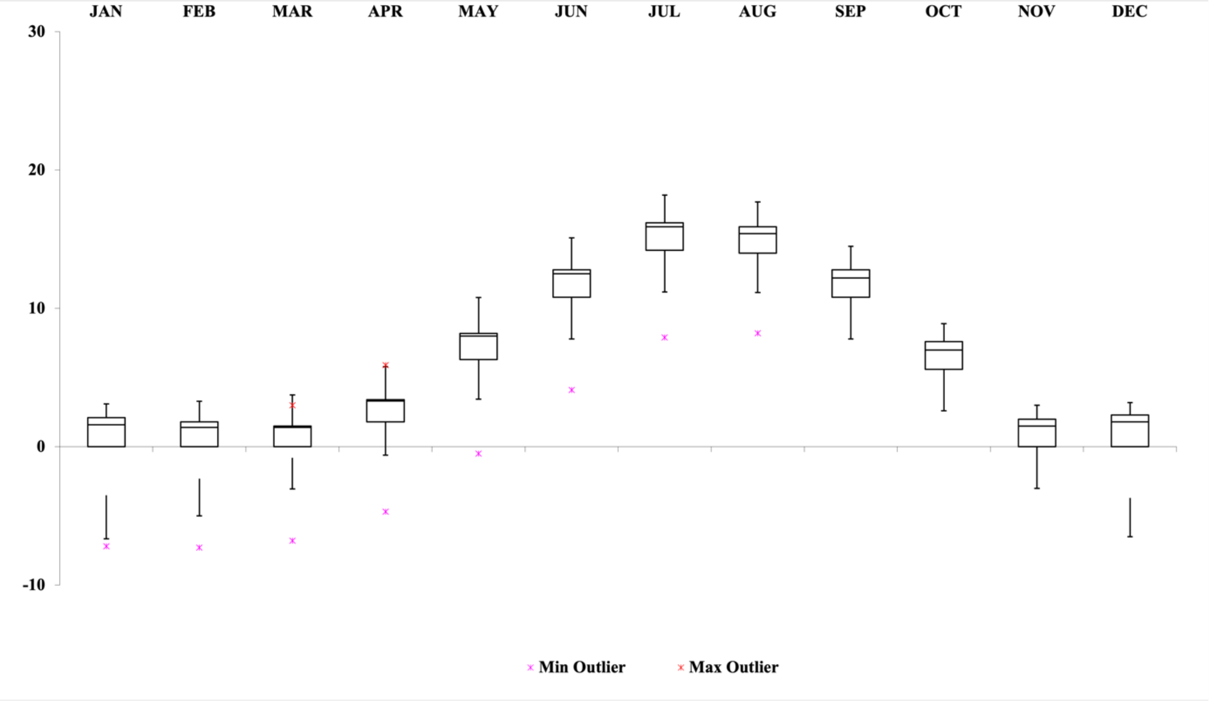

6.5.3.4. MMP [mm]

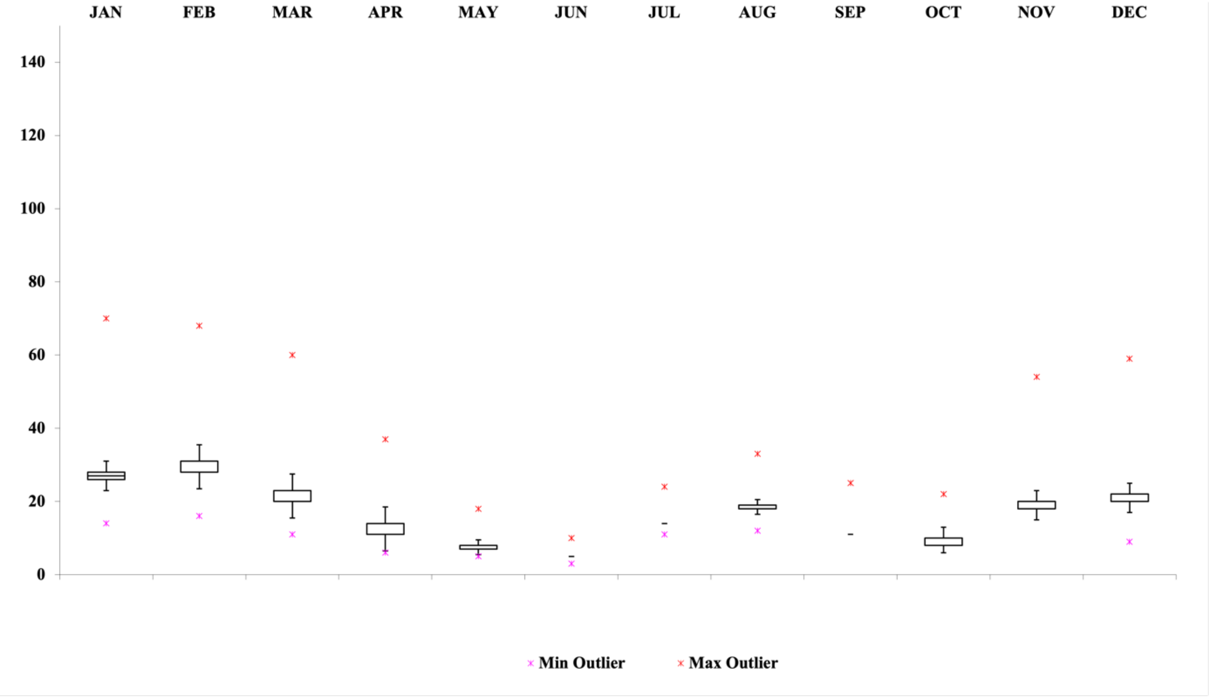

References:

Cui, D., Liang, S., Wang, D., and Liu, Z. 2021. A 1 km global dataset of historical (1979–2013) and future (2020–2100) Köppen–Geiger climate classification and bioclimatic variables. Earth Syst. Sci. Data, 13, 5087–5114. https://doi.org/10.5194/essd-13-5087-2021

Kottek, M., Grieser, J., Beck, C., Rudolf, B., and Rubel, F. 2006. World map of the Köppen-Geiger climate classification updated. Meteorol. Z., 15, 259–263.

Olson, D.M., Dinerstein, E., Wikramanayake, E.D., Burgess, N.D., Powell, G.V.N., Underwood, E.C., D'Amico, J.A., Itoua, I., Strand, H.E., Morrison, J.C., Loucks, C.L., Allnutt, T.F., Ricketts, T.H., Kura, Y., Lamoreux, J.F., Wettengel, W.W., Hedao, P., and Kassem, K.R. 2001 Terrestrial ecosystems of the world: A new map of life on Earth. *BioScience*, 51, 933-938.

Peel, M. C., Finlayson, B. L., and McMahon, T. A. 2007. Updated world map of the Köppen-Geiger climate classification, Hydrol. Earth Syst. Sci., 11, 1633–1644, https://doi.org/10.5194/hess-11-1633-2007.

Rubel, F., Brugger, K., Haslinger, K., and Auer, I. 2017. The climate of the European Alps: Shift of very high resolution Köppen- Geiger climate zones 1800–2100, Meteorol. Z., 26, 115–125, https://doi.org/10.1127/metz/2016/0816.

GBIF Datasets:
Hyaenachne globosa, GBIF Occurrences (downloaded, 12 September 2024), https://doi.org/10.15468/dl.52hh3x

Picrodendron baccatum, GBIF Occurrences (downloaded, 5 November 2024), https://doi.org/10.15468/dl.ee6dmh

Prianhea, GBIF Occurrences (downloaded, 12 September 2024), https://doi.org/10.15468/dl.eeaucq

Tetracoccus, GBIF Occurrences (downloaded, 5 November 2024),https://doi.org/10.15468/dl.gzmzaf
